# Use of *Aleuria alantia* Lectin Affinity Chromatography to Enrich Candidate Biomarkers from the Urine of Patients with Bladder Cancer

**DOI:** 10.3390/proteomes3030266

**Published:** 2015-09-03

**Authors:** Sarah R. Ambrose, Naheema S. Gordon, James C. Goldsmith, Wenbin Wei, Maurice P. Zeegers, Nicholas D. James, Margaret A. Knowles, Richard T. Bryan, Douglas G. Ward

**Affiliations:** 1 School of Cancer Sciences, University of Birmingham, Birmingham B15 2TT, UK; E-Mails: SRA295@student.bham.ac.uk (S.R.A.); n.gordon@bham.ac.uk (N.S.G.); jgoldsmith38@googlemail.com (J.C.G.); w.wei@bham.ac.uk (W.W.); m.zeegers@maastrichtuniversity.nl (M.P.Z.); r.t.bryan@bham.ac.uk (R.T.B.); 2 Department of Complex Genetics, NUTRIM School of Nutrition and Translational Research in Metabolism, Maastricht University Medical Centre, Maastricht 6200 MD, The Netherlands; 3 Clinical Trials Unit, University of Warwick, Coventry CV4 7AL, UK; E-Mail: N.D.James@warwick.ac.uk; 4 Section of Experimental Oncology, Leeds Institute of Cancer and Pathology, St James’s’ University Hospital, Beckett Street, Leeds LS9 7TF, UK; E-Mail: M.A.Knowles@leeds.ac.uk

**Keywords:** bladder cancer, urine, biomarker, lectin, glycoproteome

## Abstract

Developing a urine test to detect bladder tumours with high sensitivity and specificity is a key goal in bladder cancer research. We hypothesised that bladder cancer-specific glycoproteins might fulfill this role. Lectin-ELISAs were used to study the binding of 25 lectins to 10 bladder cell lines and serum and urine from bladder cancer patients and non-cancer controls. Selected lectins were then used to enrich glycoproteins from the urine of bladder cancer patients and control subjects for analysis by shotgun proteomics. None of the lectins showed a strong preference for bladder cancer cell lines over normal urothlelial cell lines or for urinary glycans from bladder cancer patients over those from non-cancer controls. However, several lectins showed a strong preference for bladder cell line glycans over serum glycans and are potentially useful for enriching glycoproteins originating from the urothelium in urine. *Aleuria alantia* lectin affinity chromatography and shotgun proteomics identified mucin-1 and golgi apparatus protein 1 as proteins warranting further investigation as urinary biomarkers for low-grade bladder cancer. Glycosylation changes in bladder cancer are not reliably detected by measuring lectin binding to unfractionated proteomes, but it is possible that more specific reagents and/or a focus on individual proteins may produce clinically useful biomarkers.

## 1. Introduction

Urothelial bladder cancer is the fourth most common cancer in men and ninth most common cancer in women in western societies [1]. This highly heterogeneous disease presents as a spectrum from low-grade non-invasive tumours with a good prognosis but high recurrence rate through to high-grade muscle invasive tumours with a very poor prognosis. Low grade tumours tend to have a near-normal karyotype with few genomic rearrangements and often have activating mutations in *FGFR3* and the MAPK pathway, whereas high-grade tumours typically have inactivating mutations in *TP53* and/or other tumour suppressor genes and multiple chromosomal aberrations [2]. Algorithms based on clinicopathological factors can be used to guide treatment, which ranges from transurethral resection and surveillance for recurrence for low-risk disease through to cystectomy and systemic chemotherapy for muscle-invasive and metastatic disease [3].

Bladder tumours are typically detected by flexible cystoscopy, a burdensome and resource-intensive procedure. Patients undergoing surveillance for bladder cancer will require this procedure at regular intervals for many years [4]. There is thus a need for a urine or blood-based test to reduce reliance on cystoscopy. Despite extensive research, most candidate urinary biomarkers for bladder cancer do not show sufficient sensitivity and specificity to replace cystoscopy [5]. Most of the proposed biomarkers are particularly poor at detecting low-grade NMIBC. Indeed, no urinary biomarkers have been validated as having sufficient sensitivity and specificity to be widely adopted in clinical practice [5].

A number of urinary biomarkers have been proposed for the detection of bladder cancer including tests based on miRNA [6], RNA [7], DNA [8], metabolites [9] and proteins [10]. The latter may be measured in exfoliated cells (e.g., ImmunoCyt^®^) or as soluble proteins in the urine e.g., NMP22 and BTA. Unfortunately, none of these tests are both highly specific and sensitive for early stage and low grade disease. Nucleic acid tests based on DNA methylation and mutations have the advantage over other biomarkers in identifying the presence of a disease-specific variant of the molecule that is being detected, rather than the total level of a molecule which may be released from both cancer and normal cells. Because bladder cancer is highly heterogeneous at the molecular level it is likely that a panel of biomarkers will be required to detect all tumours. Theoretically, highly specific markers can be combined to generate an effective test. However, total levels of protein markers may be influenced by non-malignant conditions and haematuria, limiting specificity and therefore suitability for inclusion in multimarker tests. A test based on bladder cancer-specific variants of proteins would be expected to outperform a test based on the total levels of these proteins.

Numerous proteomic approaches have been applied to analyse urine in the search for bladder cancer biomarkers (reviewed in [10]). The urinary proteome is challenging to mine in depth, in part due to an abundance of plasma proteins, and many proteomic studies have suggested plasma proteins as biomarkers despite the fact that they are unlikely to be specific for bladder cancer. For example, Chen *et al.* carried out quantitative shotgun proteomics on pooled bladder cancer urine samples and non-cancer controls using iTRAQ for relative quantitation, followed by MRM quantitation of candidate biomarkers producing a multimarker panel with a ROC AUC of 0.814 [11,12]. The “biomarkers”, however, are moderately abundant plasma proteins rather than cancer-specific proteins. Top-down approaches have the ability to detect proteoforms not readily distinguishable in bottom-up approaches. However, to date, CE-MS and MALDI based profiling have failed to generate a highly sensitive and specific test for bladder cancer [13,14,15]. A small number of studies have used lectin affinity chromatography in studies of the urinary glycoproteome, however these studies have used broad specificity lectins (expected to capture most glycoproteins) rather than focussing on alterations in glycosylation [16,17]. Kreunin *et al.* [16] compared the glycoproteome of urine from bladder cancer patients and non-cancer patients using Concanavalin A (ConA) affinity chromatography combined with nano-liquid chromatography-tandem mass spectrometry (LC-MS/MS). Alpha-1β-glycoprotein was identified as the most discriminatory protein, but again, this is a plasma protein.

It has been reported that protein glycosylation is significantly altered in many cancers including bladder cancer [18]. Alterations in glycosylation patterns in cancer reflect changes in expression of glycosyltransferases and glycosidases [19]. The changes to glycan structure that can occur in cancer include *O*-glycan truncation, increased branching of *N*-glycans, and increased sialylation, sulfation and fucosylation [18]. There have been several studies on the glycosylation state of specific proteins found in the sera of cancer patients. For example, increased fucosylation and sialylation of PSA have been reported in prostate cancer [20]. Wu and colleagues used a fucose specific lectin, Aleuria aurantia lectin (AAL) to characterise the fucosylation of haptoglobin in ovarian cancer and found increased levels of fucosylated haptoglobin in patient sera [21]. Another study observed glycan-specific changes in periostin and thrombospondin in ovarian cancer [22]. All of these studies found that the specific protein glycoforms were able to better differentiate cancer sera from control serum samples than the total concentration of the protein. Perhaps the best example of a cancer specific glycoform is AFP-L3. An increase in total AFP concentration in the serum was originally used as an indicator for hepatocellular carcinoma (HCC); however, measuring the total AFP concentration cannot always discriminate between small HCCs and chronic liver disease. Further study of the protein identified a core-fucosylated form of AFP known as AFP-L3 which is specific to HCC and which can be measured in the serum to distinguish between HCC and chronic liver disease, making it a clinically useful biomarker [23]. Identifying a similarly cancer-specific glycoprotein biomarker for bladder cancer could be the answer to finding an accurate non-invasive test for disease detection and long-term surveillance of patients.

In the experiments reported here we test the hypothesis that incorporating selective lectin chromatography into urinary proteomics workflows has the potential to uncover urinary biomarkers for bladder cancer. We focus on low grade non-invasive bladder cancer as this is the form of the disease which is most challenging to detect using currently available non-invasive tests.

## 2. Materials and Methods

### 2.1. Materials and Cell Lines

All lectins were purchased from Vector Laboratories Ltd. (Peterborough, UK). All other chemicals and materials were purchased from Sigma-Aldrich (St. Louis, MI, USA) unless otherwise stated.

The bladder cancer cell lines 5637 and HB-CLS-2 were purchased from CLS Cell Lines Service GmbH (Eppelheim, Germany). NHU-TERT, VM-CUB-1, MGH-U3, RT4, RT112, SW780 and T24 were provided by Professor Margaret Knowles (University of Leeds, Leeds, UK). The UROtsa cell line was a gift from Alexander Dowell (University of Birmingham, Birmingham, UK). Cell lines were cultured as previously described [24]. The bladder cancer cell lines were derived from tumours of different grades as detailed in Supplemental Table S1. Cell lysates were prepared by sonication in PBS containing cOmplete EDTA-free protease inhibitor cocktail followed by a 10 min centrifugation at 13,000 rpm. The protein concentration of the supernatant was determined by Bradford assay.

### 2.2. Patient Samples

Patient urine and serum samples were collected as part of the Bladder Cancer Prognosis Programme (BCPP) [25]. Recruitment to BCPP was undertaken between 2005 and 2011 and consists of samples from patients with suspected primary bladder cancer. Midstream urine was collected, centrifuged at 2000 rpm for 10 min and the supernatant stored in aliquots at −80°C. After sample collection, each patient underwent TURBT and definitive diagnosis by histopathological examination of the resected tissue. Urine samples obtained from patients with non-malignant conditions were retained in the BCPP collection and have been used as non-cancer controls. For the lectin ELISA experiments, urine samples were pooled into four control pools (two normal and two from patients with cystitis or inflammation) and six cancer pools (two from each of stages pTa, pT1 and pT2+). For the proteomics experiments, two pools of pTa patient urine were used (*n* = 75; *n* = 36) and a non-cancer control urine pool (*n* = 28). All samples used were negative for haematuria by dipstick test. Further patient information is provided in Supplemental Materials.

### 2.3. Lectin ELISAs

Urine samples were diluted ×50 in PBS and cell lysates and serum samples were diluted to 3 ug protein/mL in PBS and 100 µL added to 96 well Maxisorb Immuno-plates followed by a 1 hour incubation at 37°C to adsorb proteins. The wells were washed three times with 200 μL PBS containing 0.05% *w*/*v* Tween^®^ 20 (PBST, Sigma-Aldrich). The plates were blocked with 1% BSA in PBS for one hour. After washing with PBST, 100 μL of biotinylated lectin at 10 μg/mL in PBS was added to the wells and incubated for 30 min. After washing with PBST, 100 μL of a 1 in 200 dilution of streptavidin conjugated to horseradish peroxidase (R&D Systems, Abingdon, UK) in 1% BSA in PBS was added and incubated for 30 min. The plates were then washed five times with PBST and 100 μL of substrate solution (3,3′,5,5′-tetramethlbenzidine) was added. The reaction was stopped with 40 μL of 2M H_2_SO_4_ and the absorbance measured at 450 nm. All ELISAs were performed in triplicate and means compared across experimental groups using *t*-tests.

### 2.4. Lectin Dot Blots

Samples were diluted (cell lysate or serum diluted to 3 ug protein/mL in PBS and urine diluted ×50 in PBS and 2 μL spotted onto nitrocellulose membrane. Once dry, the membrane was blocked with 1% BSA in PBS for 40 min and then washed three times with PBST. The membrane was incubated in a lectin solution at 10 ug/mL in PBS for 30 min, washed with PBST and incubated with streptavidin-HRP (as above). The membrane was washed with PBST and imaged using ECL and photographic film.

### 2.5. Lectin Affinity Chromatography

Lectin conjugated agarose beads were washed 10 times with PBS to remove sugars in their storage solution and 500 µL of 50% slurry mixed with 5 mL of pooled urine and 500 μL of 12 × PBS. Binding was allowed to occur during a 2 h incubation on a rotating mixer at 4°C. The beads were captured on filters and the flow through was collected and stored. The beads were then washed thoroughly with PBS and bound glycoproteins eluted with 2 × 400 μL of 100 mM l-fucose or 200 mM N-aceytl-d-galactosamine (GalNAc). In experiments using UEA1 and DBA (which require divalent cations) PBS was substituted with 150 mM NaCl, 1 mM CaCl_2_, 1 mM MgCl_2_, 100 uM MnCl_2_, 100 uM ZnSO_4_ and 20 mM MOPS, pH 7.4. All lectin affinity-chromatography–shotgun-proteomics experiments were performed as 2 independent replicates. 

### 2.6. Filter-Aided Sample Preparation and Tryptic Digestion

Up to 200 µg of protein was dissolved in 9 M Urea, 1% CHAPS (Melford Laboratories, Ipswich, UK) in 100 mM triethylammonium bicarbonate (TEAB), and incubated with 20 mM dithiothreitol (DTT) for 30 min at room temp. Following addition of 50 mM iodoacetamide the proteins were captured in 0.5 mL 30 kDa MWCO centrifugal filters, centrifuged at 13,000 rpm for five minutes and washed four times with 100 mM TEAB. Proteins were digested by incubating overnight at 37 °C with 5 μg of sequencing grade trypsin (Promega). Peptides were collected by centrifugation.

### 2.7. Stable Isotope Labelling

After tryptic digestion, formaldehyde was added to the control urine peptides to a final concentration of 0.2% *w*/*v* and deuterated formaldehyde was added to the pTa patient urine peptides. Sodium cyanoborohydride was added to both samples to a final concentration of 25 mM. After 30 min, 0.5 M ammonium bicarbonate was added to quench the reaction. The control and cancer urine samples were then combined and acidified with 10% trifluoroacetic acid (TFA), the peptides were captured on a C18 cartridge, washed with 0.1% TFA and peptides eluted with 600 μL of 60% acetonitrile (ACN)/0.1% TFA.

### 2.8. Peptide Fractionation and LC-MS/MS

Peptides were dried and dissolved in mixed mode buffer A (110 μL 20 mM ammonium formate, pH 6.5, 3% ACN) and separated into 16 fractions using an Acclaim^®^ Mixed-Mode WAX-1, 3 um, 120 Å (2.1 × 150 mm) column (Dionex, Camberley, UK) at a flow rate of 100 μL/min. The elution gradient used was 0%–50% of buffer B (2 mM ammonium formate, pH 3.0, 80% ACN) for 45 min followed by 50%–100% buffer B over five minutes and then 100%–0% buffer B for the last 10 min. Fractions were dried and reconstituted in 0.1% formic acid (FA) in water. The peptides in each fraction were analysed by LC-MS/MS using a 60 min gradient of 0%–36% ACN in 0.1% FA at a flow rate of 350 nL/min with an Acclaim^®^ Pepmap C18, 3 μm, 100 Å column (25 cm × 75 μm) (Thermo Scientific, Loughborough, UK) attached to an Ultimate 3000 RS HPLC system coupled to an Impact Quadrupole-TOF mass spectrometer (Bruker Daltonics, Coventry, UK) working in data-dependent mode at 5 MS/MS per cycle.

### 2.9. Peptide Identification and Analysis

All MS/MS spectra were searched against a database containing Swissprot human sequences and randomized versions thereof using MASCOT (version 2.3 Matrix Science Ltd, London, UK). Search parameters were as follows: (i) species: *Homo sapiens*; (ii) enzyme: trypsin; (iii) ≤2 missed cleavages; (iv) 10 ppm precursor ion tolerance; (v) 0.02 Da fragment ion tolerance; (vi) fixed modifications: cysteine carbamidomethylation; (vii) variable modifications: methionine oxidation; (viii) a peptide score of >25. For quantitative experiments light and heavy dimethylation of N-termini and lysine residues were also included as variable modifications. Proteinscape 3 software (Bruker Daltonics) was used to combine multiple search results and filter the data using a protein false discovery rate of 1%. WARP-LC (Bruker Daltonics) was used for relative quantitation based on extracted ion chromatograms and limma used for statistical analysis of differential expression [26].

## 3. Results

### 3.1. Lectin Binding to Urothelial Cell Line Lysates

**Figure 1:**
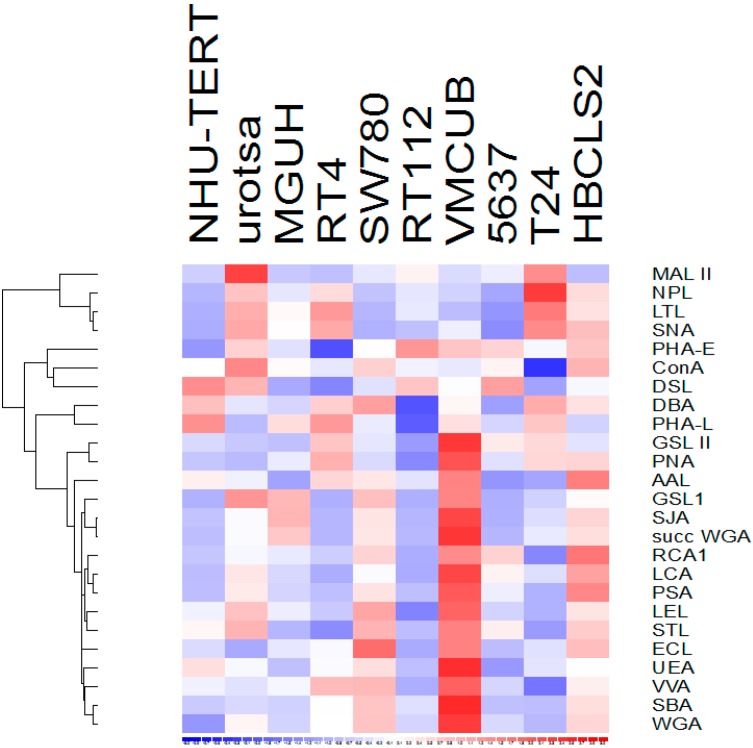
Lectin binding to urothelial cell lysates. Cell lines are shown from left to right, non-cancer (NHU-TERT & UROtsa), grade 1 (MGH-U3, RT4, SW780), grade 2 (RT112, VM-CUB-1, 5637) and grade 3 bladder cancer (T24, HB-CLS-2). The relative binding level of each lectin to the cell lines is shown on a sliding scale from high (red) to low (blue).

**Figure 2:**
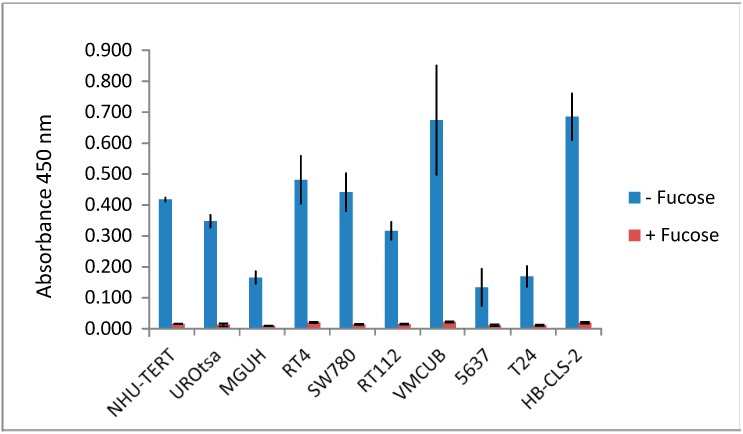
Fucose inhibition of AAL binding to cell lysates. Lectin ELISA results are shown for AAL binding (absorbance 450 nm) to cell lysates ±100 mM l-fucose.

Lectin ELISAs were used to evaluate the ability of 25 different lectins to bind to cell lysates of 2 normal urothelial human cell lines and 8 human bladder cancer cell lines derived from tumours of different grades (potentially allowing us to determine how glycosylation differs between low- and high-grade disease). Although different cell lines show quite different lectin binding profiles, none of the lectins showed consistent, substantially different binding between the normal and cancer cell lines or between cancer cell lines derived from low or high grade tumours (*p* > 0.05 in all cases). The data are summarised in Figure 1. Figure 2 demonstrates the specificity of AAL for fucose containing substrates: including 100 mM l-Fucose during the incubation of AAL on urothelial cell line lysate coated plates effectively prevents any binding from taking place.

### 3.2. Lectin Binding Properties of Urinary Proteins

**Figure 3:**
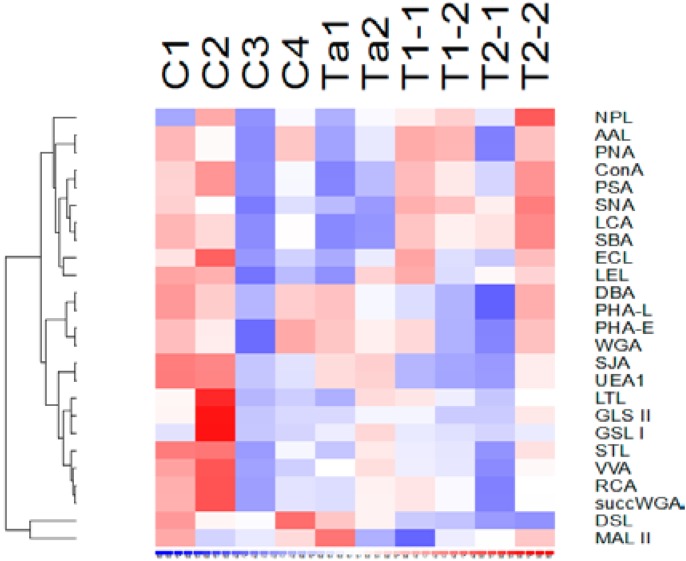
Lectin binding to pooled urine samples. Each pooled urine samples consists of urine from >8 patients with: C1&C2 = no abnormality detected, C3&C4 = no malignant disease (cystitis/inflammation), Ta1 & Ta2 = pTa UBC, T1-1 &T1-2 = pT1 UBC, T2-1 & T2-2 = MIBC. The relative binding level of each lectin to the 8 pooled urines is shown on a sliding scale from high (red) to low (blue).

The binding of the same panel of 25 lectins to the proteins in pooled urine from patients without bladder cancer or with pTa, pT1 or pT2+ bladder cancer was tested by ELISA. None of the lectins demonstrated a statistically significant higher or lower binding (*p* > 0.05) to the proteins in the urine of cancer patients relative to urine of non-cancer controls (Figure 3).

### 3.3. Lectins with Selectivity for Urothelial Glycans Relative to Plasma Glycans

**Figure 4:**
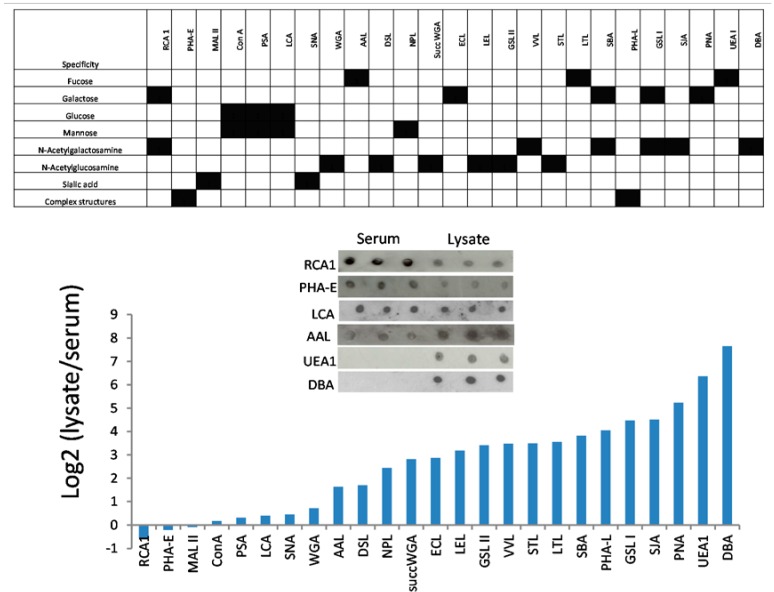
Lectin binding to urothelial cell line lysates and serum. The histogram shows data from lectin ELISAs run in triplicate (pooled cell lysate and pooled serum). The inserted panel shows confirmatory dot blots. With the exception of PHA-E, SNA and LCA all lectins showed significantly different binding to lysates and serum (*p* < 0.05).

A lectin that could enrich urothelial glycoproteins relative to plasma glycoproteins in urine would be a useful tool in urine proteomics. We therefore compared the binding of the 25 lectins to bladder cell line lysates with their binding to serum from non-cancer control subjects by lectin ELISA and confirmed selected results with lectin dot blots (Figure 4). Whilst RCA1 and PHA-E bound more strongly to serum than to cell lysate, the majority of lectins preferred the lysate with 10 lectins showing very low binding to serum. These lectins, in particular UEA1 and DBA, showed very high cell:serum binding ratios. Lectins that bind mannose, glucose or sialic acid tended to show low binding to urothelial glycans whereas fucose, galactose and *N*-acetylgalactosamine binding lectins exhibited high binding to urothelial glycans relative to serum.

### 3.4. Glycoproteome Analysis of Pooled pTa UBC Patient Urine

**Figure 5:**
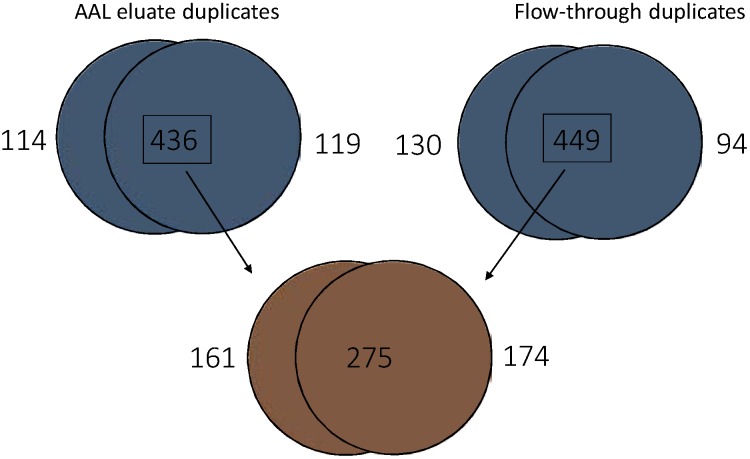
The number of proteins identified by LC-MS/MS in the AAL flow-through and eluted fractions. The upper Venn diagrams show the number of proteins identified in the flow-through and eluted fractions of two independent AAL chromatographies of a pooled urine sample from patients with pTa bladder cancer. The lower Venn diagram shows the overlap between the proteins identified in both eluates and both flow-throughs.

Shotgun proteomics was used to assess the ability of AAL, UEA and DBA affinity chromatography to extract subproteomes from a pooled bladder cancer urine sample (75 patients with G1-G3 pTa disease). UEA1 and DBA were chosen for glycoprotein enrichment due to their striking preference for urothelial proteins over serum proteins and AAL was chosen because it not only displayed a preference for urothelial proteins over serum proteins, but has previously been shown to have an affinity for cancer-related glycoproteins. LC-MS/MS analysis of the proteins bound to UEA1 or DBA and eluted with 100 mM l-fucose or 200 mM *N-*aceytl-d-galactosamine respectively identified surprisingly few proteins: DBA captured 140 proteins of which 75 were found in two experimental replicates and UEA1 captured 122 proteins of which 69 were common to both replicates. Furthermore, these proteins included keratins and abundant proteins such as uromodulin and albumin indicative of non-specific binding. In contrast, in both experimental replicates, more than 500 proteins were eluted from AAL with 100 mM l-Fucose. There was a high degree of overlap between the proteins identified in the AAL eluates in both replicates with 436 protein identifications common to both (Figure 5). Of these 436 proteins, 285 contain glycosylation sites (65%). Of these 285 glycoproteins, 274 possess *N*-linked glycans and 24 possess *O*-linked glycans.

The AAL flow throughs (*i.e*., proteins not captured by AAL) were also analysed by LC-MS/MS and the protein identifications compared with the proteins identified in the AAL eluate to determine which proteins were enriched by the AAL affinity chromatography. We defined proteins as enriched by AAL if they were present in both eluates and were identified by at least twice as many peptides in the eluates as in the flow throughs. Using these criteria, AAL enriched 186 proteins. Of these 186 enriched proteins, 115 (62%) contain glycosylation sites and 84 of these proteins were not identified in the flow-throughs. Of the 186 enriched proteins, 70 are associated with the extracellular space and 71 with the plasma membrane. This suggests that AAL lectin affinity chromatography may be able to identify proteins originating from the urothelial cell surface or proteins released from the urothelium. The levels of abundant plasma proteins were decreased in the AAL eluates relative to the flow-thoughs: albumin decreased from 8307 peptide spectrum matches in the flow-throughs to 585 in the eluates and serotransferrin decreased from 950 to 76 peptide spectrum matches. Information on the protein identifications are provided in Supplemental Materials.

### 3.5. Quantitative Comparison of AAL Binding Proteins in the Urine of Control Subject and Patients with G1 pTa Bladder Cancer

**Figure 6:**
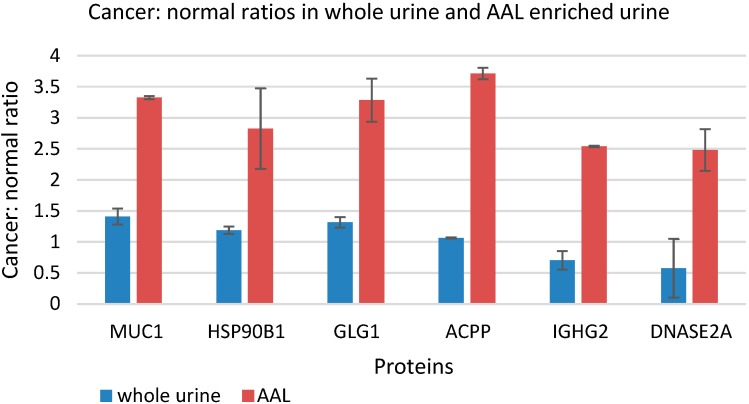
AAL binding proteins and their cancer: normal peptide intensity ratios for the whole urine experiment and the AAL experiment. Mucin-1 (MUC1), endoplasmin (HSP90B1), golgi apparatus protein 1 (GLG1), prostastic acid phosphatase (ACPP), Ig gamma-2 chain C region (IGHG2), and deoxyribonuclease-2-alpha (DNASE2A) all have a greater cancer: normal ratio after AAL enrichment.

AAL affinity chromatography was performed on pooled urine samples from patients with G1 pTa bladder cancer (*n* = 36) and non-cancer controls (*n* = 28). The eluted proteins were digested and the peptides stable isotope labelled as described in the method section, the samples combined and analysed by shotgun proteomics. Duplicate experiments again proved reproducible with 394 protein identified in both AAL eluates. We also analysed the 2 pooled urine samples without AAL enrichment in duplicate with 501 protein identifications common to replicates. The heavy/light peptide intensity ratios were used to estimate the relative levels of proteins in the two pooled urine samples in the AAL eluate and whole urine datasets. In the AAL eluates the concentrations of 21 proteins were significantly (*p* << 0.01) and substantially (≥2-fold increase in both experimental replicates) higher in the pooled pTa urine than the control urine. Of these, 12 proteins were also increased in the cancer sample in the whole urine experiments whereas 9 of the proteins were increased in cancer only in the AAL eluates. The 12 proteins elevated in cancer in both the whole urine and AAL eluates are likely to be present at a higher total concentration in the cancer sample whereas the 9 proteins increased in the AAL eluates but not whole urine could be cancer specific glycoforms. Of the 9 proteins with apparent altered glycosylation (rather than simply an increase in total concentration), 6 were previously identified as released by bladder cancer cell lines *in vitro* [24] (mucin-1 (MUC1), golgi apparatus protein 1 (GLG1), endoplasmin (HSP90B1), prostatic acid phosphatase (ACPP), Ig gamma-2 chain C region (IGHG2), and deoxyribonuclease-2-alpha (DNASE2A)), and 3 were not (voltage-dependent anion-selective channel 1, carbonic anhydrase 1 and bile salt-activated lipase 11) . The cancer:normal intensity ratio in the AAL eluate and the whole urine data for the proteins previously identified as released by bladder cancer cell lines is shown in Figure 6.

## 4. Discussion

Current urinary protein biomarkers for bladder cancer lack the sensitivity and/or specificity required to have utility in the clinic. Aberrant glycosylation has been widely reported in cancer and some cancer biomarkers utilise changes in protein glycosylation to improve biomarker effectiveness. In this study we investigated whether cancer specific glycoforms are a feature of bladder cancer and whether lectin affinity chromatography is a useful tool in urine proteomics. In summary, the binding of 25 different lectins to bladder cancer cell lines or urine from control subjects and patients with bladder cancer did not detect any major global changes in glycosylation. We did, however, find that many lectins have a preference for bladder cancer cell line proteins over plasma proteins and this may be useful for discriminating between proteins that are released into the urine from the urothelium and those that are filtered through the kidney or leak in due to haematuria.

A recent paper by Yang *et al.* reported that the binding of many lectins differed significantly between a normal urothelial cell line (HCV29) and several bladder cancer cell lines, and went on to show increased binding of LCA and SNA and decreased binding of ConA to bladder cancer tissue relative to adjacent normal tissue [27]. The use of different reference cell lines (NHU-TERT and UROtsa) in our study may be the reason that we did not see clear differences between the non-cancer and cancer cell lines, although differences between individual cell lines were observed. The lack of evidence for cancer-specific changes in glycosylation in our experiments does not exclude the possibility that they exist since we looked globally at the whole glycoproteome rather than determining the glycosylation status of individual proteins. The fact that no lectins demonstrated preferential binding to bladder cancer patient urine samples over control urine samples may reflect that the vast majority of the proteins in the urine samples are not tumour derived. Consistent with this finding, a recent study of urinary glycans found only small changes in the *N*- and *O*-linked glycomes of patients with bladder cancer [28]. The literature on lectin binding to bladder cancer tissues is complex with no clear consensus as to which lectins bind preferentially to tumours over normal tissue or relationships of lectin binding to stage, grade and outcome [29,30]. These data indicate that specific glycoprotein markers will be required rather than global changes in glycosylation.

Perhaps our most important finding is that several lectins are selective for urothelial proteins over plasma proteins. This is a useful characteristic for bladder cancer urine proteomics because low abundance urothelial proteins can be masked from detection by mass spectrometry by the presence of highly abundant serum proteins in the urine. The broad specificity lectins WGA and ConA that have been used in previous urinary glycoprotoemic studies [16,17] however, are not selective for urothelial proteins over plasma proteins. When we tested the ability of the 2 lectins with the greatest selectivity for urothelial proteins, UEA1 and DBA, to enrich urothelial glycoproteins from urine we identified only a small number of proteins. Perhaps these unexpected results are because the lectins recognise cellular proteins that are not released into the urine or because the eluting sugars were unable to effectively compete with the glycoprotein-lectin interaction. Nonetheless, it would seem that the other lectins with a high lysate/serum ratio (PNA, SJA, GSL I, SBA, PHA-L, STL, VVA) should be considered in urinary glycoproteomic workflows. AAL was selected in this study partly because it showed a small preference for urothelial proteins but more so because it has previously been used to enrich aberrantly glycosylated proteins in HCC [31]. AAL is specific for core and terminal fucose structures including fucose (α-1,6) linked to *N*-acetylglucosamine and fucose (α-1,3) linked to structures related to *N*-acetyllactosamine (85). The fucose (α-1,6) residue is a core fucose structure that is present in many mammalian tissues and has been reported to be altered in pathological settings.

Of the 580 unique proteins captured by AAL, 6 behave as if they are aberrantly glycosylated in bladder cancer: mucin-1, golgi apparatus protein 1, prostatic acid phosphatase, endoplasmin, deoxyribonuclease-2-alpha and Ig gamma-2 chain C region. The selection was based on the fact that they are all released by bladder cell lines [24] and it appears that it is their glycosylation status rather than the total quantity of these proteins which change in bladder cancer. For two of these proteins, Deoxyribonuclease-2-alpha (DNASE2) and Ig gamma-2 chain C region (IGHG2) there is a lack of further evidence for a role in bladder. Endoplasmin (HSP90B1) is a ubiquitously expressed molecular chaperone of plasma membrane associated and secreted proteins [32]. Heat shock proteins are widely reported as overexpressed in cancer and one study reported that HSP90B1 is overexpressed in canine bladder cancer [33]. Prostatic acid phosphatase (ACPP) is a 100 kDa tyrosine phosphatase that dephosphorylates a diverse array of substrates under acidic conditions [34]. ACPP exists as intracellular and secreted forms that possess different glycosylation patterns and different hydrophobicities [35]. Although serum ACPP can be used to monitor prostate cancer it is reportedly not expressed in bladder cancer [36] and is not likely be a good biomarker for bladder cancer (because urinary ACPP may be primarily derived from the prostate) unless a genuinely bladder cancer specific glycoform exists. MUC1 and GLG1 appear to be the most interesting of the 6 candidates as discussed below.

MUC1 is a transmembrane protein present in normal urothelium on the apical surfaces of umbrella cells and acts to protect the cells from adhesion of bacteria [37]. Overexpression and changes in glycosylation of MUC1 have been reported in lung, breast, ovary, colon and bladder cancer [38]. In an immunohistochemistry study of 539 bladder tumours MUC1 was expressed in 62% of the tumours and increased with tumour grade [39]. Serum levels of MUC1 are elevated in patients with late stage bladder cancer but sensitivity for early disease is poor [38]. An investigation of urinary levels of MUC1 31 patients with TCC and 30 control patients found no significant difference between patient groups [40]. However, total MUC1 was measured whereas our data suggest that it is an alternatively glycosylated form of MUC1 that is increased in the urine of bladder cancer patients.

The golgi apparatus protein 1 (GLG1), also known as CFR, ESL-1 and MG-160, is a 135 kDa glycosylated single pass type I transmembrane protein that contains 16 cysteine-rich GLG1 repeats [41]. It is found in the golgi apparatus and on the cell surface membrane. GLG1 is able to bind with many different proteins making it an important regulatory protein and signal transducer. The localisation of GLG1 is a crucial determinant of GLG1 function and is influenced by two mechanisms: the transmembrane domain and cytoplasmic tail retain GLG1 in the golgi apparatus, whereas the cys-rich repeats destabilise the protein and GLG1 is recruited to the cell surface via processes of stability control [41]. It has also been reported that GLG1 can be released from the cell by proteolytic cleavage at the juxtamembrane region [42]. These mechanisms of localisation and proteolytic cleavage may be altered in cancer and may play a role in the increased presence of GLG1 in the urine. GLG1 has been shown by immunohistochemistry to be highly or intermediately expressed in both high and low grade urothelial cancer tissue samples [43]; furthermore, GLG1 was detected on the cell surface of bladder cancer cell lines (Ward, unpublished data). Therefore, it seems plausible that the GLG1 detected in pTa patient urine is tumour derived.

## 5. Conclusions

Our data demonstrate a role for certain lectins in urinary biomarker discovery. The urinary glycoproteome has not been fully explored to date and using the lectins with a strong preference for urothelial proteins over serum proteins (Figure 4) in conjunction with shotgun proteomics may identify much needed biomarkers for bladder cancer. To assess the biomarker potential of the candidate glycoprotein biomarkers suggested by the current work, initial validation could be carried out by lectin affinity chromatography combined with an ELISA using specific antibodies against the target glycoprotein. If the glycoprotein concentration in urine samples from bladder cancer patients is confirmed as significantly greater than the concentration in control samples then a more streamlined assay such as a sandwich ELISA combining lectin and antibody binding would be required for full validation and ultimately clinical use.
