# Supplementary material for: Use of Aleuria alantia Lectin Affinity Chromatography to Enrich Candidate Biomarkers from the Urine of Patients with Bladder Cancer
Source: Proteomes. 2015 Sep 3;3(3):266–82. doi: 10.3390/proteomes3030266 (PMC5217382; doi:10.3390/proteomes3030266)
Supplement: Supplementary file 1 [file proteomes-03-00266-s001.pdf]

Supplementary Materials

Table S1. Urothelial cell lines. 5637 and HB-CLS-2 were purchased from CLS Cell Lines Service GmbH (Eppelheim, Germany). VM-CUB-1, MGH-U3, RT4, RT112, SW780 and T24 were validated and selected on the basis that they exhibit a diversity of mutation profiles and therefore may capture some of the heterogeneity of UBC. The UROtsa immortalised normal urothelium cell line was a gift from Alexander Dowell.

Cell Line	Grade of Tumour	Mutations	Growth Conditions
NHU-TERT	N/A	ND	SF keratinocyte medium
UROtsa	N/A	ND	RPMI −1640 + 10% FCS
MGH-U3	1	TP53: WT<br>FGFR3: Y375C<br>RAS: WT<br>PIK3CA: WT<br>TSC1: WT<br>AKT1: E17K	DMEM + 10% FCS + 2 mM L-glutamine +1% non-essential amino acids
RT4	1	TP53: WT<br>FGFR3: translocation<br>RAS: WT<br>PIK3CA: WT<br>TSC1:c.1669delC<br>AKT1: WT	McCoy's 5A + 10% FCS + 2 mM L-glutamine
SW-780	1	TP53: WT<br>FGFR3: translocation<br>RAS: WT<br>PIK3CA: WT<br>TSC1: WT<br>AKT1: WT	DMEM +10% FCS + 2 mM L-glutamine
RT112	2	TP53: WT<br>FGFR3: translocation<br>RAS: WT<br>PIK3CA: WT<br>TSC1: WT<br>AKT1: WT	RPMI-1640 + 10% FCS
VM-CUB-1	2	TP53: R175H<br>FGFR3: WT<br>RAS: WT<br>PIK3CA: E545K<br>TSC1: WT<br>AKT1: WT	DMEM + 10% FCS + 2 mM L-glutamine
5637	2	TP53: R280T<br>RAS: WT<br>PIK3CA: WT<br>TSC1: WT<br>AKT1: WT	RPMI-1640 + 10% FCS

Table S1. *Cont.*

Cell Line	Grade of Tumour	Mutations	Growth Conditions
T-24	3	TP53: Y126*<br>FGFR3:WT<br>RAS: HRAS p.G12V<br>PIK3CA: WT<br>TSC1: WT<br>AKT1: WT	DMEM + 10% FCS + 2 mM L-glutamine
HB-CLS-2	3	ND	RPMI-1640 + 10% FCS

Table S2. Proteins identified in the AAL eluate from pTa urine in both experimental replicates. Proteins are sorted according to their average score.

Accession	Protein Name	AAL1 #Peptides	AAL1 Score	AAL2 #Peptides	AAL2 Score
LRP2_HUMAN	Low-density lipoprotein receptor-related protein 2	**94**	6243	**96**	6360.7
CUBN_HUMAN	Cubilin	**76**	5130.5	**76**	5196.6
CO3_HUMAN	Complement C3	**72**	4239.1	**73**	4604
FCGBP_HUMAN	IgGFc-binding protein	**47**	3049.8	**56**	3631.9
A2MG_HUMAN	Alpha-2-macroglobulin	**45**	2500.9	**45**	2790.2
ALBU_HUMAN	Serum albumin	**44**	2965.8	**34**	1933.5
CERU_HUMAN	Ceruloplasmin	**33**	2339.3	**36**	2497.7
K1C10_HUMAN	Keratin, type I cytoskeletal 10	**37**	2342.4	**31**	2087.9
K2C1_HUMAN	Keratin, type II cytoskeletal 1	**38**	2436.9	**28**	1755.2
APOB_HUMAN	Apolipoprotein B-100	**33**	1534.5	**43**	2360.3
K1C9_HUMAN	Keratin, type I cytoskeletal 9	**29**	2027.4	**23**	1643.9
UROM_HUMAN	Uromodulin	**28**	1667.1	**33**	1914.4
K22E_HUMAN	Keratin, type II cytoskeletal 2 epidermal	**34**	2057.1	**23**	1509.8
PIGR_HUMAN	Polymeric immunoglobulin receptor	**23**	1569.6	**22**	1697.8
EGF_HUMAN	Pro-epidermal growth factor	**22**	1549.9	**24**	1630.7
AMPN_HUMAN	Aminopeptidase N	**27**	1551.5	**24**	1465.2
FIBB_HUMAN	Fibrinogen beta chain	**19**	1393.5	**23**	1582.6
A1AT_HUMAN	Alpha-1-antitrypsin	**21**	1502.9	**18**	1396.6
MGA_HUMAN	Maltase-glucoamylase, intestinal	**23**	1332.1	**24**	1399.4
ACTB_HUMAN	Actin, cytoplasmic 1	**15**	1077	**20**	1445.1
IGKC_HUMAN	Ig kappa chain C region	**10**	1237.8	**11**	1173.7
TRFE_HUMAN	Serotransferrin	**19**	1220.4	**20**	1165.7
FINC_HUMAN	Fibronectin	**18**	1135.3	**23**	1231.5
FIBG_HUMAN	Fibrinogen gamma chain	**17**	937	**24**	1355.1
ATRN_HUMAN	Attractin	**13**	1029	**18**	1200.4
AMBP_HUMAN	Protein AMBP	**19**	1271.6	**16**	929.8
K1C14_HUMAN	Keratin, type I cytoskeletal 14	**17**	1154	**13**	1017.5

Table S2. *Cont.*

Accession	Protein Name	AAL1 #Peptides	AAL1 Score	AAL2 #Peptides	AAL2 Score
DPP4_HUMAN	Dipeptidyl peptidase 4	**18**	1036.7	**19**	1026.2
LG3BP_HUMAN	Galectin-3-binding protein	**11**	949.8	**14**	1110.9
K1H1_HUMAN	Keratin, type I cuticular Ha1	**25**	1422.4	**9**	542.5
IGHA1_HUMAN	Ig alpha-1 chain C region	**12**	949.7	**13**	998.2
IGHA2_HUMAN	Ig alpha-2 chain C region	**12**	949.9	**12**	970.5
IPSP_HUMAN	Plasma serine protease inhibitor	**15**	973.3	**18**	942.5
KRT83_HUMAN	Keratin, type II cuticular Hb3	**29**	1466.7	**8**	390.7
FIBA_HUMAN	Fibrinogen alpha chain	**11**	695	**18**	1119.4
KRT86_HUMAN	Keratin, type II cuticular Hb6	**28**	1385.2	**8**	413.3
KT33A_HUMAN	Keratin, type I cuticular Ha3-I	**23**	1326.3	**7**	449.3
CLUS_HUMAN	Clusterin	**14**	781.4	**16**	907.1
LYAG_HUMAN	Lysosomal alpha-glucosidase	**13**	725.3	**16**	918.4
SORL_HUMAN	Sortilin-related receptor	**14**	842.6	**17**	794.7
IC1_HUMAN	Plasma protease C1 inhibitor	**13**	758.1	**13**	846.1
IGHG1_HUMAN	Ig gamma-1 chain C region	**11**	734.5	**13**	865.2
THBG_HUMAN	Thyroxine-binding globulin	**12**	828.9	**11**	762.4
IGHM_HUMAN	Ig mu chain C region	**10**	601.1	**14**	966.4
THRB_HUMAN	Prothrombin	**12**	692.4	**13**	829.6
HBB_HUMAN	Hemoglobin subunit beta	**9**	593.9	**14**	919.1
AMPE_HUMAN	Glutamyl aminopeptidase	**15**	809.3	**14**	694.8
APOA1_HUMAN	Apolipoprotein A-I	**15**	724.6	**16**	763.9
HPT_HUMAN	Haptoglobin	**11**	699.5	**12**	734.8
ANAG_HUMAN	Alpha-N-acetylglucosaminidase	**14**	766.4	**10**	667.4
NEP_HUMAN	Neprilysin	**9**	616.2	**14**	803.8
ALDOB_HUMAN	Fructose-bisphosphate aldolase B	**9**	598.6	**12**	804.9
AACT_HUMAN	Alpha-1-antichymotrypsin	**12**	845	**9**	549.9
SUSD2_HUMAN	Sushi domain-containing protein 2	**8**	810.3	**8**	574
BTD_HUMAN	Biotinidase	**8**	630.9	**10**	711.7
IGHG3_HUMAN	Ig gamma-3 chain C region	**14**	697.7	**13**	639.6
PAPP2_HUMAN	Pappalysin-2	**12**	639.7	**11**	674.9
MMP9_HUMAN	Matrix metalloproteinase-9	**12**	599.3	**15**	711.4
CO9_HUMAN	Complement component C9	**9**	539	**14**	751.6
KNG1_HUMAN	Kininogen-1	**11**	675.8	**9**	550.1
CO6A1_HUMAN	Collagen alpha-1(VI) chain	**10**	719.6	**8**	484.9
APOD_HUMAN	Apolipoprotein D	**11**	638.6	**9**	544.6
G3P_HUMAN	Glyceraldehyde-3-phosphate dehydrogenase	**9**	490.1	**9**	670.1
CO5_HUMAN	Complement C5	**11**	593.2	**12**	566.1
OLFM4_HUMAN	Olfactomedin-4	**9**	549.2	**10**	607.2
ITIH1_HUMAN	Inter-alpha-trypsin inhibitor heavy chain H1	**9**	604	**10**	540.8

Table S2. *Cont.*

Accession	Protein Name	AAL1 #Peptides	AAL1 Score	AAL2 #Peptides	AAL2 Score
K2C6C_HUMAN	Keratin, type II cytoskeletal 6C	**15**	766.2	**8**	378.4
APOE_HUMAN	Apolipoprotein E	**9**	610.7	**8**	513
MYH9_HUMAN	Myosin-9	**12**	622.7	**10**	490.6
HBD_HUMAN	Hemoglobin subunit delta	**8**	454.9	**10**	656.4
FBLN3_HUMAN	EGF-containing fibulin-like extracellular matrix protein 1	**7**	514.6	**8**	594.8
GGT1_HUMAN	Gamma-glutamyltranspeptidase 1	**8**	538.1	**9**	565.2
PCD24_HUMAN	Protocadherin-24	**9**	510.3	**10**	588.6
AMYP_HUMAN	Pancreatic alpha-amylase	**8**	467.9	**9**	627.8
KRT35_HUMAN	Keratin, type I cuticular Ha5	**11**	661.8	**6**	427.2
GSLG1_HUMAN	Golgi apparatus protein 1	**9**	532	**10**	549.2
ITIH4_HUMAN	Inter-alpha-trypsin inhibitor heavy chain H4	**6**	373.9	**10**	640.6
K2C5_HUMAN	Keratin, type II cytoskeletal 5	**12**	585.8	**9**	425.5
TRFL_HUMAN	Lactotransferrin	**9**	553.4	**6**	442.6
IGHG4_HUMAN	Ig gamma-4 chain C region	**8**	469.8	**10**	514.3
KPYM_HUMAN	Pyruvate kinase isozymes M1/M2	**8**	503.5	**8**	444.4
PPAP_HUMAN	Prostatic acid phosphatase	**9**	536	**7**	402
FBLN1_HUMAN	Fibulin-1	**8**	510.2	**8**	421.2
ANT3_HUMAN	Antithrombin-III	**10**	528.1	**6**	396.4
K1C17_HUMAN	Keratin, type I cytoskeletal 17	**11**	530.5	**8**	383.4
ENOA_HUMAN	Alpha-enolase	**6**	398.6	**8**	511.5
CFAB_HUMAN	Complement factor B	**9**	418.6	**10**	489.5
PROZ_HUMAN	Vitamin K-dependent protein Z	**9**	433.5	**7**	462.3
IGHG2_HUMAN	Ig gamma-2 chain C region	**8**	392.1	**10**	501.9
MUC5B_HUMAN	Mucin-5B	**9**	422.6	**9**	429
PEDF_HUMAN	Pigment epithelium-derived factor	**6**	376.3	**7**	462
GELS_HUMAN	Gelsolin	**9**	501.2	**6**	317.7
CPN2_HUMAN	Carboxypeptidase N subunit 2	**4**	360.4	**6**	449.9
MMP8_HUMAN	Neutrophil collagenase	**7**	371.8	**6**	431.8
MEGF8_HUMAN	Multiple epidermal growth factor-like domains protein 8	**6**	348.5	**7**	434
LMAN2_HUMAN	Vesicular integral-membrane protein VIP36	**5**	304.5	**11**	471.6
BGAL_HUMAN	Beta-galactosidase	**4**	253.4	**8**	509.4
1433Z_HUMAN	14-3-3 protein zeta/delta	**3**	289.4	**7**	470.8
ROBO4_HUMAN	Roundabout homolog 4	**8**	417.7	**5**	340.5
PGK1_HUMAN	Phosphoglycerate kinase 1	**7**	402.6	**5**	347.6
K1H2_HUMAN	Keratin, type I cuticular Ha2	**8**	434.5	**4**	278.1
PPAL_HUMAN	Lysosomal acid phosphatase	**7**	329.1	**7**	379.8

Table S2. *Cont.*

Accession	Protein Name	AAL1 #Peptides	AAL1 Score	AAL2 #Peptides	AAL2 Score
HSP7C_HUMAN	Heat shock cognate 71 kDa protein	**6**	373.1	**6**	327.5
FREM2_HUMAN	FRAS1-related extracellular matrix protein 2	**7**	382.5	**7**	314.6
PSCA_HUMAN	Prostate stem cell antigen	**5**	366.8	**4**	326.7
HSP71_HUMAN	Heat shock 70 kDa protein 1A/1B	**5**	349.8	**6**	340.1
MASP2_HUMAN	Mannan-binding lectin serine protease 2	**5**	330	**5**	336.4
VASN_HUMAN	Vasorin	**6**	361.9	**5**	302.2
LDHB_HUMAN	L-lactate dehydrogenase B chain	**5**	323.9	**5**	338.7
APOA4_HUMAN	Apolipoprotein A-IV	**4**	218.2	**8**	440.6
ITIH2_HUMAN	Inter-alpha-trypsin inhibitor heavy chain H2	**4**	188.4	**9**	469.8
HPTR_HUMAN	Haptoglobin-related protein	**5**	268.8	**7**	386.9
HS90A_HUMAN	Heat shock protein HSP 90-alpha	**7**	296.8	**7**	353.8
DPP2_HUMAN	Dipeptidyl peptidase 2	**5**	316.4	**6**	327
CATD_HUMAN	Cathepsin D	**7**	319.8	**6**	305.4
QSOX1_HUMAN	Sulfhydryl oxidase 1	**7**	266.5	**7**	356.9
CO7_HUMAN	Complement component C7	**5**	277.6	**5**	342.3
AFAM_HUMAN	Afamin	**6**	320.4	**6**	293.9
LUM_HUMAN	Lumican	**9**	375.5	**5**	237.3
HEG1_HUMAN	Protein HEG homolog 1	**5**	330.5	**3**	280.4
CAH1_HUMAN	Carbonic anhydrase 1	**6**	296.9	**6**	300.1
HS90B_HUMAN	Heat shock protein HSP 90-beta	**5**	255.5	**7**	333.9
HBA_HUMAN	Hemoglobin subunit alpha	**5**	244.5	**6**	327
PGRP2_HUMAN	N-acetylmuramoyl-L-alanine amidase	**3**	211.4	**4**	359.8
CO8B_HUMAN	Complement component C8 beta chain	**6**	341.9	**5**	225.7
CD14_HUMAN	Monocyte differentiation antigen CD14	**3**	199.4	**6**	366
SODE_HUMAN	Extracellular superoxide dismutase [Cu-Zn]	**4**	265.4	**5**	287
ABHEB_HUMAN	Abhydrolase domain-containing protein 14B	**6**	288.8	**4**	255.6
CBG_HUMAN	Corticosteroid-binding globulin	**5**	242.1	**6**	297.5
PLSL_HUMAN	Plastin-2	**5**	232.9	**7**	296.7
PLMN_HUMAN	Plasminogen	**4**	189.2	**7**	333.6
AGAL_HUMAN	Alpha-galactosidase A	**3**	275.8	**3**	244
ACY1_HUMAN	Aminoacylase-1	**4**	177.7	**7**	340.7
CFAI_HUMAN	Complement factor I	**3**	229.5	**4**	287.4
CO2_HUMAN	Complement C2	**5**	214.2	**6**	299

Table S2. *Cont.*

Accession	Protein Name	AAL1 #Peptides	AAL1 Score	AAL2 #Peptides	AAL2 Score
TSP1_HUMAN	Thrombospondin-1	**3**	185.1	**5**	327.7
ANGT_HUMAN	Angiotensinogen	**4**	210.9	**5**	296.6
PTPRJ_HUMAN	Receptor-type tyrosine-protein phosphatase eta	**4**	235	**6**	270.9
COFA1_HUMAN	Collagen alpha-1(XV) chain	**7**	283.6	**4**	221.8
ARSA_HUMAN	Arylsulfatase A	**3**	175.7	**6**	324
ABP1_HUMAN	Amiloride-sensitive amine oxidase [copper-containing]	**3**	187.9	**6**	308.1
CHL1_HUMAN	Neural cell adhesion molecule L1-like protein	**4**	154	**7**	336.5
4F2_HUMAN	4F2 cell-surface antigen heavy chain	**6**	268.1	**4**	217.2
ALDOA_HUMAN	Fructose-bisphosphate aldolase A	**2**	101.3	**8**	383.9
BHMT1_HUMAN	Betaine--homocysteine S-methyltransferase 1	**3**	207.6	**5**	275
DPEP1_HUMAN	Dipeptidase 1	**4**	225.7	**4**	252.6
SPHM_HUMAN	N-sulphoglucosamine sulphohydrolase	**4**	208.9	**5**	269.2
HEP2_HUMAN	Heparin cofactor 2	**3**	230.9	**4**	243.8
ACTN4_HUMAN	Alpha-actinin-4	**5**	232.5	**4**	237
KV402_HUMAN	Ig kappa chain V-IV region Len	**3**	252.1	**3**	211.8
CADM4_HUMAN	Cell adhesion molecule 4	**5**	293.9	**3**	169.5
A2GL_HUMAN	Leucine-rich alpha-2-glycoprotein	**3**	192.1	**4**	270.2
CO8A_HUMAN	Complement component C8 alpha chain	**4**	238	**4**	222
KV305_HUMAN	Ig kappa chain V-III region WOL	**2**	210	**2**	248.1
IDHC_HUMAN	Isocitrate dehydrogenase [NADP] cytoplasmic	**4**	143	**6**	314.8
ACE_HUMAN	Angiotensin-converting enzyme	**4**	261.4	**3**	191.8
GSTM4_HUMAN	Glutathione S-transferase Mu 4	**3**	187.4	**4**	251.8
GSTM3_HUMAN	Glutathione S-transferase Mu 3	**3**	208.2	**3**	221.1
LRC19_HUMAN	Leucine-rich repeat-containing protein 19	**3**	192.9	**3**	233.6
PGRP1_HUMAN	Peptidoglycan recognition protein 1	**2**	144.1	**3**	282.2
PTGDS_HUMAN	Prostaglandin-H2 D-isomerase	**4**	244.5	**3**	181
FETUA_HUMAN	Alpha-2-HS-glycoprotein	**3**	156.5	**4**	264.6
ACE2_HUMAN	Angiotensin-converting enzyme 2	**2**	119	**6**	296.9
KRT82_HUMAN	Keratin, type II cuticular Hb2	**6**	262.4	**3**	145
6PGL_HUMAN	6-phosphogluconolactonase	**4**	166.7	**4**	235.6
PON1_HUMAN	Serum paraoxonase/arylesterase 1	**4**	213.6	**4**	185.1

Table S2. *Cont.*

Accession	Protein Name	AAL1 #Peptides	AAL1 Score	AAL2 #Peptides	AAL2 Score
HV303_HUMAN	Ig heavy chain V-III region VH26	**4**	186.5	**4**	207.2
LAC_HUMAN	Ig lambda chain C regions	**3**	180.8	**3**	212.1
NID1_HUMAN	Nidogen-1	**4**	153.6	**4**	233.6
KV105_HUMAN	Ig kappa chain V-I region DEE	**2**	217	**2**	168.9
CPVL_HUMAN	Probable serine carboxypeptidase CPVL	**6**	250.3	**3**	134.1
VWF_HUMAN	von Willebrand factor	**3**	115.6	**7**	268
CLM9_HUMAN	CMRF35-like molecule 9	**3**	187.4	**3**	192.5
A1BG_HUMAN	Alpha-1B-glycoprotein	**5**	217.3	**3**	162.3
VTNC_HUMAN	Vitronectin	**3**	164.6	**4**	214.5
HV307_HUMAN	Ig heavy chain V-III region CAM	**4**	176.5	**4**	202.5
HEMO_HUMAN	Hemopexin	**4**	205	**4**	169.7
FAT2_HUMAN	Protocadherin Fat 2	**3**	136.3	**5**	236.7
ZA2G_HUMAN	Zinc-alpha-2-glycoprotein	**3**	172	**4**	200.2
MA1A1_HUMAN	Mannosyl-oligosaccharide 1,2-alpha-mannosidase IA	**3**	155.9	**5**	215.1
FBLN4_HUMAN	EGF-containing fibulin-like extracellular matrix protein 2	**2**	119.8	**5**	250.2
STOM_HUMAN	Erythrocyte band 7 integral membrane protein	**3**	168.3	**3**	200.8
DMBT1_HUMAN	Deleted in malignant brain tumors 1 protein	**3**	208.2	**2**	157.7
TSN1_HUMAN	Tetraspanin-1	**2**	173.8	**2**	191.7
GAS6_HUMAN	Growth arrest-specific protein 6	**3**	139.8	**3**	224.9
TGM4_HUMAN	Protein-glutamine gamma-glutamyltransferase 4	**4**	208.8	**3**	155.5
ZG16B_HUMAN	Zymogen granule protein 16 homolog B	**2**	188.6	**2**	172.6
PVR_HUMAN	Poliovirus receptor	**3**	137.6	**3**	217.7
SPB3_HUMAN	Serpin B3	**3**	174.8	**4**	176.3
CFAH_HUMAN	Complement factor H	**3**	126.2	**5**	214.3
FTHFD_HUMAN	10-formyltetrahydrofolate dehydrogenase	**3**	186.8	**3**	150.1
GLYC_HUMAN	Serine hydroxymethyltransferase, cytosolic	**3**	194.2	**2**	138.4
NAGAB_HUMAN	Alpha-N-acetylgalactosaminidase	**4**	205.2	**3**	126.6
PDC6I_HUMAN	Programmed cell death 6-interacting protein	**4**	195.6	**3**	136.1
PCD12_HUMAN	Protocadherin-12	**2**	192.8	**3**	137.4
CD81_HUMAN	CD81 antigen	**3**	202.4	**2**	125.4
TRHDE_HUMAN	Thyrotropin-releasing hormone-degrading ectoenzyme	**3**	112.6	**5**	215

Table S2. *Cont.*

Accession	Protein Name	AAL1 #Peptides	AAL1 Score	AAL2 #Peptides	AAL2 Score
ANGL2_HUMAN	Angiopoietin-related protein 2	**2**	99.4	**6**	223.8
C1R_HUMAN	Complement C1r subcomponent	**2**	104.1	**5**	218.9
HV304_HUMAN	Ig heavy chain V-III region TIL	**2**	156.2	**2**	166.4
RINI_HUMAN	Ribonuclease inhibitor	**2**	138.7	**3**	182.9
VNN1_HUMAN	Pantetheinase	**2**	135.7	**3**	183.4
AL1A1_HUMAN	Retinal dehydrogenase 1	**3**	122.5	**5**	186.6
MUCDL_HUMAN	Mucin and cadherin-like protein	**2**	100.1	**3**	207.3
MXRA8_HUMAN	Matrix-remodeling-associated protein 8	**1**	65.6	**4**	241
KV104_HUMAN	Ig kappa chain V-I region CAR	**2**	181.5	**1**	116.8
NAPSA_HUMAN	Napsin-A	**2**	160.5	**2**	133.1
MMRN2_HUMAN	Multimerin-2	**2**	158.5	**2**	133.1
GP1BA_HUMAN	Platelet glycoprotein Ib alpha chain	**2**	69.6	**5**	221.1
GNS_HUMAN	N-acetylglucosamine-6-sulfatase	**2**	107	**4**	183.3
PAG15_HUMAN	Group XV phospholipase A2	**2**	116	**3**	173.3
ALS_HUMAN	Insulin-like growth factor-binding protein complex acid labile subunit	**3**	161.5	**3**	126.3
BCAM_HUMAN	Basal cell adhesion molecule	**3**	120.6	**4**	165.9
C1RL_HUMAN	Complement C1r subcomponent-like protein	**2**	142.9	**2**	139.1
G6PI_HUMAN	Glucose-6-phosphate isomerase	**3**	150.5	**2**	130.9
SAMP_HUMAN	Serum amyloid P-component	**3**	165.8	**2**	114.1
LDHA_HUMAN	L-lactate dehydrogenase A chain	**2**	144.6	**2**	134.6
DCD_HUMAN	Dermcidin	**3**	179.4	**2**	99.6
R4RL2_HUMAN	Reticulon-4 receptor-like 2	**2**	127.9	**2**	150.2
ASSY_HUMAN	Argininosuccinate synthase	**2**	103.9	**3**	173.6
WFDC2_HUMAN	WAP four-disulfide core domain protein 2	**3**	119.4	**4**	154.8
PPGB_HUMAN	Lysosomal protective protein	**2**	122.4	**2**	150.5
SCRB2_HUMAN	Lysosome membrane protein 2	**3**	147.6	**2**	125.2
A1AG1_HUMAN	Alpha-1-acid glycoprotein 1	**4**	171.2	**2**	100.6
HV305_HUMAN	Ig heavy chain V-III region BRO	**2**	121.5	**2**	149.3
MA2B2_HUMAN	Epididymis-specific alpha-mannosidase	**2**	102.2	**3**	165.6
AT1A1_HUMAN	Sodium/potassium-transporting ATPase subunit alpha-1	**3**	169.2	**2**	98.2
CO8G_HUMAN	Complement component C8 gamma chain	**2**	93	**3**	173.7
TPP1_HUMAN	Tripeptidyl-peptidase 1	**2**	133.5	**2**	131.6
CD44_HUMAN	CD44 antigen	**2**	105.5	**2**	158

Table S2. *Cont.*

Accession	Protein Name	AAL1 #Peptides	AAL1 Score	AAL2 #Peptides	AAL2 Score
K2C78_HUMAN	Keratin, type II cytoskeletal 78	**2**	147.5	**2**	113.9
PTPRG_HUMAN	Receptor-type tyrosine-protein phosphatase gamma	**3**	141	**4**	120.1
C1QC_HUMAN	Complement C1q subcomponent subunit C	**1**	129.9	**2**	130.6
UBA1_HUMAN	Ubiquitin-like modifier-activating enzyme 1	**2**	105.4	**3**	145.7
F16P1_HUMAN	Fructose-1,6-bisphosphatase 1	**3**	132.2	**2**	118.9
PGM1_HUMAN	Phosphoglucomutase-1	**2**	113.3	**2**	137.4
DNAS1_HUMAN	Deoxyribonuclease-1	**3**	184.4	**1**	63.1
HV306_HUMAN	Ig heavy chain V-III region BUT	**1**	101.4	**3**	145
CATC_HUMAN	Dipeptidyl peptidase 1	**2**	131.6	**1**	113.2
YS019_HUMAN	Transmembrane protein HSPC323	**3**	150.6	**2**	92.4
MANBA_HUMAN	Beta-mannosidase	**2**	87.8	**3**	154.6
RNAS2_HUMAN	Non-secretory ribonuclease	**2**	121	**2**	120.4
MA2B1_HUMAN	Lysosomal alpha-mannosidase	**2**	117.7	**3**	123.4
ICOSL_HUMAN	ICOS ligand	**2**	127	**2**	113.7
GP126_HUMAN	G-protein coupled receptor 126	**2**	116.3	**2**	123.7
GLCM_HUMAN	Glucosylceramidase	**2**	129.7	**2**	110.3
PERM_HUMAN	Myeloperoxidase	**2**	86.4	**3**	152
PODXL_HUMAN	Podocalyxin-like protein 1	**2**	113.9	**2**	123
PRG2_HUMAN	Bone marrow proteoglycan	**3**	103.9	**3**	132.6
VAT1_HUMAN	Synaptic vesicle membrane protein VAT-1 homolog	**2**	79.5	**3**	157
MDHC_HUMAN	Malate dehydrogenase, cytoplasmic	**3**	139.3	**2**	96.1
KLK1_HUMAN	Kallikrein-1	**3**	102.4	**3**	131.2
LYAM1_HUMAN	L-selectin	**2**	107.5	**2**	125
CBR1_HUMAN	Carbonyl reductase [NADPH] 1	**2**	64.4	**3**	159.5
L1CAM_HUMAN	Neural cell adhesion molecule L1	**3**	139.8	**2**	82.1
B4GT1_HUMAN	Beta-1,4-galactosyltransferase 1	**1**	78.2	**2**	143.6
DSG1_HUMAN	Desmoglein-1	**4**	189.2	**1**	31.6
CLC14_HUMAN	C-type lectin domain family 14 member A	**2**	87.2	**3**	132.9
DDR1_HUMAN	Epithelial discoidin domain-containing receptor 1	**2**	97.6	**2**	121.6
EPHB4_HUMAN	Ephrin type-B receptor 4	**2**	132.4	**1**	86.5
LKHA4_HUMAN	Leukotriene A-4 hydrolase	**3**	100.1	**3**	117.8
CNDP2_HUMAN	Cytosolic non-specific dipeptidase	**3**	147.5	**2**	66.9
CD5L_HUMAN	CD5 antigen-like	**1**	74.6	**2**	137

Table S2. *Cont.*

Accession	Protein Name	AAL1 #Peptides	AAL1 Score	AAL2 #Peptides	AAL2 Score
FCG3A_HUMAN	Low affinity immunoglobulin gamma Fc region receptor III-A	**2**	91.6	**2**	119.3
KAIN_HUMAN	Kallistatin	**1**	35.9	**4**	173.7
GDIB_HUMAN	Rab GDP dissociation inhibitor beta	**2**	85	**2**	124.4
PLAK_HUMAN	Junction plakoglobin	**2**	122.7	**2**	83.6
ASAH1_HUMAN	Acid ceramidase	**2**	114.8	**2**	89.2
THY1_HUMAN	Thy-1 membrane glycoprotein	**2**	101.2	**2**	100.4
GGH_HUMAN	Gamma-glutamyl hydrolase	**2**	96.3	**2**	103.1
EF2_HUMAN	Elongation factor 2	**3**	153.2	**1**	44.8
ARP2_HUMAN	Actin-related protein 2	**2**	125.7	**2**	71.8
CO6A3_HUMAN	Collagen alpha-3(VI) chain	**1**	69.1	**2**	128.1
PTPRK_HUMAN	Receptor-type tyrosine-protein phosphatase kappa	**3**	149.7	**1**	47.3
SAHH_HUMAN	Adenosylhomocysteinase	**3**	116.1	**2**	77.5
LTBP2_HUMAN	Latent-transforming growth factor beta-binding protein 2	**2**	72.3	**2**	121
H2B1K_HUMAN	Histone H2B type 1-K	**2**	130.9	**1**	61.7
FAT4_HUMAN	Protocadherin Fat 4	**1**	48.7	**3**	142.2
APOL1_HUMAN	Apolipoprotein L1	**1**	97.2	**1**	90.1
MUC18_HUMAN	Cell surface glycoprotein MUC18	**3**	91	**2**	96.1
IGJ_HUMAN	Immunoglobulin J chain	**1**	67.7	**2**	118
FUCO_HUMAN	Tissue alpha-L-fucosidase	**2**	109.8	**2**	75.8
FGL2_HUMAN	Fibroleukin	**2**	52.2	**3**	132.8
HV207_HUMAN	Ig heavy chain V-II region NEWM	**1**	73	**1**	109.5
PLTP_HUMAN	Phospholipid transfer protein	**2**	110.8	**1**	70.8
LBP_HUMAN	Lipopolysaccharide-binding protein	**2**	98.3	**1**	81.6
UBIQ_HUMAN	Ubiquitin	**1**	87	**2**	91.4
C1S_HUMAN	Complement C1s subcomponent	**1**	61.3	**2**	117
TENX_HUMAN	Tenascin-X	**2**	92.7	**2**	84.8
HV301_HUMAN	Ig heavy chain V-III region TRO	**1**	58.6	**2**	118.6
PRDX6_HUMAN	Peroxiredoxin-6	**1**	49.1	**2**	126.6
TRBM_HUMAN	Thrombomodulin	**1**	73.5	**2**	101.3
DSC2_HUMAN	Desmocollin-2	**3**	106.5	**2**	68.2
S10A8_HUMAN	Protein S100-A8	**2**	84.8	**2**	89.5
S36A2_HUMAN	Proton-coupled amino acid transporter 2	**2**	73.3	**2**	98.7
TTHY_HUMAN	Transthyretin	**1**	67.4	**2**	103.6
PGM2_HUMAN	Phosphoglucomutase-2	**2**	73.7	**2**	97.2

Table S2. *Cont.*

Accession	Protein Name	AAL1 #Peptides	AAL1 Score	AAL2 #Peptides	AAL2 Score
CAZA1_HUMAN	F-actin-capping protein subunit alpha-1	**2**	70.9	**2**	99.4
1433E_HUMAN	14-3-3 protein epsilon	**2**	97.4	**2**	72.8
ITB1_HUMAN	Integrin beta-1	**2**	103.3	**1**	64.6
A4_HUMAN	Amyloid beta A4 protein	**1**	31.3	**2**	135.6
CRYL1_HUMAN	Lambda-crystallin homolog	**1**	73.7	**1**	92.7
ANXA3_HUMAN	Annexin A3	**1**	33.2	**2**	130.5
S10A9_HUMAN	Protein S100-A9	**1**	47.7	**2**	115
P3IP1_HUMAN	Phosphoinositide-3-kinase-interacting protein 1	**2**	83.1	**2**	77.6
EF1A1_HUMAN	Elongation factor 1-alpha 1	**1**	65.1	**2**	95.4
FUCO2_HUMAN	Plasma alpha-L-fucosidase	**2**	99	**1**	60.2
ACPH_HUMAN	Acylamino-acid-releasing enzyme	**2**	99.3	**1**	57.7
PGAM1_HUMAN	Phosphoglycerate mutase 1	**1**	68.1	**2**	86
TARSH_HUMAN	Target of Nesh-SH3	**1**	32.1	**3**	121.8
CAMP_HUMAN	Cathelicidin antimicrobial peptide	**2**	75.9	**2**	75.7
CATZ_HUMAN	Cathepsin Z	**2**	93	**1**	57.9
PDIA1_HUMAN	Protein disulfide-isomerase	**1**	64.1	**1**	84.9
CBLN2_HUMAN	Cerebellin-2	**2**	113.3	**1**	35.4
EPCR_HUMAN	Endothelial protein C receptor	**1**	68.9	**2**	78.7
DHAK_HUMAN	Bifunctional ATP-dependent dihydroxyacetone kinase/FAD-AMP lyase (cyclizing)	**1**	45.6	**2**	101.5
MVP_HUMAN	Major vault protein	**2**	103.4	**1**	43
AGRIN_HUMAN	Agrin	**1**	46.5	**2**	99.7
TKT_HUMAN	Transketolase	**1**	40.6	**2**	105.3
TCO1_HUMAN	Transcobalamin-1	**1**	34	**2**	110.3
CTL4_HUMAN	Choline transporter-like protein 4	**1**	68.3	**1**	75.1
ARSF_HUMAN	Arylsulfatase F	**1**	68.1	**1**	74.4
OSTP_HUMAN	Osteopontin	**2**	90.3	**1**	51.8
GPC5C_HUMAN	G-protein coupled receptor family C group 5 member C	**1**	73.2	**1**	68.5
XPP2_HUMAN	Xaa-Pro aminopeptidase 2	**1**	47	**2**	94.6
PVRL4_HUMAN	Poliovirus receptor-related protein 4	**1**	59.7	**1**	80.9
LAMP1_HUMAN	Lysosome-associated membrane glycoprotein 1	**1**	61.5	**1**	78.9
ANPRC_HUMAN	Atrial natriuretic peptide receptor 3	**2**	104.4	**1**	33.8
PCP_HUMAN	Lysosomal Pro-X carboxypeptidase	**3**	112	**1**	25.8

Table S2. *Cont.*

Accession	Protein Name	AAL1 #Peptides	AAL1 Score	AAL2 #Peptides	AAL2 Score
SERC1_HUMAN	Serine incorporator 1	**1**	46.8	**2**	90.9
TIG1_HUMAN	Retinoic acid receptor responder protein 1	**1**	72	**2**	65.6
BPI_HUMAN	Bactericidal permeability-increasing protein	**1**	60.8	**1**	76.8
LIPL_HUMAN	Lipoprotein lipase	**1**	57.3	**1**	80
CAH2_HUMAN	Carbonic anhydrase 2	**1**	36.7	**2**	96.2
KLK3_HUMAN	Prostate-specific antigen	**1**	34.7	**2**	97.9
TPIS_HUMAN	Triosephosphate isomerase	**1**	51	**2**	81.5
RENR_HUMAN	Renin receptor	**1**	57	**2**	75.4
HYAL1_HUMAN	Hyaluronidase-1	**2**	81	**2**	50.4
ARP3_HUMAN	Actin-related protein 3	**2**	77.9	**1**	52.2
PGS1_HUMAN	Biglycan	**1**	68.3	**1**	60.7
AATC_HUMAN	Aspartate aminotransferase, cytoplasmic	**2**	58.3	**2**	70.4
QPCT_HUMAN	Glutaminyl-peptide cyclotransferase	**2**	93.4	**1**	35.1
CAN1_HUMAN	Calpain-1 catalytic subunit	**1**	43.2	**2**	84.6
PIP_HUMAN	Prolactin-inducible protein	**1**	26.6	**2**	99.8
CD9_HUMAN	CD9 antigen	**1**	61	**1**	65.3
FILA2_HUMAN	Filaggrin-2	**1**	81.7	**1**	44.5
DNER_HUMAN	Delta and Notch-like epidermal growth factor-related receptor	**1**	62.9	**1**	62.7
C4BPA_HUMAN	C4b-binding protein alpha chain	**1**	60.1	**1**	65.5
SYPL1_HUMAN	Synaptophysin-like protein 1	**1**	62.2	**1**	62.4
PCKGC_HUMAN	Phosphoenolpyruvate carboxykinase, cytosolic [GTP]	**1**	52.6	**2**	70.7
AMPL_HUMAN	Cytosol aminopeptidase	**1**	68.6	**1**	54.2
PDZ1P_HUMAN	Putative PDZ domain-containing protein 1P	**1**	67.8	**1**	54.9
VTDB_HUMAN	Vitamin D-binding protein	**1**	35.6	**2**	86.9
GSTP1_HUMAN	Glutathione S-transferase P	**1**	42.9	**2**	79.2
6PGD_HUMAN	6-phosphogluconate dehydrogenase, decarboxylating	**1**	31.6	**3**	89.4
GUAD_HUMAN	Guanine deaminase	**2**	81.2	**1**	38.9
TERA_HUMAN	Transitional endoplasmic reticulum ATPase	**1**	43	**2**	76.4
CD63_HUMAN	CD63 antigen	**1**	50	**1**	67.4
ISLR_HUMAN	Immunoglobulin superfamily containing leucine-rich repeat protein	**2**	74.4	**1**	42.6

Table S2. *Cont.*

Accession	Protein Name	AAL1 #Peptides	AAL1 Score	AAL2 #Peptides	AAL2 Score
TM7S3_HUMAN	Transmembrane 7 superfamily member 3	**1**	53	**1**	64
A2AP_HUMAN	Alpha-2-antiplasmin	**2**	76.4	**1**	40.2
NAR3_HUMAN	Ecto-ADP-ribosyltransferase 3	**1**	48.5	**1**	66.9
HV102_HUMAN	Ig heavy chain V-I region HG3	**1**	60.5	**1**	54.7
LEG9_HUMAN	Galectin-9	**1**	44.7	**1**	69.7
SBP1_HUMAN	Selenium-binding protein 1	**3**	86.8	**1**	25.6
JAG1_HUMAN	Protein jagged-1	**1**	54	**1**	58.4
LAMC1_HUMAN	Laminin subunit gamma-1	**1**	64.4	**1**	48
HEXA_HUMAN	Beta-hexosaminidase subunit alpha	**1**	38.8	**1**	71.9
RISC_HUMAN	Retinoid-inducible serine carboxypeptidase	**1**	36.6	**2**	73.9
AMNLS_HUMAN	Protein amnionless	**1**	56.9	**1**	52.4
SDC1_HUMAN	Syndecan-1	**1**	67.4	**1**	39.5
SDCB1_HUMAN	Syntenin-1	**2**	65.7	**2**	40.9
KV203_HUMAN	Ig kappa chain V-II region MIL	**1**	54.4	**1**	51.7
TTYH3_HUMAN	Protein tweety homolog 3	**1**	54.6	**1**	50.9
HABP2_HUMAN	Hyaluronan-binding protein 2	**1**	44.5	**1**	60.3
HGFA_HUMAN	Hepatocyte growth factor activator	**1**	49.6	**1**	54.2
ML12B_HUMAN	Myosin regulatory light chain 12B	**1**	60.2	**1**	43.2
CRUM2_HUMAN	Crumbs homolog 2	**1**	47.2	**1**	51.4
CBPE_HUMAN	Carboxypeptidase E	**1**	34.9	**2**	62.6
FBN1_HUMAN	Fibrillin-1	**1**	46.4	**1**	49.5
SAP_HUMAN	Proactivator polypeptide	**1**	30.3	**1**	65.4
CEL_HUMAN	Bile salt-activated lipase	**1**	46.9	**1**	48.2
CADH5_HUMAN	Cadherin-5	**1**	54.2	**1**	40.6
LAMA5_HUMAN	Laminin subunit alpha-5	**1**	40.4	**1**	54
ANXA4_HUMAN	Annexin A4	**1**	29.8	**1**	64.2
LAMP2_HUMAN	Lysosome-associated membrane glycoprotein 2	**1**	47.4	**1**	46.3
ARPC4_HUMAN	Actin-related protein 2/3 complex subunit 4	**1**	32.9	**1**	60.5
CLN5_HUMAN	Ceroid-lipofuscinosis neuronal protein 5	**1**	48.8	**1**	43.2
CATB_HUMAN	Cathepsin B	**1**	41	**1**	50.8
KV309_HUMAN	Ig kappa chain V-III region VG (Fragment)	**1**	44.8	**1**	45.5

Table S2. *Cont.*

Accession	Protein Name	AAL1 #Peptides	AAL1 Score	AAL2 #Peptides	AAL2 Score
GBG12_HUMAN	Guanine nucleotide-binding protein G(I)/G(S)/G(O) subunit gamma-12	**1**	56	**1**	33.8
SPRL1_HUMAN	SPARC-like protein 1	**1**	32	**1**	57.4
MYO6_HUMAN	Myosin-VI	**1**	48.9	**1**	40.3
NRP1_HUMAN	Neuropilin-1	**1**	32.8	**1**	55.7
MMRN1_HUMAN	Multimerin-1	**1**	49.7	**1**	38.3
VMO1_HUMAN	Vitelline membrane outer layer protein 1 homolog	**1**	42.4	**1**	43
ATPB_HUMAN	ATP synthase subunit beta, mitochondrial	**1**	44.7	**1**	40.3
DSG2_HUMAN	Desmoglein-2	**1**	38.6	**1**	46
F13A_HUMAN	Coagulation factor XIII A chain	**1**	36.8	**1**	46.8
BODG_HUMAN	Gamma-butyrobetaine dioxygenase	**1**	56.2	**1**	27.4
PPT1_HUMAN	Palmitoyl-protein thioesterase 1	**1**	58.4	**1**	25
PEPA_HUMAN	Pepsin A	**1**	41.1	**1**	40.7
APOA_HUMAN	Apolipoprotein(a)	**1**	35.4	**1**	46.1
HRG_HUMAN	Histidine-rich glycoprotein	**2**	53	**1**	27.5
CBPB2_HUMAN	Carboxypeptidase B2	**1**	44.8	**1**	35.4
IGHD_HUMAN	Ig delta chain C region	**1**	36.2	**1**	43.9
ILEU_HUMAN	Leukocyte elastase inhibitor	**1**	39.1	**1**	40.9
CD59_HUMAN	CD59 glycoprotein	**1**	33.2	**1**	44.3
LIRA3_HUMAN	Leukocyte immunoglobulin-like receptor subfamily A member 3	**1**	32	**1**	44.4
F151A_HUMAN	Protein FAM151A	**1**	29.6	**1**	46.4
EPHB6_HUMAN	Ephrin type-B receptor 6	**1**	32.4	**1**	43.6
SORT_HUMAN	Sortilin	**1**	28.4	**1**	47.4
AL9A1_HUMAN	4-trimethylaminobutyraldehyde dehydrogenase	**1**	33.1	**1**	41.3
EPHAA_HUMAN	Ephrin type-A receptor 10	**1**	33.1	**1**	40.8
HSPB1_HUMAN	Heat shock protein beta-1	**1**	38.2	**1**	35.1
CATG_HUMAN	Cathepsin G	**1**	27.6	**1**	45.1
PTTG_HUMAN	Pituitary tumor-transforming gene 1 protein-interacting protein	**1**	37.3	**1**	34.9
PLD3_HUMAN	Phospholipase D3	**1**	29.3	**1**	42.6
PNPH_HUMAN	Purine nucleoside phosphorylase	**1**	39.6	**1**	31.5
BDH2_HUMAN	3-hydroxybutyrate dehydrogenase type 2	**1**	29	**1**	40.1
MDR1_HUMAN	Multidrug resistance protein 1	**1**	26.9	**1**	41.5
8ODP_HUMAN	7,8-dihydro-8-oxoguanine triphosphatase	**1**	28.4	**1**	39.2

Table S2. *Cont.*

Accession	Protein Name	AAL1 #Peptides	AAL1 Score	AAL2 #Peptides	AAL2 Score
ESTD_HUMAN	S-formylglutathione hydrolase	**1**	34.7	**1**	31.7
PEBP1_HUMAN	Phosphatidylethanolamine-binding protein 1	**1**	31	**1**	35.1
PPT2_HUMAN	Lysosomal thioesterase PPT2	**1**	30.3	**1**	34.7
WDR1_HUMAN	WD repeat-containing protein 1	**1**	35.2	**1**	29
ARC1B_HUMAN	Actin-related protein 2/3 complex subunit 1B	**1**	29.2	**1**	34.3
TACD2_HUMAN	Tumor-associated calcium signal transducer 2	**1**	36.1	**1**	26.4
GPX3_HUMAN	Glutathione peroxidase 3	**1**	25.8	**1**	36
TRY1_HUMAN	Trypsin-1	**1**	28.7	**1**	32.3
CATO_HUMAN	Cathepsin O	**1**	29.1	**1**	31.7
GALNS_HUMAN	N-acetylgalactosamine-6-sulfatase	**1**	25.9	**1**	34.1
GDIR1_HUMAN	Rho GDP-dissociation inhibitor 1	**1**	29.4	**1**	30.4
CPXM2_HUMAN	Inactive carboxypeptidase-like protein X2	**1**	29.9	**1**	28
SAP3_HUMAN	Ganglioside GM2 activator	**1**	28	**1**	27.6
GPVI_HUMAN	Platelet glycoprotein VI	**1**	26.7	**1**	27.8

Table S3. Proteins identified in the AAL flow-through from pTa urine in both experimental replicates. Proteins are sorted according to their average score.

Accession	Protein Name	FT1 #Peptides	FT1 Score	FT2 #Peptides	FT2 Score
ALBU_HUMAN	Serum albumin	**88**	6766.5	**86**	6551.7
TRFE_HUMAN	Serotransferrin	**60**	4412.3	**60**	4740.9
CO3_HUMAN	Complement C3	**57**	3543.8	**54**	3310.8
A2MG_HUMAN	Alpha-2-macroglobulin	**50**	3097.2	**46**	3061.8
CERU_HUMAN	Ceruloplasmin	**45**	3048.6	**41**	3005.1
AMBP_HUMAN	Protein AMBP	**33**	2938.5	**32**	2949.6
FIBB_HUMAN	Fibrinogen beta chain	**38**	2510.2	**28**	2182
IGKC_HUMAN	Ig kappa chain C region	**20**	2230.6	**17**	1977
VTDB_HUMAN	Vitamin D-binding protein	**24**	1969.4	**26**	2060.5
FIBG_HUMAN	Fibrinogen gamma chain	**28**	1752.1	**28**	1897
A1AT_HUMAN	Alpha-1-antitrypsin	**24**	1843.2	**20**	1687.8
PIGR_HUMAN	Polymeric immunoglobulin receptor	**24**	1774.5	**20**	1553.3
CFAH_HUMAN	Complement factor H	**31**	1741.2	**24**	1560.3
CO4B_HUMAN	Complement C4-B	**30**	1682.5	**27**	1596.6
KNG1_HUMAN	Kininogen-1	**26**	1774.6	**22**	1471.4
IGHG1_HUMAN	Ig gamma-1 chain C region	**22**	1575	**22**	1561.3

Table S3. *Cont.*

Accession	Protein Name	FT1 #Peptides	FT1 Score	FT2 #Peptides	FT2 Score
FIBA_HUMAN	Fibrinogen alpha chain	**24**	1580	**21**	1406.2
GELS_HUMAN	Gelsolin	**22**	1390.5	**20**	1437.8
AMYP_HUMAN	Pancreatic alpha-amylase	**24**	1677.2	**13**	935.5
K2C1_HUMAN	Keratin, type II cytoskeletal 1	**25**	1554.7	**17**	1017.8
K1C10_HUMAN	Keratin, type I cytoskeletal 10	**29**	1734.6	**12**	733.8
AMY1_HUMAN	Alpha-amylase 1	**22**	1490.4	**14**	942.8
AMY2B_HUMAN	Alpha-amylase 2B	**20**	1445.2	**13**	899.5
HEMO_HUMAN	Hemopexin	**22**	1169.2	**20**	1157.4
OSTP_HUMAN	Osteopontin	**16**	1080	**18**	1162.4
ITIH4_HUMAN	Inter-alpha-trypsin inhibitor heavy chain H4	**16**	1309.3	**13**	903.4
HPT_HUMAN	Haptoglobin	**14**	905.7	**21**	1251.2
APOB_HUMAN	Apolipoprotein B-100	**21**	1016.5	**22**	1083.3
ACTB_HUMAN	Actin, cytoplasmic 1	**16**	1002	**16**	1082.3
K22E_HUMAN	Keratin, type II cytoskeletal 2 epidermal	**27**	1670.8	**7**	411.3
TRFL_HUMAN	Lactotransferrin	**22**	1202.3	**13**	879.4
A1AG1_HUMAN	Alpha-1-acid glycoprotein 1	**16**	1222.1	**13**	849.2
UROM_HUMAN	Uromodulin	**17**	1026	**17**	1027.4
FINC_HUMAN	Fibronectin	**22**	1081.6	**17**	949.5
K1C9_HUMAN	Keratin, type I cytoskeletal 9	**12**	821.3	**14**	1160.9
APOA1_HUMAN	Apolipoprotein A-I	**18**	929.6	**18**	1023.4
IGHG3_HUMAN	Ig gamma-3 chain C region	**17**	982	**17**	963.5
PLMN_HUMAN	Plasminogen	**19**	1073.4	**13**	842.1
IGHG4_HUMAN	Ig gamma-4 chain C region	**13**	983	**14**	922
THRB_HUMAN	Prothrombin	**13**	900.6	**12**	989.9
EGF_HUMAN	Pro-epidermal growth factor	**17**	967.4	**13**	913.9
CADH1_HUMAN	Cadherin-1	**12**	1027.9	**10**	851.7
IGHA1_HUMAN	Ig alpha-1 chain C region	**12**	773.4	**16**	1077.8
IGHG2_HUMAN	Ig gamma-2 chain C region	**15**	810.1	**19**	1034.2
APOD_HUMAN	Apolipoprotein D	**14**	885.9	**13**	794.6
HBB_HUMAN	Hemoglobin subunit beta	**12**	754.5	**12**	887.1
LMAN2_HUMAN	Vesicular integral-membrane protein VIP36	**13**	827.7	**13**	807.5
ZA2G_HUMAN	Zinc-alpha-2-glycoprotein	**13**	764.6	**13**	863.6
AACT_HUMAN	Alpha-1-antichymotrypsin	**12**	742.3	**12**	885.3
AMPN_HUMAN	Aminopeptidase N	**17**	895.7	**14**	711.9
ENDD1_HUMAN	Endonuclease domain-containing 1 protein	**9**	718.2	**10**	840.8
IGHA2_HUMAN	Ig alpha-2 chain C region	**9**	619.2	**12**	933.8
CFAI_HUMAN	Complement factor I	**9**	818	**8**	614.6

Table S3. *Cont.*

**Accession**	**Protein Name**	**FT1 #Peptides**	**FT1 Score**	**FT2 #Peptides**	**FT2 Score**
CUBN_HUMAN	Cubilin	**13**	786.4	**10**	634.1
PGBM_HUMAN	Basement membrane-specific heparan sulfate proteoglycan core protein	**9**	600.9	**12**	762.9
CLUS_HUMAN	Clusterin	**12**	695.5	**9**	637.7
IGHM_HUMAN	Ig mu chain C region	**8**	584.2	**11**	748.5
ANT3_HUMAN	Antithrombin-III	**13**	607.3	**11**	687
CFAB_HUMAN	Complement factor B	**11**	517.3	**13**	729.7
CATD_HUMAN	Cathepsin D	**9**	528.4	**11**	700.4
ANAG_HUMAN	Alpha-N-acetylglucosaminidase	**10**	595.4	**12**	632.9
A1BG_HUMAN	Alpha-1B-glycoprotein	**10**	537.4	**11**	686
AFAM_HUMAN	Afamin	**11**	521.9	**12**	697.7
PTGDS_HUMAN	Prostaglandin-H2 D-isomerase	**8**	663.8	**5**	554.8
APOA4_HUMAN	Apolipoprotein A-IV	**11**	575.8	**10**	639
FBLN1_HUMAN	Fibulin-1	**6**	409.1	**10**	782
PI16_HUMAN	Peptidase inhibitor 16	**10**	665.4	**6**	512.3
PAPP2_HUMAN	Pappalysin-2	**11**	563.1	**10**	582.7
GNS_HUMAN	N-acetylglucosamine-6-sulfatase	**9**	503.2	**11**	620.6
A1AG2_HUMAN	Alpha-1-acid glycoprotein 2	**10**	621.9	**8**	501.5
PPAP_HUMAN	Prostatic acid phosphatase	**9**	577.6	**8**	514.9
ITIH1_HUMAN	Inter-alpha-trypsin inhibitor heavy chain H1	**10**	509.3	**7**	580.6
CD14_HUMAN	Monocyte differentiation antigen CD14	**6**	429.6	**8**	648.3
MYH9_HUMAN	Myosin-9	**10**	642.7	**7**	430.8
FBLN3_HUMAN	EGF-containing fibulin-like extracellular matrix protein 1	**8**	377.1	**10**	692.7
IC1_HUMAN	Plasma protease C1 inhibitor	**7**	429.8	**11**	636.4
IPSP_HUMAN	Plasma serine protease inhibitor	**8**	477.9	**8**	586.1
LYAG_HUMAN	Lysosomal alpha-glucosidase	**7**	375.2	**11**	660.5
APOE_HUMAN	Apolipoprotein E	**11**	592.1	**6**	435.1
HBD_HUMAN	Hemoglobin subunit delta	**7**	418.8	**9**	603.6
CADH2_HUMAN	Cadherin-2	**8**	486.5	**8**	532.5
HPTR_HUMAN	Haptoglobin-related protein	**8**	383.6	**11**	614.5
FETUA_HUMAN	Alpha-2-HS-glycoprotein	**7**	474.3	**7**	519.3
VCAM1_HUMAN	Vascular cell adhesion protein 1	**9**	446.2	**8**	494.8
ITIH2_HUMAN	Inter-alpha-trypsin inhibitor heavy chain H2	**9**	436.7	**9**	486.1
CO6A1_HUMAN	Collagen alpha-1(VI) chain	**10**	457.3	**8**	455.5
APOH_HUMAN	Beta-2-glycoprotein 1	**6**	378.6	**7**	513.9
LG3BP_HUMAN	Galectin-3-binding protein	**10**	613.2	**5**	272
HMCN1_HUMAN	Hemicentin-1	**7**	408.5	**8**	464.7

Table S3. *Cont.*

**Accession**	**Protein Name**	**FT1 #Peptides**	**FT1 Score**	**FT2 #Peptides**	**FT2 Score**
LRP2_HUMAN	Low-density lipoprotein receptor-related protein 2	**5**	271.6	**10**	594.7
ATRN_HUMAN	Attractin	**11**	534.5	**4**	330
THBG_HUMAN	Thyroxine-binding globulin	**6**	404	**6**	459.6
A2GL_HUMAN	Leucine-rich alpha-2-glycoprotein	**7**	473.7	**5**	389.6
VASN_HUMAN	Vasorin	**8**	517	**5**	344
ALDOB_HUMAN	Fructose-bisphosphate aldolase B	**7**	432.7	**6**	401.2
KLK3_HUMAN	Prostate-specific antigen	**6**	436.1	**9**	378.8
BGAL_HUMAN	Beta-galactosidase	**8**	430.7	**6**	362.7
CADM4_HUMAN	Cell adhesion molecule 4	**4**	346.5	**5**	437.6
VMO1_HUMAN	Vitelline membrane outer layer protein 1 homolog	**5**	335.4	**5**	436.3
PERM_HUMAN	Myeloperoxidase	**8**	382.9	**7**	378.2
DSC2_HUMAN	Desmocollin-2	**7**	414.3	**6**	332.7
S10A9_HUMAN	Protein S100-A9	**6**	291.6	**7**	455.2
TETN_HUMAN	Tetranectin	**7**	419.4	**6**	326.4
MASP2_HUMAN	Mannan-binding lectin serine protease 2	**6**	439.8	**4**	293.8
SPRL1_HUMAN	SPARC-like protein 1	**7**	381.6	**7**	335.2
ENOA_HUMAN	Alpha-enolase	**7**	329.5	**5**	373.4
CAD13_HUMAN	Cadherin-13	**7**	416.2	**4**	271.7
ANXA2_HUMAN	Annexin A2	**5**	303.7	**6**	381.4
MGA_HUMAN	Maltase-glucoamylase, intestinal	**5**	293.2	**7**	384.7
CO7_HUMAN	Complement component C7	**6**	368.2	**4**	306
KV101_HUMAN	Ig kappa chain V-I region AG	**3**	342.8	**3**	318.1
KPYM_HUMAN	Pyruvate kinase isozymes M1/M2	**6**	297	**6**	363.8
ARSA_HUMAN	Arylsulfatase A	**7**	356	**5**	298.9
AGAL_HUMAN	Alpha-galactosidase A	**5**	235	**5**	402.3
QPCT_HUMAN	Glutaminyl-peptide cyclotransferase	**5**	281	**6**	348
EPCR_HUMAN	Endothelial protein C receptor	**5**	284.7	**4**	333.9
DNAS1_HUMAN	Deoxyribonuclease-1	**5**	334.2	**3**	278.9
PLSL_HUMAN	Plastin-2	**6**	240.2	**7**	363.9
ACTN4_HUMAN	Alpha-actinin-4	**4**	214.2	**4**	387.5
KLK1_HUMAN	Kallikrein-1	**5**	257.5	**5**	323.5
HBA_HUMAN	Hemoglobin subunit alpha	**6**	287.6	**7**	289.6
RET4_HUMAN	Retinol-binding protein 4	**5**	322.1	**4**	253.9
KV402_HUMAN	Ig kappa chain V-IV region Len	**4**	282.8	**3**	292.8
CYTC_HUMAN	Cystatin-C	**3**	262.6	**4**	308.1
CAH1_HUMAN	Carbonic anhydrase 1	**6**	283.2	**5**	277.3
C1S_HUMAN	Complement C1s subcomponent	**6**	271.8	**5**	286.9
CAD11_HUMAN	Cadherin-11	**4**	196.8	**7**	356.3

Table S3. *Cont.*

**Accession**	**Protein Name**	**FT1 #Peptides**	**FT1 Score**	**FT2 #Peptides**	**FT2 Score**
HEG1_HUMAN	Protein HEG homolog 1	**4**	314.3	**3**	238
GSTP1_HUMAN	Glutathione S-transferase P	**6**	267.2	**5**	283.6
VTNC_HUMAN	Vitronectin	**6**	281	**5**	262.7
HRG_HUMAN	Histidine-rich glycoprotein	**8**	330.4	**5**	211.8
PGRP1_HUMAN	Peptidoglycan recognition protein 1	**4**	261.8	**3**	278
PEDF_HUMAN	Pigment epithelium-derived factor	**3**	170.7	**5**	366.3
LAC_HUMAN	Ig lambda chain C regions	**4**	291.6	**3**	244.9
PEBP1_HUMAN	Phosphatidylethanolamine-binding protein 1	**4**	279.8	**3**	250.5
AGRIN_HUMAN	Agrin	**3**	190	**5**	331.5
P3IP1_HUMAN	Phosphoinositide-3-kinase-interacting protein 1	**5**	248.2	**5**	270.9
PON1_HUMAN	Serum paraoxonase/arylesterase 1	**5**	252.6	**4**	259.7
CO6_HUMAN	Complement component C6	**5**	300.6	**3**	210.8
NID1_HUMAN	Nidogen-1	**5**	314.1	**3**	190.7
NEP_HUMAN	Neprilysin	**5**	226.1	**5**	273.3
CATC_HUMAN	Dipeptidyl peptidase 1	**5**	339.4	**2**	157.8
PGRP2_HUMAN	N-acetylmuramoyl-L-alanine amidase	**6**	323.8	**2**	171.7
KV301_HUMAN	Ig kappa chain V-III region B6	**2**	160.1	**4**	330.2
CO9_HUMAN	Complement component C9	**6**	312.3	**3**	176.5
UFO_HUMAN	Tyrosine-protein kinase receptor UFO	**6**	236.4	**4**	248.3
IBP7_HUMAN	Insulin-like growth factor-binding protein 7	**5**	229.3	**6**	255.4
ROBO4_HUMAN	Roundabout homolog 4	**4**	225.7	**4**	258.5
PCOC1_HUMAN	Procollagen C-endopeptidase enhancer 1	**4**	197.5	**4**	279.6
CO5_HUMAN	Complement C5	**4**	201.1	**4**	263.4
G3P_HUMAN	Glyceraldehyde-3-phosphate dehydrogenase	**8**	358.6	**2**	105.6
CATA_HUMAN	Catalase	**5**	262.8	**3**	197.7
CEL_HUMAN	Bile salt-activated lipase	**5**	261.3	**3**	197.3
1433Z_HUMAN	14-3-3 protein zeta/delta	**3**	182.5	**3**	274.6
DIAC_HUMAN	Di-N-acetylchitobiase	**4**	215.1	**4**	240.1
GOLM1_HUMAN	Golgi membrane protein 1	**5**	242.9	**4**	205.5
HV320_HUMAN	Ig heavy chain V-III region GAL	**4**	230	**3**	216.5
KV121_HUMAN	Ig kappa chain V-I region Ni	**2**	194.5	**2**	242.9
PZP_HUMAN	Pregnancy zone protein	**7**	236.1	**4**	195.7
LUM_HUMAN	Lumican	**6**	229.2	**4**	202.5
CBG_HUMAN	Corticosteroid-binding globulin	**4**	227.3	**4**	199.8
PSCA_HUMAN	Prostate stem cell antigen	**3**	227.7	**3**	197.9
NTRI_HUMAN	Neurotrimin	**1**	77.2	**3**	343.4
NGAL_HUMAN	Neutrophil gelatinase-associated lipocalin	**4**	177.7	**4**	241.2

Table S3. *Cont.*

Accession	Protein Name	FT1 #Peptides	FT1 Score	FT2 #Peptides	FT2 Score
KV204_HUMAN	Ig kappa chain V-II region TEW	**3**	173.2	**3**	241.6
BCAM_HUMAN	Basal cell adhesion molecule	**5**	210.1	**3**	203.8
CATB_HUMAN	Cathepsin B	**4**	206	**3**	206.4
SUSD2_HUMAN	Sushi domain-containing protein 2	**4**	339.2	**1**	68.2
PPGB_HUMAN	Lysosomal protective protein	**3**	209.8	**3**	197.6
ANXA1_HUMAN	Annexin A1	**1**	67.4	**6**	337.8
MUC18_HUMAN	Cell surface glycoprotein MUC18	**5**	242.2	**3**	162.5
C4BPA_HUMAN	C4b-binding protein alpha chain	**6**	265.5	**3**	132.2
CATH_HUMAN	Cathepsin H	**4**	217	**3**	178.4
CD248_HUMAN	Endosialin	**3**	195.2	**5**	200
LDHB_HUMAN	L-lactate dehydrogenase B chain	**4**	214.9	**3**	177.9
BHMT1_HUMAN	Betaine--homocysteine S-methyltransferase 1	**4**	230.2	**3**	160.6
CO8A_HUMAN	Complement component C8 alpha chain	**5**	236.2	**2**	148.2
CALM_HUMAN	Calmodulin	**3**	180	**3**	204.2
MXRA8_HUMAN	Matrix-remodeling-associated protein 8	**4**	183.2	**3**	200.4
TNR16_HUMAN	Tumor necrosis factor receptor superfamily member 16	**2**	180	**3**	203
HS90A_HUMAN	Heat shock protein HSP 90-alpha	**4**	247.3	**3**	134.3
HV303_HUMAN	Ig heavy chain V-III region VH26	**3**	162.6	**4**	218.1
K2C6C_HUMAN	Keratin, type II cytoskeletal 6C	**5**	221.7	**3**	156.5
ITIH3_HUMAN	Inter-alpha-trypsin inhibitor heavy chain H3	**3**	164.6	**4**	212.7
K1C14_HUMAN	Keratin, type I cytoskeletal 14	**3**	153.1	**2**	224
MMP9_HUMAN	Matrix metalloproteinase-9	**5**	261.6	**2**	114.9
4F2_HUMAN	4F2 cell-surface antigen heavy chain	**3**	176.6	**3**	191.5
ANGT_HUMAN	Angiotensinogen	**3**	184	**4**	183.9
OLFM4_HUMAN	Olfactomedin-4	**4**	179.4	**4**	186.9
ANXA3_HUMAN	Annexin A3	**3**	138.3	**4**	227.1
CATZ_HUMAN	Cathepsin Z	**5**	216.1	**2**	149.1
C1RL_HUMAN	Complement C1r subcomponent-like protein	**2**	148.7	**3**	214.8
BTD_HUMAN	Biotinidase	**2**	117.4	**4**	244.7
CSF1_HUMAN	Macrophage colony-stimulating factor 1	**3**	178	**4**	183.4
PGK1_HUMAN	Phosphoglycerate kinase 1	**2**	106.5	**4**	254.3
CO8G_HUMAN	Complement component C8 gamma chain	**2**	160.6	**3**	195.6
PCD24_HUMAN	Protocadherin-24	**4**	173	**3**	178.2
IGJ_HUMAN	Immunoglobulin J chain	**3**	165.8	**3**	180.4
HS90B_HUMAN	Heat shock protein HSP 90-beta	**4**	243.6	**2**	102.4
MOES_HUMAN	Moesin	**4**	116.4	**3**	228.3
ALDOA_HUMAN	Fructose-bisphosphate aldolase A	**5**	198.7	**2**	138.5

Table S3. *Cont.*

Accession	Protein Name	FT1 #Peptides	FT1 Score	FT2 #Peptides	FT2 Score
WFDC2_HUMAN	WAP four-disulfide core domain protein 2	**2**	116.7	**3**	219.9
CLM9_HUMAN	CMRF35-like molecule 9	**3**	175.4	**2**	155.4
S10A8_HUMAN	Protein S100-A8	**2**	133.2	**3**	196.8
HEP2_HUMAN	Heparin cofactor 2	**4**	231.1	**2**	95.1
CALR_HUMAN	Calreticulin	**3**	163.6	**2**	152.4
AMPE_HUMAN	Glutamyl aminopeptidase	**5**	264.5	**1**	50.3
COF1_HUMAN	Cofilin-1	**2**	146.1	**2**	168.6
CNTN1_HUMAN	Contactin-1	**4**	178.8	**3**	135.7
NAPSA_HUMAN	Napsin-A	**3**	190.6	**2**	123.1
ICOSL_HUMAN	ICOS ligand	**2**	128.8	**2**	184.2
TSN1_HUMAN	Tetraspanin-1	**2**	144.8	**2**	166.7
CD44_HUMAN	CD44 antigen	**2**	107.6	**3**	203.3
PVRL4_HUMAN	Poliovirus receptor-related protein 4	**3**	205.1	**1**	105.6
GGH_HUMAN	Gamma-glutamyl hydrolase	**4**	193.3	**2**	117.1
FBLN4_HUMAN	EGF-containing fibulin-like extracellular matrix protein 2	**2**	164	**2**	145.5
HV304_HUMAN	Ig heavy chain V-III region TIL	**2**	144.6	**2**	160.4
THY1_HUMAN	Thy-1 membrane glycoprotein	**2**	101.6	**3**	201
FAM3C_HUMAN	Protein FAM3C	**2**	98.8	**4**	200.6
S12A1_HUMAN	Solute carrier family 12 member 1	**3**	131	**3**	167.2
PODXL_HUMAN	Podocalyxin-like protein 1	**3**	150.7	**2**	144.7
UROK_HUMAN	Urokinase-type plasminogen activator	**3**	126	**3**	167.7
LAIR1_HUMAN	Leukocyte-associated immunoglobulin-like receptor 1	**2**	126.3	**2**	163.1
QSOX1_HUMAN	Sulfhydryl oxidase 1	**2**	110.5	**4**	178.1
RNAS1_HUMAN	Ribonuclease pancreatic	**1**	28.9	**4**	259
COFA1_HUMAN	Collagen alpha-1(XV) chain	**3**	171.7	**2**	115.9
MUCDL_HUMAN	Mucin and cadherin-like protein	**2**	156.9	**2**	129.4
FBN1_HUMAN	Fibrillin-1	**5**	231.5	**2**	54.5
PDIA1_HUMAN	Protein disulfide-isomerase	**3**	183.6	**2**	100.5
ML12B_HUMAN	Myosin regulatory light chain 12B	**2**	160.4	**2**	123.3
RNAS2_HUMAN	Non-secretory ribonuclease	**3**	90.8	**3**	192
EPHB4_HUMAN	Ephrin type-B receptor 4	**2**	128.6	**2**	148.8
TTHY_HUMAN	Transthyretin	**3**	148.8	**2**	126.6
F16P1_HUMAN	Fructose-1,6-bisphosphatase 1	**3**	138.4	**2**	134.9
SCTM1_HUMAN	Secreted and transmembrane protein 1	**2**	124.9	**2**	148.4
PROZ_HUMAN	Vitamin K-dependent protein Z	**2**	121.2	**2**	150.9
PEBP4_HUMAN	Phosphatidylethanolamine-binding protein 4	**1**	35.2	**3**	231.8
PVR_HUMAN	Poliovirus receptor	**1**	47.4	**4**	219.2

Table S3. *Cont.*

Accession	Protein Name	FT1 #Peptides	FT1 Score	FT2 #Peptides	FT2 Score
TPIS_HUMAN	Triosephosphate isomerase	**4**	173.1	**2**	93.4
SAP_HUMAN	Proactivator polypeptide	**2**	127.7	**2**	138.7
KV312_HUMAN	Ig kappa chain V-III region HAH	**2**	93	**2**	172.9
FBLN2_HUMAN	Fibulin-2	**2**	156.2	**2**	108.2
KV203_HUMAN	Ig kappa chain V-II region MIL	**2**	120.4	**2**	143.6
LAMC1_HUMAN	Laminin subunit gamma-1	**3**	137.8	**2**	124.5
MARCS_HUMAN	Myristoylated alanine-rich C-kinase substrate	**3**	121.3	**2**	140.3
C1R_HUMAN	Complement C1r subcomponent	**2**	117.1	**2**	144
SODE_HUMAN	Extracellular superoxide dismutase [Cu-Zn]	**3**	165.8	**2**	94
CBPM_HUMAN	Carboxypeptidase M	**4**	190.6	**2**	67
R4RL2_HUMAN	Reticulon-4 receptor-like 2	**2**	107.3	**2**	149.6
COMP_HUMAN	Cartilage oligomeric matrix protein	**3**	188	**1**	66.9
NUCB1_HUMAN	Nucleobindin-1	**3**	161.3	**1**	91.6
CD81_HUMAN	CD81 antigen	**3**	114	**2**	138.8
CO2_HUMAN	Complement C2	**2**	89	**2**	162.4
BT2A2_HUMAN	Butyrophilin subfamily 2 member A2	**2**	116.8	**2**	134.4
MUC1_HUMAN	Mucin-1	**2**	90.1	**3**	158.5
PRDX6_HUMAN	Peroxiredoxin-6	**4**	189.9	**1**	58.6
CRAC1_HUMAN	Cartilage acidic protein 1	**2**	105.8	**3**	140.8
KCRB_HUMAN	Creatine kinase B-type	**3**	133.5	**1**	110.3
LAMP2_HUMAN	Lysosome-associated membrane glycoprotein 2	**2**	133.4	**2**	109.9
PRIO_HUMAN	Major prion protein	**2**	111.8	**2**	127.4
MA2B2_HUMAN	Epididymis-specific alpha-mannosidase	**2**	75	**3**	161.7
PDC6I_HUMAN	Programmed cell death 6-interacting protein	**2**	106.7	**2**	129.5
OPCM_HUMAN	Opioid-binding protein/cell adhesion molecule	**1**	60.1	**3**	175.1
CADM1_HUMAN	Cell adhesion molecule 1	**3**	126.4	**2**	107.5
DDR1_HUMAN	Epithelial discoidin domain-containing receptor 1	**2**	121.3	**2**	112.4
ANXA4_HUMAN	Annexin A4	**2**	111.5	**2**	121.6
SIAE_HUMAN	Sialate O=-acetylesterase	**2**	152.2	**1**	79.3
B2MG_HUMAN	Beta-2-microglobulin	**2**	89.1	**3**	142.2
FHR1_HUMAN	Complement factor H-related protein 1	**4**	139.6	**2**	90.9
UBIQ_HUMAN	Ubiquitin	**3**	169.3	**1**	61.1
GSTM3_HUMAN	Glutathione S-transferase Mu 3	**2**	99.8	**2**	130.6
EPHB2_HUMAN	Ephrin type-B receptor 2	**2**	140	**1**	86.3
LFA3_HUMAN	Lymphocyte function-associated antigen 3	**2**	91.5	**2**	132.5

Table S3. *Cont.*

Accession	Protein Name	FT1 #Peptides	FT1 Score	FT2 #Peptides	FT2 Score
CSTN1_HUMAN	Calsyntenin-1	**2**	125.3	**2**	96.3
HEXB_HUMAN	Beta-hexosaminidase subunit beta	**3**	159.2	**1**	62
TRBM_HUMAN	Thrombomodulin	**3**	145	**1**	75.4
GPC5C_HUMAN	G-protein coupled receptor family C group 5 member C	**2**	81	**2**	137.5
LYVE1_HUMAN	Lymphatic vessel endothelial hyaluronic acid receptor 1	**2**	82.4	**3**	135
NEO1_HUMAN	Neogenin	**3**	110.5	**2**	106
CO8B_HUMAN	Complement component C8 beta chain	**2**	77.6	**3**	137.8
ANGL2_HUMAN	Angiopoietin-related protein 2	**2**	79.7	**3**	134.2
EPHA4_HUMAN	Ephrin type-A receptor 4	**2**	112.5	**1**	100
HGFA_HUMAN	Hepatocyte growth factor activator	**3**	149.7	**1**	62.4
CALL3_HUMAN	Calmodulin-like protein 3	**2**	108.8	**2**	103.1
CPVL_HUMAN	Probable serine carboxypeptidase CPVL	**2**	93.8	**3**	117.4
GRP78_HUMAN	78 kDa glucose-regulated protein	**4**	139.2	**2**	71
OLR1_HUMAN	Oxidized low-density lipoprotein receptor 1	**2**	82.3	**2**	127.7
CD59_HUMAN	CD59 glycoprotein	**2**	74.6	**3**	126.5
TRFM_HUMAN	Melanotransferrin	**3**	136.4	**1**	63.9
RNF13_HUMAN	RING finger protein 13	**2**	110.7	**1**	86.3
GNAI3_HUMAN	Guanine nucleotide-binding protein G(k) subunit alpha	**1**	150.7	**1**	45.9
LCAT_HUMAN	Phosphatidylcholine-sterol acyltransferase	**2**	98.9	**2**	96.3
CBPB2_HUMAN	Carboxypeptidase B2	**3**	165.8	**1**	27.6
IQGA1_HUMAN	Ras GTPase-activating-like protein IQGAP1	**3**	111.3	**1**	81.2
C1QB_HUMAN	Complement C1q subcomponent subunit B	**1**	94.9	**1**	95.2
DPP2_HUMAN	Dipeptidyl peptidase 2	**2**	102.4	**1**	86.4
CBPE_HUMAN	Carboxypeptidase E	**1**	45.8	**3**	142.3
HEXA_HUMAN	Beta-hexosaminidase subunit alpha	**2**	85.6	**2**	101.3
LV301_HUMAN	Ig lambda chain V-III region SH	**1**	84.7	**1**	101.3
8ODP_HUMAN	7,8-dihydro-8-oxoguanine triphosphatase	**1**	67	**2**	118.3
GPVI_HUMAN	Platelet glycoprotein VI	**1**	59.4	**3**	125.5
ROR1_HUMAN	Tyrosine-protein kinase transmembrane receptor ROR1	**1**	55.3	**1**	128.7
GP1BA_HUMAN	Platelet glycoprotein Ib alpha chain	**1**	52.6	**3**	131.2
CRIS3_HUMAN	Cysteine-rich secretory protein 3	**1**	103.2	**2**	79
ZG16B_HUMAN	Zymogen granule protein 16 homolog B	**1**	70.3	**1**	110.8
ANXA5_HUMAN	Annexin A5	**2**	76.6	**2**	103.9
EF1A1_HUMAN	Elongation factor 1-alpha 1	**2**	81	**2**	98.4

Table S3. *Cont.*

Accession	Protein Name	FT1 #Peptides	FT1 Score	FT2 #Peptides	FT2 Score
CLM8_HUMAN	CMRF35-like molecule 8	**1**	55.3	**1**	121.3
JAM3_HUMAN	Junctional adhesion molecule C	**2**	91.6	**1**	84.1
OSCAR_HUMAN	Osteoclast-associated immunoglobulin-like receptor	**1**	69.2	**2**	100.9
A4_HUMAN	Amyloid beta A4 protein	**1**	77.9	**1**	92.1
HV301_HUMAN	Ig heavy chain V-III region TRO	**1**	84.8	**1**	85.2
DPEP1_HUMAN	Dipeptidase 1	**1**	53.4	**3**	114.1
GFRP_HUMAN	GTP cyclohydrolase 1 feedback regulatory protein	**2**	101.3	**1**	66.2
1433T_HUMAN	14-3-3 protein theta	**1**	62.9	**1**	103.6
LV302_HUMAN	Ig lambda chain V-III region LOI	**2**	88.4	**1**	76.4
GGT6_HUMAN	Gamma-glutamyltransferase 6	**2**	86.2	**1**	75.6
DPP4_HUMAN	Dipeptidyl peptidase 4	**1**	75.2	**2**	84.8
6PGL_HUMAN	6-phosphogluconolactonase	**2**	84.5	**2**	75.3
FCGBP_HUMAN	IgGFc-binding protein	**3**	113.7	**1**	45
HV302_HUMAN	Ig heavy chain V-III region WEA	**1**	54.4	**2**	103.2
TFF2_HUMAN	Trefoil factor 2	**1**	53.2	**2**	102.1
S10AB_HUMAN	Protein S100-A11	**1**	55	**2**	100.3
MDR1_HUMAN	Multidrug resistance protein 1	**2**	89.7	**2**	64.3
CNTFR_HUMAN	Ciliary neurotrophic factor receptor subunit alpha	**2**	76.7	**1**	75.7
RISC_HUMAN	Retinoid-inducible serine carboxypeptidase	**2**	114	**1**	35.9
LAYN_HUMAN	Layilin	**1**	75.1	**1**	73.3
MUC5B_HUMAN	Mucin-5B	**2**	101.3	**1**	44
MSTP9_HUMAN	Putative macrophage-stimulating protein MSTP9	**1**	72	**1**	72.9
GDIR1_HUMAN	Rho GDP-dissociation inhibitor 1	**2**	72.6	**1**	70.6
IDHC_HUMAN	Isocitrate dehydrogenase [NADP] cytoplasmic	**2**	69.8	**1**	72.7
INAR2_HUMAN	Interferon alpha/beta receptor 2	**1**	67.5	**1**	74.8
CAH2_HUMAN	Carbonic anhydrase 2	**1**	38	**2**	102.8
MADCA_HUMAN	Mucosal addressin cell adhesion molecule 1	**1**	28.1	**1**	110.1
PRTN3_HUMAN	Myeloblastin	**2**	110	**1**	26.1
1433E_HUMAN	14-3-3 protein epsilon	**1**	48.5	**2**	87.5
ROR2_HUMAN	Tyrosine-protein kinase transmembrane receptor ROR2	**1**	64.2	**1**	71.5
PGM1_HUMAN	Phosphoglucomutase-1	**1**	49.8	**2**	85.8
CO5A3_HUMAN	Collagen alpha-3(V) chain	**2**	70.6	**1**	64
ASAH1_HUMAN	Acid ceramidase	**2**	61	**2**	71.6

Table S3. *Cont.*

Accession	Protein Name	FT1 #Peptides	FT1 Score	FT2 #Peptides	FT2 Score
NEGR1_HUMAN	Neuronal growth regulator 1	**2**	93.9	**1**	37.6
DHSO_HUMAN	Sorbitol dehydrogenase	**2**	63.7	**1**	67.4
PCP_HUMAN	Lysosomal Pro-X carboxypeptidase	**1**	28.4	**2**	102.3
SDK1_HUMAN	Protein sidekick-1	**1**	46.8	**1**	83.5
CLC14_HUMAN	C-type lectin domain family 14 member A	**2**	80.9	**1**	49.2
SKP1_HUMAN	S-phase kinase-associated protein 1	**1**	59.1	**1**	71
TENX_HUMAN	Tenascin-X	**1**	63.8	**1**	65.9
CYTM_HUMAN	Cystatin-M	**2**	88.2	**1**	39
C1QC_HUMAN	Complement C1q subcomponent subunit C	**1**	25.7	**2**	100.5
LYSC_HUMAN	Lysozyme C	**2**	85	**1**	40
PDZ1P_HUMAN	Putative PDZ domain-containing protein 1P	**1**	57.6	**1**	67.2
CADH6_HUMAN	Cadherin-6	**1**	65	**1**	59
ARF3_HUMAN	ADP-ribosylation factor 3	**1**	48.4	**1**	75.4
PHLD_HUMAN	Phosphatidylinositol-glycan-specific phospholipase D	**1**	49.6	**1**	71.1
GPC5B_HUMAN	G-protein coupled receptor family C group 5 member B	**1**	43.5	**2**	76.7
APOA2_HUMAN	Apolipoprotein A-II	**1**	66.4	**1**	53.8
CAPG_HUMAN	Macrophage-capping protein	**2**	68.3	**1**	51.9
PPAL_HUMAN	Lysosomal acid phosphatase	**1**	32	**2**	85.6
PTPRG_HUMAN	Receptor-type tyrosine-protein phosphatase gamma	**1**	38.7	**2**	78.7
CHM2A_HUMAN	Charged multivesicular body protein 2a	**1**	26.2	**1**	91.1
PROS_HUMAN	Vitamin K-dependent protein S	**2**	66.8	**1**	50.4
RINI_HUMAN	Ribonuclease inhibitor	**1**	73.5	**1**	43.4
SC5A1_HUMAN	Sodium/glucose cotransporter 1	**1**	36	**1**	80.4
EZRI_HUMAN	Ezrin	**2**	61.9	**1**	54.4
K1C19_HUMAN	Keratin, type I cytoskeletal 19	**2**	73.3	**1**	42.3
LV403_HUMAN	Ig lambda chain V-IV region Hil	**1**	30.6	**2**	82.9
PVRL2_HUMAN	Poliovirus receptor-related protein 2	**1**	54.9	**1**	58.5
IST1_HUMAN	IST1 homolog	**1**	59.4	**1**	53.6
GGT1_HUMAN	Gamma-glutamyltranspeptidase 1	**1**	38.6	**2**	73.8
DKK3_HUMAN	Dickkopf-related protein 3	**1**	68.3	**1**	44.1
CHL1_HUMAN	Neural cell adhesion molecule L1-like protein	**1**	45.9	**2**	66.1
SE6L2_HUMAN	Seizure 6-like protein 2	**1**	32.7	**2**	79.1
CPN2_HUMAN	Carboxypeptidase N subunit 2	**2**	71.7	**1**	39.9
VSIG4_HUMAN	V-set and immunoglobulin domain-containing protein 4	**1**	62.2	**1**	48.9

Table S3. *Cont.*

Accession	Protein Name	FT1 #Peptides	FT1 Score	FT2 #Peptides	FT2 Score
KAIN_HUMAN	Kallistatin	**1**	42.5	**2**	67.8
COIA1_HUMAN	Collagen alpha-1(XVIII) chain	**2**	70	**1**	40.3
TGM4_HUMAN	Protein-glutamine gamma-glutamyltransferase 4	**2**	79.3	**1**	30.9
TWSG1_HUMAN	Twisted gastrulation protein homolog 1	**1**	41.1	**1**	69
TNR8_HUMAN	Tumor necrosis factor receptor superfamily member 8	**1**	69.2	**1**	40
HV310_HUMAN	Ig heavy chain V-III region HIL	**1**	51.3	**1**	57.4
ITB1_HUMAN	Integrin beta-1	**2**	63.5	**1**	44.6
CTL4_HUMAN	Choline transporter-like protein 4	**1**	36.6	**1**	71.2
GDIB_HUMAN	Rab GDP dissociation inhibitor beta	**1**	46.3	**1**	60.5
MA2B1_HUMAN	Lysosomal alpha-mannosidase	**1**	40.6	**1**	63.8
SFRP4_HUMAN	Secreted frizzled-related protein 4	**1**	62.4	**1**	41.2
L1CAM_HUMAN	Neural cell adhesion molecule L1	**1**	53.1	**1**	49.6
SH3L3_HUMAN	SH3 domain-binding glutamic acid-rich-like protein 3	**1**	31.1	**1**	71.2
ELNE_HUMAN	Neutrophil elastase	**2**	68.7	**1**	33.1
APOL1_HUMAN	Apolipoprotein L1	**1**	31	**1**	70.8
KLKB1_HUMAN	Plasma kallikrein	**1**	28.8	**1**	72.9
MSLN_HUMAN	Mesothelin	**1**	34.9	**2**	66.7
NHRF1_HUMAN	Na(+)/H(+) exchange regulatory cofactor NHE-RF1	**1**	51.7	**1**	49.7
EFNB1_HUMAN	Ephrin-B1	**2**	63.1	**1**	37.2
RAP1B_HUMAN	Ras-related protein Rap-1b	**1**	67.5	**1**	30.5
PEPA_HUMAN	Pepsin A	**1**	51	**1**	46
CHMP5_HUMAN	Charged multivesicular body protein 5	**1**	25.7	**2**	70.8
ICAM2_HUMAN	Intercellular adhesion molecule 2	**1**	59.2	**1**	36.6
FABPH_HUMAN	Fatty acid-binding protein, heart	**1**	43.1	**1**	52.5
TKT_HUMAN	Transketolase	**1**	46.8	**1**	48.5
TINAL_HUMAN	Tubulointerstitial nephritis antigen-like	**1**	48.1	**1**	45.5
PCDH1_HUMAN	Protocadherin-1	**1**	50.4	**1**	42.5
EPHB3_HUMAN	Ephrin type-B receptor 3	**1**	25.3	**1**	67.5
RCN1_HUMAN	Reticulocalbin-1	**1**	52.7	**1**	39.5
NPC2_HUMAN	Epididymal secretory protein E1	**1**	37.8	**1**	54.2
HV102_HUMAN	Ig heavy chain V-I region HG3	**1**	36.9	**1**	54.8
A2AP_HUMAN	Alpha-2-antiplasmin	**2**	55.6	**1**	35.8
GGCT_HUMAN	Gamma-glutamylcyclotransferase	**1**	27.5	**2**	62.9
A2LD1_HUMAN	AIG2-like domain-containing protein 1	**1**	41.6	**1**	48.2
MMRN1_HUMAN	Multimerin-1	**1**	41.1	**1**	48.5
S10AE_HUMAN	Protein S100-A14	**1**	34.7	**1**	54.4

Table S3. *Cont.*

Accession	Protein Name	FT1 #Peptides	FT1 Score	FT2 #Peptides	FT2 Score
ARP2_HUMAN	Actin-related protein 2	**1**	51.2	**1**	36.7
FILA2_HUMAN	Filaggrin-2	**1**	37.7	**1**	50.2
GDIA_HUMAN	Rab GDP dissociation inhibitor alpha	**1**	40.9	**1**	44.7
MANBA_HUMAN	Beta-mannosidase	**1**	50.7	**1**	34
IL2RA_HUMAN	Interleukin-2 receptor subunit alpha	**1**	52	**1**	31.8
ISLR_HUMAN	Immunoglobulin superfamily containing leucine-rich repeat protein	**1**	30.3	**1**	53.2
ABCBB_HUMAN	Bile salt export pump	**1**	40.5	**1**	42.6
PEAR1_HUMAN	Platelet endothelial aggregation receptor 1	**1**	32.4	**1**	50.1
ACE_HUMAN	Angiotensin-converting enzyme	**1**	35.2	**1**	45.6
CAZA1_HUMAN	F-actin-capping protein subunit alpha-1	**1**	32.7	**1**	47.9
MUC5A_HUMAN	Mucin-5AC (Fragments)	**1**	54	**1**	26.4
CBPZ_HUMAN	Carboxypeptidase Z	**1**	47.3	**1**	31.8
LAMP1_HUMAN	Lysosome-associated membrane glycoprotein 1	**1**	28.4	**1**	48.5
AT1A1_HUMAN	Sodium/potassium-transporting ATPase subunit alpha-1	**1**	44.5	**1**	32.2
LGMN_HUMAN	Legumain	**1**	35.6	**1**	40.3
MVP_HUMAN	Major vault protein	**1**	34	**1**	41.5
SYUG_HUMAN	Gamma-synuclein	**1**	31.8	**1**	43.7
LBP_HUMAN	Lipopolysaccharide-binding protein	**1**	36.5	**1**	37.7
CD27_HUMAN	CD27 antigen	**1**	38.3	**1**	35.2
TIMD3_HUMAN	Hepatitis A virus cellular receptor 2	**1**	36.2	**1**	36.8
SDC1_HUMAN	Syndecan-1	**1**	31.6	**1**	39.1
BASI_HUMAN	Basigin	**1**	29.2	**1**	40.4
STK24_HUMAN	Serine/threonine-protein kinase 24	**1**	40.8	**1**	28.6
PGCB_HUMAN	Brevican core protein	**1**	29.6	**1**	39.3
PGAM1_HUMAN	Phosphoglycerate mutase 1	**1**	39.4	**1**	28.7
MUC20_HUMAN	Mucin-20	**1**	29.3	**1**	38.5
CD7_HUMAN	T-cell antigen CD7	**1**	28.4	**1**	39.3
CP089_HUMAN	Uncharacterized protein C16orf89	**1**	34.2	**1**	32.7
LPHN2_HUMAN	Latrophilin-2	**1**	34.4	**1**	31.9
AB12B_HUMAN	Abhydrolase domain-containing protein 12B	**1**	33.6	**1**	32
PRG2_HUMAN	Bone marrow proteoglycan	**1**	38.3	**1**	26.2
CASPE_HUMAN	Caspase-14	**1**	37.6	**1**	26.6
KV310_HUMAN	Ig kappa chain V-III region VH (Fragment)	**1**	32.5	**1**	31.6
RN214_HUMAN	RING finger protein 214	**1**	31.9	**1**	31.9
TM7S3_HUMAN	Transmembrane 7 superfamily member 3	**1**	25.5	**1**	36.3
FUCO2_HUMAN	Plasma alpha-L-fucosidase	**1**	32.4	**1**	27.6

Table S4. Urinary proteins enriched by AAL (>2-fold between eluate and flow through). The AAL-FT column is the total number of AAL peptides minus the total number of FT peptides identified. The fold change column is the total number of AAL peptides divided by the total number of FT peptides. Where the value is N/A it indicates that there were no peptide identifications in the FT so the calculation is not possible.

**Accession**	**Protein Name**	**AAL1. #Peptides**	**AAL1. Scores**	**AAL2. #Peptides**	**AAL2. Scores**	**AAL-FT**	**Fold Change**
GSLG1_HUMAN	Golgi apparatus protein 1	**9**	532.0 (M:532.0)	**10**	549.2 (M:549.2)	19	N/A
FREM2_HUMAN	FRAS1-related extracellular matrix protein 2	**7**	382.5 (M:382.5)	**7**	314.6 (M:314.6)	14	N/A
MEGF8_HUMAN	Multiple epidermal growth factor-like domains protein 8	**6**	348.5 (M:348.5)	**7**	434.0 (M:434.0)	13	N/A
VWF_HUMAN	von Willebrand factor	**3**	115.6 (M:115.6)	**7**	268.0 (M:268.0)	10	N/A
ABP1_HUMAN	Amiloride-sensitive amine oxidase [copper-containing]	**3**	187.9 (M:187.9)	**6**	308.1 (M:308.1)	9	N/A
HV307_HUMAN	Ig heavy chain V-III region CAM	**4**	176.5 (M:176.5)	**4**	202.5 (M:202.5)	8	N/A
FAT2_HUMAN	Protocadherin Fat 2	**3**	136.3 (M:136.3)	**5**	236.7 (M:236.7)	8	N/A
TSP1_HUMAN	Thrombospondin-1	**3**	185.1 (M:185.1)	**5**	327.7 (M:327.7)	8	N/A
TRHDE_HUMAN	Thyrotropin-releasing hormone-degrading ectoenzyme	**3**	112.6 (M:112.6)	**5**	215.0 (M:215.0)	8	N/A
FTHFD_HUMAN	10-formyltetrahydrofolate dehydrogenase	**3**	186.8 (M:186.8)	**3**	150.1 (M:150.1)	6	N/A
GAS6_HUMAN	Growth arrest-specific protein 6	**3**	139.8 (M:139.8)	**3**	224.9 (M:224.9)	6	N/A
ALS_HUMAN	Insulin-like growth factor-binding protein complex acid labile subunit	**3**	161.5 (M:161.5)	**3**	126.3 (M:126.3)	6	N/A
LRC19_HUMAN	Leucine-rich repeat-containing protein 19	**3**	192.9 (M:192.9)	**3**	233.6 (M:233.6)	6	N/A
SAHH_HUMAN	Adenosylhomocysteinase	**3**	116.1 (M:116.1)	**2**	77.5 (M:77.5)	5	N/A
CBR1_HUMAN	Carbonyl reductase [NADPH] 1	**2**	64.4 (M:64.4)	**3**	159.5 (M:159.5)	5	N/A
CNDP2_HUMAN	Cytosolic non-specific dipeptidase	**3**	147.5 (M:147.5)	**2**	66.9 (M:66.9)	5	N/A
DMBT1_HUMAN	Deleted in malignant brain tumors 1 protein	**3**	208.2 (M:208.2)	**2**	157.7 (M:157.7)	5	N/A
FGL2_HUMAN	Fibroleukin	**2**	52.2 (M:52.2)	**3**	132.8 (M:132.8)	5	N/A

Table S4. *Cont.*

**Accession**	**Protein Name**	**AAL1. #Peptides**	**AAL1. Scores**	**AAL2. #Peptides**	**AAL2. Scores**	**AAL-FT**	**Fold Change**
G6PI_HUMAN	Glucose-6-phosphate isomerase	**3**	150.5 (M:150.5)	**2**	130.9 (M:130.9)	5	N/A
PAG15_HUMAN	Group XV phospholipase A2	**2**	116.0 (M:116.0)	**3**	173.3 (M:173.3)	5	N/A
UBA1_HUMAN	Ubiquitin-like modifier-activating enzyme 1	**2**	105.4 (M:105.4)	**3**	145.7 (M:145.7)	5	N/A
AATC_HUMAN	Aspartate aminotransferase, cytoplasmic	**2**	58.3 (M:58.3)	**2**	70.4 (M:70.4)	4	N/A
CAMP_HUMAN	Cathelicidin antimicrobial peptide	**2**	75.9 (M:75.9)	**2**	75.7 (M:75.7)	4	N/A
GLCM_HUMAN	Glucosylceramidase	**2**	129.7 (M:129.7)	**2**	110.3 (M:110.3)	4	N/A
GP126_HUMAN	G-protein coupled receptor 126	**2**	116.3 (M:116.3)	**2**	123.7 (M:123.7)	4	N/A
HV306_HUMAN	Ig heavy chain V-III region BUT	**1**	101.4 (M:101.4)	**3**	145.0 (M:145.0)	4	N/A
KV105_HUMAN	Ig kappa chain V-I region DEE	**2**	217.0 (M:217.0)	**2**	168.9 (M:168.9)	4	N/A
PLAK_HUMAN	Junction plakoglobin	**2**	122.7 (M:122.7)	**2**	83.6 (M:83.6)	4	N/A
LDHA_HUMAN	L-lactate dehydrogenase A chain	**2**	144.6 (M:144.6)	**2**	134.6 (M:134.6)	4	N/A
FCG3A_HUMAN	Low affinity immunoglobulin gamma Fc region receptor III-A	**2**	91.6 (M:91.6)	**2**	119.3 (M:119.3)	4	N/A
MMRN2_HUMAN	Multimerin-2	**2**	158.5 (M:158.5)	**2**	133.1 (M:133.1)	4	N/A
FAT4_HUMAN	Protocadherin Fat 4	**1**	48.7 (M:48.7)	**3**	142.2 (M:142.2)	4	N/A
PTPRK_HUMAN	Receptor-type tyrosine-protein phosphatase kappa	**3**	149.7 (M:149.7)	**1**	47.3 (M:47.3)	4	N/A
TARSH_HUMAN	Target of Nesh-SH3	**1**	32.1 (M:32.1)	**3**	121.8 (M:121.8)	4	N/A
FUCO_HUMAN	Tissue alpha-L-fucosidase	**2**	109.8 (M:109.8)	**2**	75.8 (M:75.8)	4	N/A
ANPRC_HUMAN	Atrial natriuretic peptide receptor 3	**2**	104.4 (M:104.4)	**1**	33.8 (M:33.8)	3	N/A
B4GT1_HUMAN	Beta-1,4-galactosyltransferase 1	**1**	78.2 (M:78.2)	**2**	143.6 (M:143.6)	3	N/A
DHAK_HUMAN	Bifunctional ATP-dependent dihydroxyacetone kinase/FAD-AMP lyase (cyclizing)	**1**	45.6 (M:45.6)	**2**	101.5 (M:101.5)	3	N/A

Table S4. *Cont.*

Accession	Protein Name	AAL1. #Peptides	AAL1. Scores	AAL2. #Peptides	AAL2. Scores	AAL-FT	Fold Change
CAN1_HUMAN	Calpain-1 catalytic subunit	**1**	43.2 (M:43.2)	**2**	84.6 (M:84.6)	3	N/A
CBLN2_HUMAN	Cerebellin-2	**2**	113.3 (M:113.3)	**1**	35.4 (M:35.4)	3	N/A
GUAD_HUMAN	Guanine deaminase	**2**	81.2 (M:81.2)	**1**	38.9 (M:38.9)	3	N/A
KV104_HUMAN	Ig kappa chain V-I region CAR	**2**	181.5 (M:181.5)	**1**	116.8 (M:116.8)	3	N/A
PCKGC_HUMAN	Phosphoenolpyruvate carboxykinase, cytosolic [GTP]	**1**	52.6 (M:52.6)	**2**	70.7 (M:70.7)	3	N/A
RENR_HUMAN	Renin receptor	**1**	57.0 (M:57.0)	**2**	75.4 (M:75.4)	3	N/A
TIG1_HUMAN	Retinoic acid receptor responder protein 1	**1**	72.0 (M:72.0)	**2**	65.6 (M:65.6)	3	N/A
SERC1_HUMAN	Serine incorporator 1	**1**	46.8 (M:46.8)	**2**	90.9 (M:90.9)	3	N/A
BDH2_HUMAN	3-hydroxybutyrate dehydrogenase type 2	**1**	29.0 (M:29.0)	**1**	40.1 (M:40.1)	2	N/A
AL9A1_HUMAN	4-trimethylaminobutyraldehyde dehydrogenase	**1**	33.1 (M:33.1)	**1**	41.3 (M:41.3)	2	N/A
ARC1B_HUMAN	Actin-related protein 2/3 complex subunit 1B	**1**	29.2 (M:29.2)	**1**	34.3 (M:34.3)	2	N/A
ATPB_HUMAN	ATP synthase subunit beta, mitochondrial	**1**	44.7 (M:44.7)	**1**	40.3 (M:40.3)	2	N/A
BPI_HUMAN	Bactericidal permeability-increasing protein	**1**	60.8 (M:60.8)	**1**	76.8 (M:76.8)	2	N/A
PGS1_HUMAN	Biglycan	**1**	68.3 (M:68.3)	**1**	60.7 (M:60.7)	2	N/A
CADH5_HUMAN	Cadherin-5	**1**	54.2 (M:54.2)	**1**	40.6 (M:40.6)	2	N/A
CATG_HUMAN	Cathepsin G	**1**	27.6 (M:27.6)	**1**	45.1 (M:45.1)	2	N/A
CATO_HUMAN	Cathepsin O	**1**	29.1 (M:29.1)	**1**	31.7 (M:31.7)	2	N/A
CD63_HUMAN	CD63 antigen	**1**	50.0 (M:50.0)	**1**	67.4 (M:67.4)	2	N/A
CD9_HUMAN	CD9 antigen	**1**	61.0 (M:61.0)	**1**	65.3 (M:65.3)	2	N/A
F13A_HUMAN	Coagulation factor XIII A chain	**1**	36.8 (M:36.8)	**1**	46.8 (M:46.8)	2	N/A
CRUM2_HUMAN	Crumbs homolog 2	**1**	47.2 (M:47.2)	**1**	51.4 (M:51.4)	2	N/A

Table S4. *Cont.*

**Accession**	**Protein Name**	**AAL1. #Peptides**	**AAL1. Scores**	**AAL2. #Peptides**	**AAL2. Scores**	**AAL-FT**	**Fold Change**
AMPL_HUMAN	Cytosol aminopeptidase	**1**	68.6 (M:68.6)	**1**	54.2 (M:54.2)	2	N/A
DNER_HUMAN	Delta and Notch-like epidermal growth factor-related receptor	**1**	62.9 (M:62.9)	**1**	62.7 (M:62.7)	2	N/A
EPHB6_HUMAN	Ephrin type-B receptor 6	**1**	32.4 (M:32.4)	**1**	43.6 (M:43.6)	2	N/A
LEG9_HUMAN	Galectin-9	**1**	44.7 (M:44.7)	**1**	69.7 (M:69.7)	2	N/A
IGHD_HUMAN	Ig delta chain C region	**1**	36.2 (M:36.2)	**1**	43.9 (M:43.9)	2	N/A
CPXM2_HUMAN	Inactive carboxypeptidase-like protein X2	**1**	29.9 (M:29.9)	**1**	28.0 (M:28.0)	2	N/A
CRYL1_HUMAN	Lambda-crystallin homolog	**1**	73.7 (M:73.7)	**1**	92.7 (M:92.7)	2	N/A
LAMA5_HUMAN	Laminin subunit alpha-5	**1**	40.4 (M:40.4)	**1**	54.0 (M:54.0)	2	N/A
ILEU_HUMAN	Leukocyte elastase inhibitor	**1**	39.1 (M:39.1)	**1**	40.9 (M:40.9)	2	N/A
LIRA3_HUMAN	Leukocyte immunoglobulin-like receptor subfamily A member 3	**1**	32.0 (M:32.0)	**1**	44.4 (M:44.4)	2	N/A
LIPL_HUMAN	Lipoprotein lipase	**1**	57.3 (M:57.3)	**1**	80.0 (M:80.0)	2	N/A
PPT2_HUMAN	Lysosomal thioesterase PPT2	**1**	30.3 (M:30.3)	**1**	34.7 (M:34.7)	2	N/A
MYO6_HUMAN	Myosin-VI	**1**	48.9 (M:48.9)	**1**	40.3 (M:40.3)	2	N/A
NRP1_HUMAN	Neuropilin-1	**1**	32.8 (M:32.8)	**1**	55.7 (M:55.7)	2	N/A
PTTG_HUMAN	Pituitary tumor-transforming gene 1 protein-interacting protein	**1**	37.3 (M:37.3)	**1**	34.9 (M:34.9)	2	N/A
AMNLS_HUMAN	Protein amnionless	**1**	56.9 (M:56.9)	**1**	52.4 (M:52.4)	2	N/A
F151A_HUMAN	Protein FAM151A	**1**	29.6 (M:29.6)	**1**	46.4 (M:46.4)	2	N/A
JAG1_HUMAN	Protein jagged-1	**1**	54.0 (M:54.0)	**1**	58.4 (M:58.4)	2	N/A
TTYH3_HUMAN	Protein tweety homolog 3	**1**	54.6 (M:54.6)	**1**	50.9 (M:50.9)	2	N/A
PNPH_HUMAN	Purine nucleoside phosphorylase	**1**	39.6 (M:39.6)	**1**	31.5 (M:31.5)	2	N/A

Table S4. *Cont.*

**Accession**	**Protein Name**	**AAL1. #Peptides**	**AAL1. Scores**	**AAL2. #Peptides**	**AAL2. Scores**	**AAL-FT**	**Fold Change**
ESTD_HUMAN	S-formylglutathione hydrolase	**1**	34.7 (M:34.7)	**1**	31.7 (M:31.7)	2	N/A
SORT_HUMAN	Sortilin	**1**	28.4 (M:28.4)	**1**	47.4 (M:47.4)	2	N/A
SYPL1_HUMAN	Synaptophysin-like protein 1	**1**	62.2 (M:62.2)	**1**	62.4 (M:62.4)	2	N/A
TRY1_HUMAN	Trypsin-1	**1**	28.7 (M:28.7)	**1**	32.3 (M:32.3)	2	N/A
TACD2_HUMAN	Tumor-associated calcium signal transducer 2	**1**	36.1 (M:36.1)	**1**	26.4 (M:26.4)	2	N/A
WDR1_HUMAN	WD repeat-containing protein 1	**1**	35.2 (M:35.2)	**1**	29.0 (M:29.0)	2	N/A
SORL_HUMAN	Sortilin-related receptor	**14**	842.6 (M:842.6)	**17**	794.7 (M:794.7)	30	31.0
FCGBP_HUMAN	IgGFc-binding protein	**47**	3049.8 (M:3049.8)	**56**	3631.9 (M:3631.9)	99	25.8
MMP8_HUMAN	Neutrophil collagenase	**7**	371.8 (M:371.8)	**6**	431.8 (M:431.8)	12	13.0
LRP2_HUMAN	Low-density lipoprotein receptor-related protein 2	**94**	6243.0 (M:6243.0)	**96**	6360.7 (M:6360.7)	175	12.7
DPP4_HUMAN	Dipeptidyl peptidase 4	**18**	1036.7 (M:1036.7)	**19**	1026.2 (M:1026.2)	34	12.3
ABHEB_HUMAN	Abhydrolase domain-containing protein 14B	**6**	288.8 (M:288.8)	**4**	255.6 (M:255.6)	9	10.0
PTPRJ_HUMAN	Receptor-type tyrosine-protein phosphatase eta	**4**	235.0 (M:235.0)	**6**	270.9 (M:270.9)	9	10.0
SPHM_HUMAN	N-sulphoglucosamine sulphohydrolase	**4**	208.9 (M:208.9)	**5**	269.2 (M:269.2)	8	9.0
ACE2_HUMAN	Angiotensin-converting enzyme 2	**2**	119.0 (M:119.0)	**6**	296.9 (M:296.9)	7	8.0
MA1A1_HUMAN	Mannosyl-oligosaccharide 1,2-alpha-mannosidase IA	**3**	155.9 (M:155.9)	**5**	215.1 (M:215.1)	7	8.0
SPB3_HUMAN	Serpin B3	**3**	174.8 (M:174.8)	**4**	176.3 (M:176.3)	6	7.0
CUBN_HUMAN	Cubilin	**76**	5130.5 (M:5130.5)	**76**	5196.6 (M:5196.6)	129	6.6
MUC5B_HUMAN	Mucin-5B	**9**	422.6 (M:422.6)	**9**	429.0 (M:429.0)	15	6.0
LKHA4_HUMAN	Leukotriene A-4 hydrolase	**3**	100.1 (M:100.1)	**3**	117.8 (M:117.8)	5	6.0
GGT1_HUMAN	Gamma-glutamyltranspeptidase 1	**8**	538.1 (M:538.1)	**9**	565.2 (M:565.2)	14	5.7

Table S4. *Cont.*

**Accession**	**Protein Name**	**AAL1. #Peptides**	**AAL1. Scores**	**AAL2. #Peptides**	**AAL2. Scores**	**AAL-FT**	**Fold Change**
ACY1_HUMAN	Aminoacylase-1	**4**	177.7 (M:177.7)	**7**	340.7 (M:340.7)	9	5.5
VAT1_HUMAN	Synaptic vesicle membrane protein VAT-1 homolog	**2**	79.5 (M:79.5)	**3**	157.0 (M:157.0)	4	5.0
VNN1_HUMAN	Pantetheinase	**2**	135.7 (M:135.7)	**3**	183.4 (M:183.4)	4	5.0
ASSY_HUMAN	Argininosuccinate synthase	**2**	103.9 (M:103.9)	**3**	173.6 (M:173.6)	4	5.0
DCD_HUMAN	Dermcidin	**3**	179.4 (M:179.4)	**2**	99.6 (M:99.6)	4	5.0
DSG1_HUMAN	Desmoglein-1	**4**	189.2 (M:189.2)	**1**	31.6 (M:31.6)	4	5.0
SCRB2_HUMAN	Lysosome membrane protein 2	**3**	147.6 (M:147.6)	**2**	125.2 (M:125.2)	4	5.0
PCD12_HUMAN	Protocadherin-12	**2**	192.8 (M:192.8)	**3**	137.4 (M:137.4)	4	5.0
GLYC_HUMAN	Serine hydroxymethyltransferase, cytosolic	**3**	194.2 (M:194.2)	**2**	138.4 (M:138.4)	4	5.0
SAMP_HUMAN	Serum amyloid P-component	**3**	165.8 (M:165.8)	**2**	114.1 (M:114.1)	4	5.0
AMPE_HUMAN	Glutamyl aminopeptidase	**15**	809.3 (M:809.3)	**14**	694.8 (M:694.8)	23	4.8
PPAL_HUMAN	Lysosomal acid phosphatase	**7**	329.1 (M:329.1)	**7**	379.8 (M:379.8)	11	4.7
PROZ_HUMAN	Vitamin K-dependent protein Z	**9**	433.5 (M:433.5)	**7**	462.3 (M:462.3)	12	4.0
AL1A1_HUMAN	Retinal dehydrogenase 1	**3**	122.5 (M:122.5)	**5**	186.6 (M:186.6)	6	4.0
LTBP2_HUMAN	Latent-transforming growth factor beta-binding protein 2	**2**	72.3 (M:72.3)	**2**	121.0 (M:121.0)	3	4.0
PGM2_HUMAN	Phosphoglucomutase-2	**2**	73.7 (M:73.7)	**2**	97.2 (M:97.2)	3	4.0
S36A2_HUMAN	Proton-coupled amino acid transporter 2	**2**	73.3 (M:73.3)	**2**	98.7 (M:98.7)	3	4.0
6PGD_HUMAN	6-phosphogluconate dehydrogenase, decarboxylating	**1**	31.6 (M:31.6)	**3**	89.4 (M:89.4)	3	4.0
HYAL1_HUMAN	Hyaluronidase-1	**2**	81.0 (M:81.0)	**2**	50.4 (M:50.4)	3	4.0
SBP1_HUMAN	Selenium-binding protein 1	**3**	86.8 (M:86.8)	**1**	25.6 (M:25.6)	3	4.0

Table S4. *Cont.*

**Accession**	**Protein Name**	**AAL1. #Peptides**	**AAL1. Scores**	**AAL2. #Peptides**	**AAL2. Scores**	**AAL-FT**	**Fold Change**
MGA_HUMAN	Maltase-glucoamylase, intestinal	**23**	1332.1 (M:1332.1)	**24**	1399.4 (M:1399.4)	35	3.9
MMP9_HUMAN	Matrix metalloproteinase-9	**12**	599.3 (M:599.3)	**15**	711.4 (M:711.4)	20	3.9
CHL1_HUMAN	Neural cell adhesion molecule L1-like protein	**4**	154.0 (M:154.0)	**7**	336.5 (M:336.5)	8	3.7
DPP2_HUMAN	Dipeptidyl peptidase 2	**5**	316.4 (M:316.4)	**6**	327.0 (M:327.0)	8	3.7
ACE_HUMAN	Angiotensin-converting enzyme	**4**	261.4 (M:261.4)	**3**	191.8 (M:191.8)	5	3.5
GSTM4_HUMAN	Glutathione S-transferase Mu 4	**3**	187.4 (M:187.4)	**4**	251.8 (M:251.8)	5	3.5
CPN2_HUMAN	Carboxypeptidase N subunit 2	**4**	360.4 (M:360.4)	**6**	449.9 (M:449.9)	7	3.3
IDHC_HUMAN	Isocitrate dehydrogenase [NADP] cytoplasmic	**4**	143.0 (M:143.0)	**6**	314.8 (M:314.8)	7	3.3
SUSD2_HUMAN	Sushi domain-containing protein 2	**8**	810.3 (M:810.3)	**8**	574.0 (M:574.0)	11	3.2
BTD_HUMAN	Biotinidase	**8**	630.9 (M:630.9)	**10**	711.7 (M:711.7)	12	3.0
PRG2_HUMAN	Bone marrow proteoglycan	**3**	103.9 (M:103.9)	**3**	132.6 (M:132.6)	4	3.0
STOM_HUMAN	Erythrocyte band 7 integral membrane protein	**3**	168.3 (M:168.3)	**3**	200.8 (M:200.8)	4	3.0
ARP3_HUMAN	Actin-related protein 3	**2**	77.9 (M:77.9)	**1**	52.2 (M:52.2)	2	3.0
CD5L_HUMAN	CD5 antigen-like	**1**	74.6 (M:74.6)	**2**	137.0 (M:137.0)	2	3.0
CO6A3_HUMAN	Collagen alpha-3(VI) chain	**1**	69.1 (M:69.1)	**2**	128.1 (M:128.1)	2	3.0
TCO1_HUMAN	Transcobalamin-1	**1**	34.0 (M:34.0)	**2**	110.3 (M:110.3)	2	3.0
TERA_HUMAN	Transitional endoplasmic reticulum ATPase	**1**	43.0 (M:43.0)	**2**	76.4 (M:76.4)	2	3.0
XPP2_HUMAN	Xaa-Pro aminopeptidase 2	**1**	47.0 (M:47.0)	**2**	94.6 (M:94.6)	2	3.0
ACPH_HUMAN	Acylamino-acid-releasing enzyme	**2**	99.3 (M:99.3)	**1**	57.7 (M:57.7)	2	3.0
CO5_HUMAN	Complement C5	**11**	593.2 (M:593.2)	**12**	566.1 (M:566.1)	15	2.9
CO2_HUMAN	Complement C2	**5**	214.2 (M:214.2)	**6**	299.0 (M:299.0)	7	2.8

Table S4. *Cont.*

**Accession**	**Protein Name**	**AAL1. #Peptides**	**AAL1. Scores**	**AAL2. #Peptides**	**AAL2. Scores**	**AAL-FT**	**Fold Change**
PCD24_HUMAN	Protocadherin-24	**9**	510.3 (M:510.3)	**10**	588.6 (M:588.6)	12	2.7
CO9_HUMAN	Complement component C9	**9**	539.0 (M:539.0)	**14**	751.6 (M:751.6)	14	2.6
AT1A1_HUMAN	Sodium/potassium-transporting ATPase subunit alpha-1	**3**	169.2 (M:169.2)	**2**	98.2 (M:98.2)	3	2.5
L1CAM_HUMAN	Neural cell adhesion molecule L1	**3**	139.8 (M:139.8)	**2**	82.1 (M:82.1)	3	2.5
MA2B1_HUMAN	Lysosomal alpha-mannosidase	**2**	117.7 (M:117.7)	**3**	123.4 (M:123.4)	3	2.5
MANBA_HUMAN	Beta-mannosidase	**2**	87.8 (M:87.8)	**3**	154.6 (M:154.6)	3	2.5
MDHC_HUMAN	Malate dehydrogenase, cytoplasmic	**3**	139.3 (M:139.3)	**2**	96.1 (M:96.1)	3	2.5
RINI_HUMAN	Ribonuclease inhibitor	**2**	138.7 (M:138.7)	**3**	182.9 (M:182.9)	3	2.5
YS019_HUMAN	Transmembrane protein HSPC323	**3**	150.6 (M:150.6)	**2**	92.4 (M:92.4)	3	2.5
OLFM4_HUMAN	Olfactomedin-4	**9**	549.2 (M:549.2)	**10**	607.2 (M:607.2)	11	2.4
QSOX1_HUMAN	Sulfhydryl oxidase 1	**7**	266.5 (M:266.5)	**7**	356.9 (M:356.9)	8	2.3
PTPRG_HUMAN	Receptor-type tyrosine-protein phosphatase gamma	**3**	141.0 (M:141.0)	**4**	120.1 (M:120.1)	4	2.3
TGM4_HUMAN	Protein-glutamine gamma-glutamyltransferase 4	**4**	208.8 (M:208.8)	**3**	155.5 (M:155.5)	4	2.3
NEP_HUMAN	Neprilysin	**9**	616.2 (M:616.2)	**14**	803.8 (M:803.8)	13	2.3
CO8B_HUMAN	Complement component C8 beta chain	**6**	341.9 (M:341.9)	**5**	225.7 (M:225.7)	6	2.2
COFA1_HUMAN	Collagen alpha-1(XV) chain	**7**	283.6 (M:283.6)	**4**	221.8 (M:221.8)	6	2.2
ATRN_HUMAN	Attractin	**13**	1029.0 (M:1029.0)	**18**	1200.4 (M:1200.4)	16	2.1
IPSP_HUMAN	Plasma serine protease inhibitor	**15**	973.3 (M:973.3)	**18**	942.5 (M:942.5)	17	2.1
PGK1_HUMAN	Phosphoglycerate kinase 1	**7**	402.6 (M:402.6)	**5**	347.6 (M:347.6)	6	2.0
6PGL_HUMAN	6-phosphogluconolactonase	**4**	166.7 (M:166.7)	**4**	235.6 (M:235.6)	4	2.0
DPEP1_HUMAN	Dipeptidase 1	**4**	225.7 (M:225.7)	**4**	252.6 (M:252.6)	4	2.0

Table S4. *Cont.*

**Accession**	**Protein Name**	**AAL1. #Peptides**	**AAL1. Scores**	**AAL2. #Peptides**	**AAL2. Scores**	**AAL-FT**	**Fold Change**
ARP2_HUMAN	Actin-related protein 2	**2**	125.7 (M:125.7)	**2**	71.8 (M:71.8)	2	2.0
CAZA1_HUMAN	F-actin-capping protein subunit alpha-1	**2**	70.9 (M:70.9)	**2**	99.4 (M:99.4)	2	2.0
GDIB_HUMAN	Rab GDP dissociation inhibitor beta	**2**	85.0 (M:85.0)	**2**	124.4 (M:124.4)	2	2.0
KV305_HUMAN	Ig kappa chain V-III region WOL	**2**	210.0 (M:210.0)	**2**	248.1 (M:248.1)	2	2.0
LYAM1_HUMAN	L-selectin	**2**	107.5 (M:107.5)	**2**	125.0 (M:125.0)	2	2.0
SDCB1_HUMAN	Syntenin-1	**2**	65.7 (M:65.7)	**2**	40.9 (M:40.9)	2	2.0
TENX_HUMAN	Tenascin-X	**2**	92.7 (M:92.7)	**2**	84.8 (M:84.8)	2	2.0
TPP1_HUMAN	Tripeptidyl-peptidase 1	**2**	133.5 (M:133.5)	**2**	131.6 (M:131.6)	2	2.0
ZG16B_HUMAN	Zymogen granule protein 16 homolog B	**2**	188.6 (M:188.6)	**2**	172.6 (M:172.6)	2	2.0
BODG_HUMAN	Gamma-butyrobetaine dioxygenase	**1**	56.2 (M:56.2)	**1**	27.4 (M:27.4)	1	2.0
DSG2_HUMAN	Desmoglein-2	**1**	38.6 (M:38.6)	**1**	46.0 (M:46.0)	1	2.0
EPHAA_HUMAN	Ephrin type-A receptor 10	**1**	33.1 (M:33.1)	**1**	40.8 (M:40.8)	1	2.0
GALNS_HUMAN	N-acetylgalactosamine-6-sulfatase	**1**	25.9 (M:25.9)	**1**	34.1 (M:34.1)	1	2.0
GBG12_HUMAN	Guanine nucleotide-binding protein G(I)/G(S)/G(O) subunit gamma-12	**1**	56.0 (M:56.0)	**1**	33.8 (M:33.8)	1	2.0
HV207_HUMAN	Ig heavy chain V-II region NEWM	**1**	73.0 (M:73.0)	**1**	109.5 (M:109.5)	1	2.0
KV309_HUMAN	Ig kappa chain V-III region VG (Fragment)	**1**	44.8 (M:44.8)	**1**	45.5 (M:45.5)	1	2.0
PPT1_HUMAN	Palmitoyl-protein thioesterase 1	**1**	58.4 (M:58.4)	**1**	25.0 (M:25.0)	1	2.0
ARPC4_HUMAN	Actin-related protein 2/3 complex subunit 4	**1**	32.9 (M:32.9)	**1**	60.5 (M:60.5)	1	2.0
APOA_HUMAN	Apolipoprotein(a)	**1**	35.4 (M:35.4)	**1**	46.1 (M:46.1)	1	2.0
ARSF_HUMAN	Arylsulfatase F	**1**	68.1 (M:68.1)	**1**	74.4 (M:74.4)	1	2.0

Table S4. *Cont.*

**Accession**	**Protein Name**	**AAL1. #Peptides**	**AAL1. Scores**	**AAL2. #Peptides**	**AAL2. Scores**	**AAL-FT**	**Fold Change**
NAR3_HUMAN	Ecto-ADP-ribosyltransferase 3	**1**	48.5 (M:48.5)	**1**	66.9 (M:66.9)	1	2.0
SAP3_HUMAN	Ganglioside GM2 activator	**1**	28.0 (M:28.0)	**1**	27.6 (M:27.6)	1	2.0
GPX3_HUMAN	Glutathione peroxidase 3	**1**	25.8 (M:25.8)	**1**	36.0 (M:36.0)	1	2.0
HABP2_HUMAN	Hyaluronan-binding protein 2	**1**	44.5 (M:44.5)	**1**	60.3 (M:60.3)	1	2.0

Table S5. Proteins in both whole urine duplicates for the pTa v control urine experiment. Proteins are sorted according to average H/L ratio (pTa/control).

ACCESSION	Protein Name	WHOLE 1 #Peptides	WHOLE 1 Score	WHOLE 1 H/L	WHOLE 2 #Peptides	WHOLE 2 Score	WHOLE 2 H/L	*p* Value
MUC5B_HUMAN	Mucin-5B	10	645.6	13.24	7	292.4	7.96	0.0009
S12A3_HUMAN	Solute carrier family 12 member 3	1	44.7	9.7	1	32.3	3.03	0.0178
TGM4_HUMAN	Protein-glutamine gamma-glutamyltransferase 4	5	216.3	8.25	4	187.2	3.42	0.0091
T106B_HUMAN	Transmembrane protein 106B	2	63.1	5.61	1	40.1	0.87	0.2486
UROM_HUMAN	Uromodulin	35	2127.9	2.54	38	2137.7	2.73	0.0009
MASP2_HUMAN	Mannan-binding lectin serine protease 2	8	538	2.16	7	465	2.63	0.0025
TSP1_HUMAN	Thrombospondin-1	1	52.1	2.81	2	78.3	1.27	0.0814
A1AG2_HUMAN	Alpha-1-acid glycoprotein 2	17	1056.8	2	19	1115.9	2.05	0.0021
SPHM_HUMAN	N-sulphoglucosamine sulphohydrolase	3	200.4	1.56	4	127.7	2.19	0.0154
K1C10_HUMAN	Keratin, type I cytoskeletal 10	25	1575.6	0.93	25	1629.9	2.75	0.2434
SAP_HUMAN	Proactivator polypeptide	5	255.4	2.58	6	263.2	1.1	0.1398
SAMP_HUMAN	Serum amyloid P-component	3	205.5	2.19	3	186	1.38	0.0361
UROK_HUMAN	Urokinase-type plasminogen activator	3	164.7	1.82	3	120.2	1.72	0.004
TRFM_HUMAN	Melanotransferrin	1	35.7	0.94	3	78.6	2.52	0.2414
CATZ_HUMAN	Cathepsin Z	3	109.2	1.82	3	145.9	1.6	0.0067
CAD11_HUMAN	Cadherin-11	4	152.1	0.99	7	295	2.37	0.2079
DPP4_HUMAN	Dipeptidyl peptidase 4	8	393.7	1.73	3	152.6	1.63	0.0054
ZA2G_HUMAN	Zinc-alpha-2-glycoprotein	40	2175.3	1.84	40	2103.7	1.49	0.0123
PRDX6_HUMAN	Peroxiredoxin-6	4	177.2	1.88	4	186.6	1.42	0.0198
SAP3_HUMAN	Ganglioside GM2 activator	10	443.4	1.76	6	289.2	1.53	0.0086

Table S5. *Cont.*

ACCESSION	Protein Name	WHOLE 1 #Peptides	WHOLE 1 Score	WHOLE 1 H/L	WHOLE 2 #Peptides	WHOLE 2 Score	WHOLE 2 H/L	*p* Value
DPP2_HUMAN	Dipeptidyl peptidase 2	3	173.8	1.69	2	120.4	1.55	0.0071
IPSP_HUMAN	Plasma serine protease inhibitor	21	1200.5	1.6	19	1060.1	1.62	0.0062
FUCO_HUMAN	Tissue alpha-L-fucosidase	4	205.5	1.83	1	34.6	1.36	0.0266
CALL3_HUMAN	Calmodulin-like protein 3	2	70.8	1.43	3	114.1	1.75	0.0154
GNS_HUMAN	N-acetylglucosamine-6-sulfatase	13	660.8	1.68	10	445.2	1.5	0.0092
FBN1_HUMAN	Fibrillin-1	4	177.9	1.54	4	166.1	1.63	0.0074
IBP7_HUMAN	Insulin-like growth factor-binding protein 7	15	912.8	1.53	14	653.5	1.61	0.0077
K2C1_HUMAN	Keratin, type II cytoskeletal 1	36	2541.4	0.83	38	2445.5	2.3	0.3687
CADH2_HUMAN	Cadherin-2	7	305.5	1.52	4	205.7	1.61	0.0079
NADC_HUMAN	Nicotinate-nucleotide pyrophosphorylase [carboxylating]	1	72.8	1.82	2	83.2	1.26	0.0484
DIAC_HUMAN	Di-N-acetylchitobiase	7	305.9	1.38	3	98.7	1.68	0.0184
FGL2_HUMAN	Fibroleukin	1	47.3	1.67	1	31.4	1.38	0.0189
FBLN4_HUMAN	EGF-containing fibulin-like extracellular matrix protein 2	1	78.4	1.3	2	98.3	1.75	0.0351
CPVL_HUMAN	Probable serine carboxypeptidase CPVL	4	155.8	1.51	4	150.7	1.52	0.0089
S12A1_HUMAN	Solute carrier family 12 member 1	7	334	1.5	2	78.6	1.52	0.0095
EPHB6_HUMAN	Ephrin type-B receptor 6	2	69.3	1.69	3	168.9	1.32	0.0292
S10AE_HUMAN	Protein S100-A14	1	65.8	1.08	1	66.2	1.88	0.1368
CD14_HUMAN	Monocyte differentiation antigen CD14	14	785	1.53	13	678.4	1.42	0.0129
A1AG1_HUMAN	Alpha-1-acid glycoprotein 1	17	1135.9	1.44	18	1221	1.51	0.0116
CATB_HUMAN	Cathepsin B	1	44.4	1.39	4	173.3	1.54	0.0145

Table S5. *Cont.*

ACCESSION	Protein Name	WHOLE 1 #Peptides	WHOLE 1 Score	WHOLE 1 H/L	WHOLE 2 #Peptides	WHOLE 2 Score	WHOLE 2 H/L	*p* Value
NEP_HUMAN	Neprilysin	15	649.3	1.5	11	595.7	1.42	0.0129
ASAH1_HUMAN	Acid ceramidase	7	319.8	1.44	6	250.6	1.47	0.0122
PROZ_HUMAN	Vitamin K-dependent protein Z	6	272.4	1.64	6	265.4	1.26	0.0396
MUCDL_HUMAN	Mucin and cadherin-like protein	1	67.3	1.85	2	74.6	1.05	0.1599
YS019_HUMAN	Transmembrane protein HSPC323	2	93.3	1.54	2	77.5	1.36	0.0176
LYVE1_HUMAN	Lymphatic vessel endothelial hyaluronic acid receptor 1	4	204.4	1.54	3	160.7	1.36	0.0176
PGCP_HUMAN	Plasma glutamate carboxypeptidase	8	393.9	1.45	9	450	1.45	0.0124
QPCT_HUMAN	Glutaminyl-peptide cyclotransferase	8	500	1.3	6	325.7	1.59	0.0285
TPP1_HUMAN	Tripeptidyl-peptidase 1	4	202.4	1.4	3	129.7	1.48	0.0139
PODXL_HUMAN	Podocalyxin-like protein 1	8	447.3	1.26	6	290.9	1.6	0.0375
NAPSA_HUMAN	Napsin-A	7	534.1	1.74	7	397.5	1.12	0.1063
ARSF_HUMAN	Arylsulfatase F	2	119.4	1.48	2	119.8	1.38	0.0151
HS90B_HUMAN	Heat shock protein HSP 90-beta	8	504.5	1.22	6	333.4	1.62	0.0501
CETP_HUMAN	Cholesteryl ester transfer protein	1	31.5	1.39	2	64.7	1.45	0.0148
ARSA_HUMAN	Arylsulfatase A	10	611.5	1.41	7	386.2	1.43	0.0143
GSTM3_HUMAN	Glutathione S-transferase Mu 3	1	35.1	1.25	1	48.1	1.58	0.0392
TWSG1_HUMAN	Twisted gastrulation protein homolog 1	5	218.5	1.68	5	227	1.15	0.0852
CILP2_HUMAN	Cartilage intermediate layer protein 2	3	124.8	1.02	2	59.8	1.81	0.1926
GLYC_HUMAN	Serine hydroxymethyltransferase, cytosolic	1	31.6	1.41	2	90	1.41	0.0152
MUC1_HUMAN	Mucin-1	6	299.2	1.53	5	203.7	1.28	0.0293

Table S5. *Cont.*

ACCESSION	Protein Name	WHOLE 1 #Peptides	WHOLE 1 Score	WHOLE 1 H/L	WHOLE 2 #Peptides	WHOLE 2 Score	WHOLE 2 H/L	*p* Value
SPIT1_HUMAN	Kunitz-type protease inhibitor 1	2	84.5	0.88	1	33.5	1.93	0.3543
ANXA5_HUMAN	Annexin A5	4	222.1	1.34	3	140.5	1.47	0.0198
NEUR1_HUMAN	Sialidase-1	4	187.4	1.55	3	109.6	1.25	0.0375
KV402_HUMAN	Ig kappa chain V-IV region Len	4	441.6	1.57	4	401.3	1.23	0.0451
VATH_HUMAN	V-type proton ATPase subunit H	2	62.1	1.26	1	30	1.53	0.0346
CALX_HUMAN	Calnexin	1	61.9	1.53	1	28.1	1.25	0.0367
SDC1_HUMAN	Syndecan-1	2	147.2	1.37	2	132.1	1.41	0.0172
MGA_HUMAN	Maltase-glucoamylase, intestinal	20	970	1.37	13	655.8	1.39	0.0178
BODG_HUMAN	Gamma-butyrobetaine dioxygenase	2	106.7	1.53	1	35.1	1.22	0.046
PLD3_HUMAN	Phospholipase D3	5	259.8	1.48	5	189.5	1.28	0.0293
AMPE_HUMAN	Glutamyl aminopeptidase	9	393.5	1.25	6	308.5	1.49	0.0355
HEXA_HUMAN	Beta-hexosaminidase subunit alpha	4	135	1.21	4	145	1.53	0.0525
LMAN2_HUMAN	Vesicular integral-membrane protein VIP36	18	870.3	1.4	14	661.8	1.33	0.021
GGH_HUMAN	Gamma-glutamyl hydrolase	8	418.7	1.43	9	397.5	1.3	0.0246
UBIQ_HUMAN	Ubiquitin	1	51.5	1.14	2	117.7	1.58	0.0887
CADH1_HUMAN	Cadherin-1	13	896.8	1.05	11	761.6	1.68	0.1668
PLBL2_HUMAN	Putative phospholipase B-like 2	2	100.6	1.38	2	81.4	1.34	0.0208
LEG9_HUMAN	Galectin-9	2	154.8	1.41	2	105	1.3	0.0247
ANXA4_HUMAN	Annexin A4	6	258.2	1.52	5	317.5	1.18	0.065
AQP1_HUMAN	Aquaporin-1	2	152.9	1.36	2	182.6	1.33	0.023
CUBN_HUMAN	Cubilin	46	2573.4	1.31	39	2180.7	1.38	0.0243
THBG_HUMAN	Thyroxine-binding globulin	9	532.2	0.87	10	567.4	1.81	0.3908
LRP2_HUMAN	Low-density lipoprotein receptor-related protein 2	36	2082.2	1.41	32	1755.9	1.26	0.0319

Table S5. *Cont.*

ACCESSION	Protein Name	WHOLE 1 #Peptides	WHOLE 1 Score	WHOLE 1 H/L	WHOLE 2 #Peptides	WHOLE 2 Score	WHOLE 2 H/L	*p* Value
MMRN2_HUMAN	Multimerin-2	2	90	1.32	2	86.6	1.35	0.0247
CMBL_HUMAN	Carboxymethylenebutenolidase homolog	1	33.2	1.44	1	47.5	1.22	0.0426
CD9_HUMAN	CD9 antigen	3	136.4	1.3	3	97.6	1.36	0.0262
LPHN1_HUMAN	Latrophilin-1	3	100.5	1.28	2	88.7	1.38	0.0285
INAR2_HUMAN	Interferon alpha/beta receptor 2	3	108	1.32	1	55.8	1.33	0.0259
AT1A1_HUMAN	Sodium/potassium-transporting ATPase subunit alpha-1	2	162.7	1.43	2	80.2	1.22	0.0453
LDHB_HUMAN	L-lactate dehydrogenase B chain	10	489.4	1.36	7	301.4	1.28	0.0286
ANGL2_HUMAN	Angiopoietin-related protein 2	5	191	1.39	4	198.4	1.25	0.0357
VMO1_HUMAN	Vitelline membrane outer layer protein 1 homolog	7	459.4	1.43	7	496.4	1.21	0.0481
CAD16_HUMAN	Cadherin-16	2	72.7	1.33	1	57.3	1.31	0.0272
K2C5_HUMAN	Keratin, type II cytoskeletal 5	18	953.2	0.53	16	954.6	2.1	0.9001
ASSY_HUMAN	Argininosuccinate synthase	4	181.6	1.36	2	91.3	1.28	0.0298
GSLG1_HUMAN	Golgi apparatus protein 1	5	206.8	1.39	5	282.4	1.24	0.0375
CADM4_HUMAN	Cell adhesion molecule 4	8	602.7	1.54	9	558	1.08	0.1372
BHMT1_HUMAN	Betaine--homocysteine S-methyltransferase 1	6	436.4	1.29	7	348.1	1.32	0.0298
STC1_HUMAN	Stanniocalcin-1	1	76.9	1.44	1	74.7	1.17	0.0654
SLAF5_HUMAN	SLAM family member 5	3	215.6	1.36	4	245.2	1.24	0.0378
ANAG_HUMAN	Alpha-N-acetylglucosaminidase	23	1231.6	1.32	16	867.8	1.28	0.031
PSCA_HUMAN	Prostate stem cell antigen	3	236.1	1.18	2	105.2	1.42	0.0608
DPEP1_HUMAN	Dipeptidase 1	4	156.2	1.33	6	223.1	1.27	0.0327
NAGAB_HUMAN	Alpha-N-acetylgalactosaminidase	4	252.3	1.17	2	73.8	1.42	0.0659

Table S5. *Cont.*

ACCESSION	Protein Name	WHOLE 1 #Peptides	WHOLE 1 Score	WHOLE 1 H/L	WHOLE 2 #Peptides	WHOLE 2 Score	WHOLE 2 H/L	*p* Value
VCAM1_HUMAN	Vascular cell adhesion protein 1	7	275.7	1.3	5	252.9	1.28	0.0339
PGCA_HUMAN	Aggrecan core protein	1	53.5	1.13	3	81.8	1.44	0.0899
TRBM_HUMAN	Thrombomodulin	3	163.9	1.3	3	122.4	1.27	0.0351
SIRB1_HUMAN	Signal-regulatory protein beta-1	4	186.5	1.22	3	133.6	1.34	0.0427
GSTA1_HUMAN	Glutathione S-transferase A1	2	75.6	1	4	143.3	1.56	0.2286
PPGB_HUMAN	Lysosomal protective protein	7	438.2	1.33	6	320.8	1.22	0.0462
PGS1_HUMAN	Biglycan	3	158.3	1.79	3	104.5	0.75	0.6113
MA1A1_HUMAN	Mannosyl-oligosaccharide 1,2-alpha-mannosidase IA	9	429	1.32	3	114.1	1.22	0.0467
CO6A1_HUMAN	Collagen alpha-1(VI) chain	20	1155.4	1.35	19	1167.9	1.18	0.0575
TM7S3_HUMAN	Transmembrane 7 superfamily member 3	2	129.1	1.32	1	44.9	1.21	0.0493
IGJ_HUMAN	Immunoglobulin J chain	4	159.2	1.92	2	117.2	0.61	0.8396
MXRA8_HUMAN	Matrix-remodeling-associated protein 8	5	260.9	1.34	6	257.3	1.18	0.0578
EGF_HUMAN	Pro-epidermal growth factor	41	2223	1.24	37	1809.6	1.28	0.0428
PRDX1_HUMAN	Peroxiredoxin-1	2	94.3	1.06	1	58.9	1.46	0.1612
CAD15_HUMAN	Cadherin-15	1	75.7	1.34	3	121.2	1.18	0.0613
A4_HUMAN	Amyloid beta A4 protein	3	116.5	1.39	1	84.2	1.12	0.1012
K22E_HUMAN	Keratin, type II cytoskeletal 2 epidermal	28	1961.8	1.07	29	1869.6	1.43	0.152
KLK1_HUMAN	Kallikrein-1	6	279.5	0.93	6	345.1	1.55	0.3388
GNAS2_HUMAN	Guanine nucleotide-binding protein G(s) subunit alpha isoforms short	1	36.9	1.31	3	121.6	1.18	0.0628
NEGR1_HUMAN	Neuronal growth regulator 1	1	101.8	1.26	2	126.1	1.22	0.0502

Table S5. *Cont.*

ACCESSION	Protein Name	WHOLE 1 #Peptides	WHOLE 1 Score	WHOLE 1 H/L	WHOLE 2 #Peptides	WHOLE 2 Score	WHOLE 2 H/L	*p* Value
HEXB_HUMAN	Beta-hexosaminidase subunit beta	5	236.9	1.25	3	130.5	1.23	0.0496
ICOSL_HUMAN	IC	6	383.7	1.15	5	363.4	1.33	0.0772
VDAC1_HUMAN	Voltage-dependent anion-selective channel protein 1	4	239.5	1.49	2	68	0.98	0.2635
BCAM_HUMAN	Basal cell adhesion molecule	7	315.9	1.16	5	215	1.31	0.0725
SODE_HUMAN	Extracellular superoxide dismutase [Cu-Zn]	9	647.5	1.22	7	355.1	1.23	0.0561
DAF_HUMAN	Complement decay-accelerating factor	9	505.6	1.28	9	393	1.17	0.0691
EPHA4_HUMAN	Ephrin type-A receptor 4	1	92.7	1.32	4	278.2	1.13	0.0916
BGAL_HUMAN	Beta-galactosidase	11	688.2	1.27	11	651.1	1.18	0.0665
AMY1_HUMAN	Alpha-amylase 1	25	1629	1.23	21	1418.8	1.21	0.0607
HEG1_HUMAN	Protein HEG homolog 1	4	267.3	1.08	4	199	1.35	0.1338
AGRIN_HUMAN	Agrin	6	368.3	1.45	2	99	0.98	0.2713
HMCN1_HUMAN	Hemicentin-1	9	623.1	1.16	8	500.3	1.27	0.0766
FETUA_HUMAN	Alpha-2-HS-glycoprotein	8	485.4	1.64	7	396.5	0.79	0.6027
LG3BP_HUMAN	Galectin-3-binding protein	20	1261.2	1.33	11	686	1.1	0.1185
ENDD1_HUMAN	Endonuclease domain-containing 1 protein	15	1041.1	1.4	17	979.7	1.02	0.214
GPC5C_HUMAN	G-protein coupled receptor family C group 5 member C	7	459	1.17	6	279.5	1.25	0.074
PDC6I_HUMAN	Programmed cell death 6-interacting protein	7	406.7	1.32	3	151.8	1.1	0.1193
EPHB4_HUMAN	Ephrin type-B receptor 4	4	166.9	1.38	8	277.3	1.03	0.2043
QSOX1_HUMAN	Sulfhydryl oxidase 1	6	245	1.41	6	231.7	1	0.2522
CATC_HUMAN	Dipeptidyl peptidase 1	3	192.4	1.22	2	148	1.18	0.0723

Table S5. *Cont.*

ACCESSION	Protein Name	WHOLE 1 #Peptides	WHOLE 1 Score	WHOLE 1 H/L	WHOLE 2 #Peptides	WHOLE 2 Score	WHOLE 2 H/L	*p* Value
B3GN1_HUMAN	N-acetyllactosaminide beta-1,3-N-acetylglucosaminyltransferase	4	381.4	1.18	2	234.8	1.23	0.0737
JAM3_HUMAN	Junctional adhesion molecule C	3	174.4	1.3	2	98	1.11	0.1135
OLR1_HUMAN	Oxidized low-density lipoprotein receptor 1	2	147.7	1.26	3	125.1	1.13	0.0983
RNT2_HUMAN	Ribonuclease T2	1	48.4	1.21	1	30.1	1.18	0.0786
PCP_HUMAN	Lysosomal Pro-X carboxypeptidase	1	60.2	1.05	1	81.3	1.34	0.1867
ENPL_HUMAN	Endoplasmin	4	150.9	1.14	3	136.2	1.24	0.0954
K1C9_HUMAN	Keratin, type I cytoskeletal 9	36	2100.5	0.76	22	1219.6	1.61	0.7002
PAPP2_HUMAN	Pappalysin-2	6	269.6	1.19	5	205.6	1.18	0.0895
PCDGK_HUMAN	Protocadherin gamma-C3	2	100.2	1.21	2	131.8	1.15	0.0974
OSCAR_HUMAN	Osteoclast-associated immunoglobulin-like receptor	2	87.6	1.21	3	85.4	1.15	0.0974
GNAI3_HUMAN	Guanine nucleotide-binding protein G(k) subunit alpha	1	54.2	1.17	2	84.9	1.19	0.0918
CD81_HUMAN	CD81 antigen	2	94.6	1.15	1	70	1.21	0.0977
FCG3A_HUMAN	Low affinity immunoglobulin gamma Fc region receptor III-A	3	136.6	1.15	3	147.8	1.21	0.0977
ANXA6_HUMAN	Annexin A6	2	72.4	1.26	2	68.4	1.09	0.1379
IL1AP_HUMAN	Interleukin-1 receptor accessory protein	2	119.6	1.07	3	123.2	1.28	0.1696
PGFRB_HUMAN	Beta-type platelet-derived growth factor receptor	1	43.9	1.07	3	94.7	1.28	0.1696
ANPRC_HUMAN	Atrial natriuretic peptide receptor 3	2	112.2	1.15	1	33.7	1.19	0.1038
ROBO4_HUMAN	Roundabout homolog 4	11	639.2	1.16	11	643.9	1.18	0.1048

Table S5. *Cont.*

ACCESSION	Protein Name	WHOLE 1 #Peptides	WHOLE 1 Score	WHOLE 1 H/L	WHOLE 2 #Peptides	WHOLE 2 Score	WHOLE 2 H/L	*p* Value
GGT1_HUMAN	Gamma-glutamyltranspeptidase 1	11	634.3	1.33	7	399.3	1.01	0.2616
AMYP_HUMAN	Pancreatic alpha-amylase	26	1676.8	1.14	21	1397.4	1.18	0.1151
CNTN1_HUMAN	Contactin-1	4	187.9	1.21	5	176.8	1.12	0.128
CD44_HUMAN	CD44 antigen	7	395.1	1.33	5	290.9	0.99	0.295
HYAL1_HUMAN	Hyaluronidase-1	7	284.4	1.2	3	148	1.12	0.1249
PGRP1_HUMAN	Peptidoglycan recognition protein 1	5	336	1.27	4	230.4	1.05	0.2054
KPYM_HUMAN	Pyruvate kinase isozymes M1/M2	8	363.5	0.87	11	481.1	1.45	0.5356
LIRA5_HUMAN	Leukocyte immunoglobulin-like receptor subfamily A member 5	2	106.3	1.21	2	70.8	1.11	0.1366
EZRI_HUMAN	Ezrin	6	211.1	1.04	6	240.8	1.27	0.2225
AGAL_HUMAN	Alpha-galactosidase A	7	368.6	1.4	6	285.5	0.9	0.486
MA2B2_HUMAN	Epididymis-specific alpha-mannosidase	8	441.7	1.14	4	206.4	1.16	0.1288
DDR1_HUMAN	Epithelial discoidin domain-containing receptor 1	2	153.4	1.35	4	172.9	0.95	0.3796
LAMP1_HUMAN	Lysosome-associated membrane glycoprotein 1	3	209.6	1.01	1	30.7	1.28	0.2766
GALM_HUMAN	Aldose 1-epimerase	2	86.2	1.22	1	41.9	1.07	0.1827
L1CAM_HUMAN	Neural cell adhesion molecule L1	3	106.3	1.16	2	89.8	1.13	0.1393
CLM1_HUMAN	CMRF35-like molecule 1	2	81.3	1.14	1	61.9	1.15	0.1406
PCD24_HUMAN	Protocadherin-24	11	611.4	1.18	9	433.4	1.11	0.1515
VATA_HUMAN	V-type proton ATPase catalytic subunit A	2	102	1.07	3	178	1.22	0.1949
TIMD3_HUMAN	Hepatitis A virus cellular receptor 2	2	123.5	1.21	1	71.2	1.07	0.1922
OPCM_HUMAN	Opioid-binding protein/cell adhesion molecule	4	251.1	1.21	3	223	1.07	0.1922

Table S5. *Cont.*

ACCESSION	Protein Name	WHOLE 1 #Peptides	WHOLE 1 Score	WHOLE 1 H/L	WHOLE 2 #Peptides	WHOLE 2 Score	WHOLE 2 H/L	*p* Value
RNAS1_HUMAN	Ribonuclease pancreatic	5	325.6	1.2	3	195.6	1.08	0.1848
PRG2_HUMAN	Bone marrow proteoglycan	4	173	1.1	2	60.3	1.17	0.164
SDC4_HUMAN	Syndecan-4	3	187.6	1.13	2	133.5	1.14	0.1569
PTGDS_HUMAN	Prostaglandin-H2 D-isomerase	9	697.9	1.39	8	585.7	0.87	0.5783
PDZ1P_HUMAN	Putative PDZ domain-containing protein 1P	1	97	1.13	2	108.1	1.13	0.165
PTPRG_HUMAN	Receptor-type tyrosine-protein phosphatase gamma	4	216.2	1.16	5	290.5	1.1	0.1753
IF6_HUMAN	Eukaryotic translation initiation factor 6	3	91.3	1.17	3	115	1.08	0.1925
CNDP2_HUMAN	Cytosolic non-specific dipeptidase	5	207.6	1.21	2	66.6	1.05	0.2382
LYAG_HUMAN	Lysosomal alpha-glucosidase	28	1654.2	1.15	19	1027.1	1.1	0.1839
ANTR1_HUMAN	Anthrax toxin receptor 1	1	69	1.17	2	134.5	1.08	0.2054
IGHA1_HUMAN	Ig alpha-1 chain C region	22	1430.9	1.15	21	1385.5	1.09	0.1967
PI16_HUMAN	Peptidase inhibitor 16	11	652.6	1.14	5	290.5	1.1	0.1935
MYH9_HUMAN	Myosin-9	9	491.1	1	10	532.6	1.24	0.3285
GILT_HUMAN	Gamma-interferon-inducible lysosomal thiol reductase	2	107.7	1.09	1	58.1	1.14	0.206
EPHB2_HUMAN	Ephrin type-B receptor 2	2	123	1.26	4	253.2	0.97	0.3987
GRHPR_HUMAN	Glyoxylate reductase/hydroxypyruvate reductase	1	45.6	1.18	1	54.2	1.05	0.2611
ZG16B_HUMAN	Zymogen granule protein 16 homolog B	5	339.8	1.13	4	290	1.08	0.2314
LV302_HUMAN	Ig lambda chain V-III region LOI	1	51.7	1.2	1	35	1.02	0.3232
SORL_HUMAN	Sortilin-related receptor	2	65.4	1.15	2	63.3	1.06	0.2555

Table S5. *Cont.*

ACCESSION	Protein Name	WHOLE 1 #Peptides	WHOLE 1 Score	WHOLE 1 H/L	WHOLE 2 #Peptides	WHOLE 2 Score	WHOLE 2 H/L	*p* Value
MXRA5_HUMAN	Matrix-remodeling-associated protein 5	1	53.5	1.21	1	38	0.99	0.3729
CBPM_HUMAN	Carboxypeptidase M	3	235.4	1.25	5	239.2	0.95	0.4562
EPHB3_HUMAN	Ephrin type-B receptor 3	3	147.1	1.16	3	136	1.05	0.2809
CAB45_HUMAN	45 kDa calcium-binding protein	3	185.4	1.06	2	152.5	1.15	0.2714
ATRN_HUMAN	Attractin	13	815.4	1.18	10	720.7	1.02	0.3215
NEO1_HUMAN	Neogenin	1	72.8	1.21	2	113.5	0.99	0.3832
COFA1_HUMAN	Collagen alpha-1(XV) chain	2	69	1.06	1	36.1	1.14	0.2819
SUSD2_HUMAN	Sushi domain-containing protein 2	2	115.1	1	1	113.4	1.19	0.3746
8ODP_HUMAN	7,8-dihydro-8-oxoguanine triphosphatase	2	87.4	1.08	1	36.9	1.11	0.2653
MADCA_HUMAN	Mucosal addressin cell adhesion molecule 1	2	176.5	1.11	2	171.2	1.08	0.2746
SIRBL_HUMAN	Signal-regulatory protein beta-1 isoform 3	6	353.1	0.97	4	164.1	1.22	0.4401
GPC1_HUMAN	Glypican-1	1	77.4	1.22	1	31.2	0.96	0.4654
RAB10_HUMAN	Ras-related protein Rab-10	4	161.2	1.06	4	147.6	1.12	0.306
GDIB_HUMAN	Rab GDP dissociation inhibitor beta	2	75.6	1.04	1	45.9	1.14	0.333
P3IP1_HUMAN	Phosphoinositide-3-kinase-interacting protein 1	5	287	1.25	4	292.5	0.92	0.5913
BTD_HUMAN	Biotinidase	8	473	1.07	9	382.7	1.08	0.3353
CATH_HUMAN	Cathepsin H	4	160.8	1.14	2	79.1	1.02	0.4014
FA11_HUMAN	Coagulation factor XI	2	67.2	0.88	3	91.6	1.28	0.6852
C1RL_HUMAN	Complement C1r subcomponent-like protein	7	316.3	1.12	4	203.7	1.03	0.3841

Table S5. *Cont.*

ACCESSION	Protein Name	WHOLE 1 #Peptides	WHOLE 1 Score	WHOLE 1 H/L	WHOLE 2 #Peptides	WHOLE 2 Score	WHOLE 2 H/L	*p* Value
GLU2B_HUMAN	Glucosidase 2 subunit beta	1	32.6	0.99	1	41.3	1.16	0.4541
G3P_HUMAN	Glyceraldehyde-3-phosphate dehydrogenase	7	394.8	0.98	3	93.8	1.17	0.4737
AMPN_HUMAN	Aminopeptidase N	35	1805.7	1.07	30	1614.4	1.08	0.361
ACE_HUMAN	Angiotensin-converting enzyme	1	47.7	1.17	2	164.2	0.98	0.495
CHM2A_HUMAN	Charged multivesicular body protein 2a	1	48.6	1.07	2	129.2	1.07	0.3738
CSPG5_HUMAN	Chondroitin sulfate proteoglycan 5	2	104.9	1.03	1	30.4	1.12	0.4051
NTRI_HUMAN	Neurotrimin	2	149.1	1.11	1	83.7	1.03	0.4037
PGM1_HUMAN	Phosphoglucomutase-1	1	31	0.98	3	96.5	1.16	0.4883
ICAM2_HUMAN	Intercellular adhesion molecule 2	2	102.4	1.07	2	109.1	1.06	0.3975
MUC18_HUMAN	Cell surface glycoprotein MUC18	6	274.6	1.03	4	139.2	1.11	0.4225
PPAP_HUMAN	Prostatic acid phosphatase	18	894.7	1.06	20	955.3	1.07	0.4289
ALDOB_HUMAN	Fructose-bisphosphate aldolase B	7	404.4	1.25	6	288.3	0.87	0.7582
RAP1B_HUMAN	Ras-related protein Rap-1b	3	158	0.92	1	58.6	1.2	0.6805
SCTM1_HUMAN	Secreted and transmembrane protein 1	1	82.3	1.12	3	120.6	0.98	0.5649
FOLR1_HUMAN	Folate receptor alpha	6	339.4	1.07	5	297.1	1.03	0.5046
IQGA1_HUMAN	Ras GTPase-activating-like protein IQGAP1	1	37.4	0.79	1	56.9	1.31	0.9132
KNG1_HUMAN	Kininogen-1	31	2320.4	1.06	30	2143.6	1.04	0.5404
CAD13_HUMAN	Cadherin-13	5	281.5	1.09	5	337.6	1	0.5771
PEPA_HUMAN	Pepsin A	4	248.6	0.72	5	184.4	1.37	0.9721
AQP2_HUMAN	Aquaporin-2	3	158.5	1.12	2	101.9	0.96	0.6739
FSTL1_HUMAN	Follistatin-related protein 1	1	85.9	1.08	2	95.4	0.98	0.6803

Table S5. *Cont.*

ACCESSION	Protein Name	WHOLE 1 #Peptides	WHOLE 1 Score	WHOLE 1 H/L	WHOLE 2 #Peptides	WHOLE 2 Score	WHOLE 2 H/L	*p* Value
ANX11_HUMAN	Annexin A11	6	262.9	1.07	3	151.5	0.99	0.6793
PGBM_HUMAN	Basement membrane-specific heparan sulfate proteoglycan core protein	19	1166.7	0.89	22	1279.3	1.18	0.8505
CHL1_HUMAN	Neural cell adhesion molecule L1-like protein	3	134.5	1.1	3	125.2	0.96	0.7335
TSN1_HUMAN	Tetraspanin-1	2	148.1	1	2	125.2	1.06	0.6946
IDHC_HUMAN	Isocitrate dehydrogenase [NADP] cytoplasmic	5	255.6	1.21	3	148.6	0.85	0.9121
CRIS3_HUMAN	Cysteine-rich secretory protein 3	3	178.4	0.82	4	195.3	1.23	0.9663
APOD_HUMAN	Apolipoprotein D	16	1243.1	1.2	18	1240.1	0.85	0.934
GNAI2_HUMAN	Guanine nucleotide-binding protein G(i) subunit alpha-2	1	70	1.07	2	94.2	0.97	0.7982
TNR16_HUMAN	Tumor necrosis factor receptor superfamily member 16	5	273.9	1.11	4	241.6	0.92	0.8882
PRSS8_HUMAN	Prostasin	2	86.8	1.07	2	128.6	0.97	0.8393
CF072_HUMAN	Uncharacterized protein C6orf72	3	156.3	1.05	3	136	0.98	0.8393
CEL_HUMAN	Bile salt-activated lipase	8	359.3	0.98	7	319.5	1.05	0.8612
S100P_HUMAN	Protein S100-P	2	144.1	0.98	2	117.5	1.05	0.8612
PIGR_HUMAN	Polymeric immunoglobulin receptor	28	1731.1	0.99	32	1923.8	1.02	0.9282
CLIC1_HUMAN	Chloride intracellular channel protein 1	3	154.1	1.01	2	131.2	1	0.9504
OTUB1_HUMAN	Ubiquitin thioesterase OTUB1	1	41.9	0.94	1	38.8	1.06	0.9898
ANXA2_HUMAN	Annexin A2	14	1074.5	0.93	14	749.5	1.07	0.9967
CSPG4_HUMAN	Chondroitin sulfate proteoglycan 4	6	274.5	1	4	197.4	1	1

Table S5. *Cont.*

ACCESSION	Protein Name	WHOLE 1 #Peptides	WHOLE 1 Score	WHOLE 1 H/L	WHOLE 2 #Peptides	WHOLE 2 Score	WHOLE 2 H/L	*p* Value
KV405_HUMAN	Ig kappa chain V-IV region STH (Fragment)	2	141.2	1.21	2	153.6	0.79	0.8809
CD248_HUMAN	Endosialin	8	312.9	1.07	6	279	0.92	0.9651
LAMA4_HUMAN	Laminin subunit alpha-4	1	69.6	1.11	1	75.7	0.88	0.9355
PVRL4_HUMAN	Poliovirus receptor-related protein 4	5	197.3	1.11	3	125.6	0.88	0.9355
DSC2_HUMAN	Desmocollin-2	5	226.7	0.76	5	202.4	1.24	0.8528
AMBP_HUMAN	Protein AMBP	46	3379.4	1.09	47	3301.9	0.9	0.9334
CLM9_HUMAN	CMRF35-like molecule 9	5	343.2	1.02	6	465.1	0.95	0.8508
CSF1_HUMAN	Macrophage colony-stimulating factor 1	4	180.1	1	5	201.8	0.97	0.8316
CBG_HUMAN	Corticosteroid-binding globulin	8	412	0.93	7	342.1	1.03	0.8134
6PGL_HUMAN	6-phosphogluconolactonase	11	415.8	0.96	5	229.6	1	0.7962
NOV_HUMAN	Protein NOV homolog	2	76.6	1.28	1	28.4	0.68	0.7436
CADH6_HUMAN	Cadherin-6	2	163.8	1.03	1	44.2	0.92	0.7478
LFA3_HUMAN	Lymphocyte function-associated antigen 3	2	82.4	1.02	2	105.2	0.93	0.7348
TFF2_HUMAN	Trefoil factor 2	2	205.7	1.14	4	304.5	0.81	0.7562
DDAH2_HUMAN	N(G),N(G)-dimethylarginine dimethylaminohydrolase 2	2	83.5	1.01	5	237.4	0.94	0.7218
OSTP_HUMAN	Osteopontin	18	1199.4	1	15	1012	0.95	0.7133
KV305_HUMAN	Ig kappa chain V-III region WOL	6	510.4	0.98	6	520.6	0.96	0.693
IGKC_HUMAN	Ig kappa chain C region	14	1237.2	0.89	14	1080.7	1.05	0.7175
BT2A2_HUMAN	Butyrophilin subfamily 2 member A2	4	240.9	0.99	3	161.6	0.94	0.6292
NID1_HUMAN	Nidogen-1	3	105.7	1.22	2	81.8	0.7	0.6927

Table S5. *Cont.*

ACCESSION	Protein Name	WHOLE 1 #Peptides	WHOLE 1 Score	WHOLE 1 H/L	WHOLE 2 #Peptides	WHOLE 2 Score	WHOLE 2 H/L	*p* Value
1433Z_HUMAN	14-3-3 protein zeta/delta	6	337.2	0.79	8	495.2	1.14	0.6906
CD63_HUMAN	CD63 antigen	2	76.8	0.82	1	55.2	1.1	0.6769
TPIS_HUMAN	Triosephosphate isomerase	7	455.2	1.06	3	156.2	0.85	0.6118
EPCR_HUMAN	Endothelial protein C receptor	6	417.8	0.96	6	373.4	0.95	0.5347
GPVI_HUMAN	Platelet glycoprotein VI	2	107.1	0.96	1	60.6	0.95	0.5347
RNAS2_HUMAN	Non-secretory ribonuclease	6	533	0.95	8	508.9	0.95	0.528
VNN3_HUMAN	Vascular non-inflammatory molecule 3	2	152.8	0.93	2	91.9	0.97	0.5165
CAPG_HUMAN	Macrophage-capping protein	2	58	0.93	2	79.9	0.98	0.5142
LAYN_HUMAN	Layilin	2	65.7	0.96	1	39.1	0.94	0.5032
GOLM1_HUMAN	Golgi membrane protein 1	6	422.4	1.26	8	447.2	0.64	0.6437
IGHA2_HUMAN	Ig alpha-2 chain C region	16	1019.8	0.89	16	967.8	1.01	0.5183
PGK1_HUMAN	Phosphoglycerate kinase 1	2	169.6	0.6	1	34.9	1.29	0.624
PROM1_HUMAN	Prominin-1	2	63.9	0.93	2	64.5	0.95	0.4539
MSLN_HUMAN	Mesothelin	2	120.3	0.83	1	45.8	1.03	0.4643
LAMP2_HUMAN	Lysosome-associated membrane glycoprotein 2	3	144.8	0.99	1	29.3	0.86	0.386
ARF3_HUMAN	ADP-ribosylation factor 3	2	158.1	0.81	2	124.8	1.04	0.451
AATC_HUMAN	Aspartate aminotransferase, cytoplasmic	2	61.7	0.76	1	36.5	1.09	0.5072
HGFA_HUMAN	Hepatocyte growth factor activator	1	42.2	0.89	4	126.4	0.95	0.3211
CPN2_HUMAN	Carboxypeptidase N subunit 2	4	171.5	0.87	7	390	0.97	0.332
TRFL_HUMAN	Lactotransferrin	50	3270.7	0.93	50	3049.5	0.9	0.2912
KV310_HUMAN	Ig kappa chain V-III region VH (Fragment)	3	98.8	0.84	4	111.3	0.99	0.3544
HSP71_HUMAN	Heat shock 70 kDa protein 1A/1B	5	229.8	0.83	6	286.2	1	0.363

Table S5. *Cont.*

ACCESSION	Protein Name	WHOLE 1 #Peptides	WHOLE 1 Score	WHOLE 1 H/L	WHOLE 2 #Peptides	WHOLE 2 Score	WHOLE 2 H/L	*p* Value
PRIO_HUMAN	Major prion protein	2	115.7	1.05	2	105.6	0.78	0.4295
NTF2_HUMAN	Nuclear transport factor 2	2	157	0.95	2	153.9	0.88	0.2983
CASPE_HUMAN	Caspase-14	3	205.5	0.81	4	140.4	1.02	0.3806
KV117_HUMAN	Ig kappa chain V-I region Scw	5	552.3	0.91	5	448.4	0.92	0.2569
SEPP1_HUMAN	Selenoprotein P	2	85	0.64	2	82	1.18	0.5071
CADM1_HUMAN	Cell adhesion molecule 1	4	276.7	0.81	4	189.5	0.99	0.3143
CALM_HUMAN	Calmodulin	6	296	0.81	3	181.1	0.99	0.3143
WFDC2_HUMAN	WAP four-disulfide core domain protein 2	5	345	1	6	303	0.78	0.3037
CLN5_HUMAN	Ceroid-lipofuscinosis neuronal protein 5	4	134.4	0.72	2	72.2	1.06	0.3843
SDK1_HUMAN	Protein sidekick-1	1	28.6	0.84	1	29.6	0.94	0.2095
IGHG4_HUMAN	Ig gamma-4 chain C region	19	1144.1	0.86	15	844.9	0.92	0.1832
CATD_HUMAN	Cathepsin D	15	861.9	0.96	11	602.6	0.81	0.234
SPRL1_HUMAN	SPARC-like protein 1	4	174.4	0.85	3	115.7	0.92	0.1733
6PGD_HUMAN	6-phosphogluconate dehydrogenase, decarboxylating	5	239.5	0.69	3	147.4	1.07	0.3745
PEBP1_HUMAN	Phosphatidylethanolamine-binding protein 1	7	469.2	0.89	4	203.1	0.87	0.1503
A2GL_HUMAN	Leucine-rich alpha-2-glycoprotein	12	888	0.93	12	750.9	0.82	0.1807
YIPF3_HUMAN	Protein YIPF3	2	116.9	1.01	2	116.9	0.74	0.2871
PGAM1_HUMAN	Phosphoglycerate mutase 1	2	104	0.79	1	29.9	0.96	0.2152
ACTB_HUMAN	Actin, cytoplasmic 1	18	1239	0.79	20	1102.5	0.94	0.1756
ANXA3_HUMAN	Annexin A3	5	257.1	0.86	7	378.3	0.87	0.1193
KV122_HUMAN	Ig kappa chain V-I region BAN	2	156	0.89	2	177.8	0.84	0.123
CHMP5_HUMAN	Charged multivesicular body protein 5	1	37.1	0.84	1	51.5	0.88	0.1209

Table S5. *Cont.*

ACCESSION	Protein Name	WHOLE 1 #Peptides	WHOLE 1 Score	WHOLE 1 H/L	WHOLE 2 #Peptides	WHOLE 2 Score	WHOLE 2 H/L	*p* Value
GPX3_HUMAN	Glutathione peroxidase 3	6	220.1	0.78	5	171.6	0.95	0.1812
MUC20_HUMAN	Mucin-20	1	38.4	0.81	1	39.4	0.91	0.1345
TRFE_HUMAN	Serotransferrin	89	5734.5	0.9	87	5598.7	0.82	0.1252
FREM2_HUMAN	FRAS1-related extracellular matrix protein 2	2	96	0.67	1	34.7	1.05	0.3162
CBPE_HUMAN	Carboxypeptidase E	3	133.2	0.79	3	122.9	0.92	0.1503
PGRP2_HUMAN	N-acetylmuramoyl-L-alanine amidase	13	776.5	0.95	10	743.3	0.74	0.1808
F16P1_HUMAN	Fructose-1,6-bisphosphatase 1	3	173.2	1.1	3	163	0.58	0.35
ACTN4_HUMAN	Alpha-actinin-4	4	174.7	0.73	3	178	0.95	0.1794
ML12B_HUMAN	Myosin regulatory light chain 12B	2	94.4	0.7	1	39.2	0.98	0.209
CAH1_HUMAN	Carbonic anhydrase 1	4	186.5	0.65	2	81.3	1.02	0.2682
FBLN3_HUMAN	EGF-containing fibulin-like extracellular matrix protein 1	18	1112	0.86	14	904.3	0.82	0.0829
LAC_HUMAN	Ig lambda chain C regions	10	677.2	0.94	9	672.4	0.73	0.1642
ENOA_HUMAN	Alpha-enolase	6	349	0.75	4	245.7	0.92	0.1369
AFAM_HUMAN	Afamin	8	545.6	0.79	10	559.1	0.88	0.0962
PCOC1_HUMAN	Procollagen C-endopeptidase enhancer 1	2	53.8	0.84	4	179.6	0.81	0.0667
MOES_HUMAN	Moesin	8	317.1	0.79	4	167.5	0.85	0.066
CALR_HUMAN	Calreticulin	1	50.4	0.76	1	50.4	0.88	0.0842
A1BG_HUMAN	Alpha-1B-glycoprotein	16	947.9	0.8	13	660.1	0.82	0.0559
CA056_HUMAN	Uncharacterized protein C1orf56	2	160.5	0.87	1	81.5	0.75	0.0769
KIRR1_HUMAN	Kin of IRRE-like protein 1	1	36.6	0.76	2	67	0.86	0.0707
OLFM4_HUMAN	Olfactomedin-4	10	511.9	0.79	8	388.8	0.82	0.0531

Table S5. *Cont.*

ACCESSION	Protein Name	WHOLE 1 #Peptides	WHOLE 1 Score	WHOLE 1 H/L	WHOLE 2 #Peptides	WHOLE 2 Score	WHOLE 2 H/L	*p* Value
UFO_HUMAN	Tyrosine-protein kinase receptor UFO	10	502	1	8	342.4	0.61	0.2212
CO6A3_HUMAN	Collagen alpha-3(VI) chain	3	141.8	0.89	2	134.6	0.72	0.0924
LCAT_HUMAN	Phosphatidylcholine-sterol acyltransferase	7	399.1	0.77	8	355.5	0.82	0.0442
FBLN5_HUMAN	Fibulin-5	2	99.2	0.72	3	125.8	0.85	0.0583
COMP_HUMAN	Cartilage oligomeric matrix protein	4	182.5	0.88	2	59.2	0.68	0.0849
KV204_HUMAN	Ig kappa chain V-II region TEW	7	480.3	0.86	6	459.1	0.7	0.0687
CLUS_HUMAN	Clusterin	15	956.3	0.81	15	909.2	0.74	0.0404
BASP1_HUMAN	Brain acid soluble protein 1	2	82.2	0.77	3	102.4	0.78	0.0336
GSTP1_HUMAN	Glutathione S-transferase P	1	44.4	0.61	1	38.7	0.94	0.1484
EFNB1_HUMAN	Ephrin-B1	1	33.8	0.72	1	45.4	0.82	0.0448
LV301_HUMAN	Ig lambda chain V-III region SH	3	240.4	0.79	4	229.9	0.75	0.034
ANXA1_HUMAN	Annexin A1	14	889	0.75	11	851.8	0.79	0.0334
ACY1_HUMAN	Aminoacylase-1	6	289.2	1.01	5	197.5	0.52	0.2178
ARP2_HUMAN	Actin-related protein 2	2	95.2	0.97	2	129.6	0.55	0.1825
COF1_HUMAN	Cofilin-1	4	220.6	0.73	2	102.8	0.76	0.024
KV309_HUMAN	Ig kappa chain V-III region VG (Fragment)	2	81.1	0.79	2	79.8	0.7	0.0293
KV112_HUMAN	Ig kappa chain V-I region Kue	1	41.5	0.65	1	37.5	0.82	0.0488
FCGBP_HUMAN	IgGFc-binding protein	16	719	0.77	11	503.4	0.71	0.024
CERU_HUMAN	Ceruloplasmin	56	3336.9	0.7	49	2918	0.76	0.0227
1433E_HUMAN	14-3-3 protein epsilon	3	124.9	0.92	3	132	0.55	0.1302
CD59_HUMAN	CD59 glycoprotein	5	264.2	0.69	5	275.6	0.77	0.0246
TETN_HUMAN	Tetranectin	5	206.5	0.71	6	245.7	0.75	0.0199
VASN_HUMAN	Vasorin	12	816.7	0.71	11	700.8	0.74	0.0186

Table S5. *Cont.*

ACCESSION	Protein Name	WHOLE 1 #Peptides	WHOLE 1 Score	WHOLE 1 H/L	WHOLE 2 #Peptides	WHOLE 2 Score	WHOLE 2 H/L	*p* Value
AACT_HUMAN	Alpha-1-antichymotrypsin	20	951.6	0.63	17	920.6	0.82	0.0482
DERM_HUMAN	Dermatopontin	2	84.3	0.79	3	143.7	0.65	0.0316
PPAL_HUMAN	Lysosomal acid phosphatase	5	269.5	0.69	4	217.9	0.74	0.0179
ARPC2_HUMAN	Actin-related protein 2/3 complex subunit 2	2	76.4	0.58	2	94.6	0.85	0.073
TCPQ_HUMAN	T-complex protein 1 subunit theta	3	139.4	0.8	1	48.4	0.62	0.0437
IGHG2_HUMAN	Ig gamma-2 chain C region	18	1103.7	0.78	21	1318.9	0.63	0.0303
KAIN_HUMAN	Kallistatin	4	185.8	0.65	2	81.6	0.74	0.0182
LTBP1_HUMAN	Latent-transforming growth factor beta-binding protein 1	2	61.1	1.02	1	40.6	0.37	0.2102
HV103_HUMAN	Ig heavy chain V-I region V35	4	257.5	0.7	3	166.8	0.67	0.0123
CFAI_HUMAN	Complement factor I	12	829.3	0.67	9	557.7	0.69	0.0116
VTNC_HUMAN	Vitronectin	10	564.8	0.69	10	648.4	0.65	0.0111
KV119_HUMAN	Ig kappa chain V-I region Wes	2	244.8	0.69	2	207.3	0.65	0.0111
GAS6_HUMAN	Growth arrest-specific protein 6	6	305.2	0.81	6	226.3	0.52	0.0628
APOE_HUMAN	Apolipoprotein E	14	840	0.68	10	578	0.65	0.0103
TGON2_HUMAN	Trans-Golgi network integral membrane protein 2	2	93.3	0.79	1	46	0.54	0.0466
ISLR_HUMAN	Immunoglobulin superfamily containing leucine-rich repeat protein	5	207.8	0.68	1	28.5	0.64	0.0101
PPIA_HUMAN	Peptidyl-prolyl cis-trans isomerase A	1	58.3	0.63	1	38.4	0.69	0.0113
ILEU_HUMAN	Leukocyte elastase inhibitor	2	80.6	0.68	2	81.1	0.59	0.0112
HV102_HUMAN	Ig heavy chain V-I region HG3	2	111.3	0.66	1	60.7	0.6	0.0087
HV320_HUMAN	Ig heavy chain V-III region GAL	3	138.4	0.62	8	456.9	0.65	0.0072
COTL1_HUMAN	Coactosin-like protein	2	51	0.59	1	34.9	0.65	0.0081

Table S5. *Cont.*

ACCESSION	Protein Name	WHOLE 1 #Peptides	WHOLE 1 Score	WHOLE 1 H/L	WHOLE 2 #Peptides	WHOLE 2 Score	WHOLE 2 H/L	*p* Value
TS101_HUMAN	Tumor susceptibility gene 101 protein	1	34.2	1.05	1	53.1	0.19	0.211
ALBU_HUMAN	Serum albumin	137	8947.4	0.61	132	8355.1	0.62	0.0057
GELS_HUMAN	Gelsolin	19	1106.9	0.53	18	874.6	0.68	0.0158
KV203_HUMAN	Ig kappa chain V-II region MIL	5	304.4	0.55	3	172.9	0.65	0.0086
KV106_HUMAN	Ig kappa chain V-I region EU	4	300.8	0.75	5	353.9	0.45	0.047
IC1_HUMAN	Plasma protease C1 inhibitor	15	951.6	0.63	13	807.8	0.54	0.0072
THRB_HUMAN	Prothrombin	19	1381	0.6	18	1165	0.56	0.0047
IGHG3_HUMAN	Ig gamma-3 chain C region	21	1280.4	0.62	19	1132.7	0.54	0.0063
DNS2A_HUMAN	Deoxyribonuclease-2-alpha	3	142.9	0.76	2	70.6	0.39	0.061
DKK3_HUMAN	Dickkopf-related protein 3	1	65.4	0.6	2	135.8	0.55	0.0049
HBB_HUMAN	Hemoglobin subunit beta	16	897.1	0.56	17	869.1	0.58	0.0039
CYTC_HUMAN	Cystatin-C	7	443	0.55	5	423.6	0.58	0.0042
DNAS1_HUMAN	Deoxyribonuclease-1	7	342	0.74	4	219.2	0.39	0.0536
VSIG4_HUMAN	V-set and immunoglobulin domain-containing protein 4	2	59.8	0.55	1	49.8	0.55	0.0032
ITIH4_HUMAN	Inter-alpha-trypsin inhibitor heavy chain H4	21	1182.1	0.54	15	859.2	0.55	0.0033
ABP1_HUMAN	Amiloride-sensitive amine oxidase [copper-containing]	4	184.2	0.63	1	35.8	0.42	0.0175
A1AT_HUMAN	Alpha-1-antitrypsin	33	1950.7	0.53	36	1993	0.5	0.0027
IGHG1_HUMAN	Ig gamma-1 chain C region	24	1444.5	0.51	22	1408.7	0.52	0.0024
LKHA4_HUMAN	Leukotriene A-4 hydrolase	4	227.5	0.51	3	150.1	0.48	0.0023
CAP7_HUMAN	Azurocidin	5	205.5	0.52	2	78.8	0.42	0.0045
KLK3_HUMAN	Prostate-specific antigen	5	233	0.5	6	280.6	0.42	0.0031
C1S_HUMAN	Complement C1s subcomponent	2	144.7	0.53	2	88.3	0.28	0.0182

Table S6. Proteins in both AAL eluate duplicates for pTa v control urine experiment). Proteins are sorted according to average H/L ratio (pTa/control).

ACCESSION	Protein Name	AAL 1 #Peptides	AAL 1 Scores	AAL 1 H/L	AAL 2 #Peptides	AAL 2 Scores	AAL 2 H/L	*P* Value
VDAC1_HUMAN	Voltage-dependent anion-selective channel protein 1	3	148.7	11.57	2	83.9	4.03	1E-04
TGM4_HUMAN	Protein-glutamine gamma-glutamyltransferase 4	7	405.7	6.75	2	139	6.74	7E-07
MUC5B_HUMAN	Mucin-5B	4	310.1	5.16	10	572.2	6.04	2E-06
BPIL1_HUMAN	Bactericidal/permeability-increasing protein-like 1	1	39.4	5.63	1	41	5.13	2E-06
PPAP_HUMAN	Prostatic acid phosphatase	6	366.8	3.81	6	300.2	3.62	7E-06
UROM_HUMAN	Uromodulin	37	2099	4.54	32	1883	2.78	7E-05
CAH1_HUMAN	Carbonic anhydrase 1	4	230.4	2.67	2	106.7	4.47	9E-05
SAMP_HUMAN	Serum amyloid P-component	1	85.6	2.83	3	191.8	4.3	5E-05
MUC1_HUMAN	Mucin-1	2	108.3	3.35	1	46.2	3.3	1E-05
GSLG1_HUMAN	Golgi apparatus protein 1	9	407.3	2.94	10	480.4	3.63	2E-05
LPLC1_HUMAN	Long palate, lung and nasal epithelium carcinoma-associated protein 1	2	115.8	2.55	2	79.4	3.25	5E-05
BROX_HUMAN	BRO1 domain-containing protein BROX	1	34.5	5	1	31.4	0.67	0.187
ENPL_HUMAN	Endoplasmin	3	129.9	2.18	2	96.9	3.47	2E-04
CEL_HUMAN	Bile salt-activated lipase	2	71.1	3.19	2	71.9	2.03	3E-04
IGHG2_HUMAN	Ig gamma-2 chain C region	7	429.4	2.53	7	421.8	2.55	5E-05
KV402_HUMAN	Ig kappa chain V-IV region Len	2	182	2.5	1	73.6	2.57	6E-05
DPEP1_HUMAN	Dipeptidase 1	7	379.3	2.39	5	269.5	2.68	7E-05
DNS2A_HUMAN	Deoxyribonuclease-2-alpha	1	37.4	2.82	2	64.8	2.15	1E-04
PDC6I_HUMAN	Programmed cell death 6-interacting protein	1	103.6	0.9	2	135.1	3.9	0.083
CAH2_HUMAN	Carbonic anhydrase 2	3	161.8	2.66	3	184	2.08	2E-04
SPB4_HUMAN	Serpin B4	3	176.1	2.15	3	146.8	2.53	1E-04
ACY1_HUMAN	Aminoacylase-1	8	397.6	1.92	6	261.3	2.72	4E-04
PTGDS_HUMAN	Prostaglandin-H2 D-isomerase	1	41.8	2.85	1	42.2	1.73	1E-03
NAPSA_HUMAN	Napsin-A	4	250.6	2.28	6	287.4	2	2E-04
SPHM_HUMAN	N-sulphoglucosamine sulphohydrolase	3	193.6	2.25	2	57.2	2.03	2E-04
AT1A1_HUMAN	Sodium/potassium-transporting ATPase subunit alpha-1	3	182.1	2.56	2	88	1.72	9E-04
HV102_HUMAN	Ig heavy chain V-I region HG3	1	56	2.9	1	56.2	1.34	0.009
ITIH4_HUMAN	Inter-alpha-trypsin inhibitor heavy chain H4	7	372.3	1.61	6	333.4	2.5	0.001
MDR1_HUMAN	Multidrug resistance protein 1	4	160.1	2.36	2	73	1.73	7E-04

Table S6. *Cont.*

ACCESSION	Protein Name	AAL 1 #Peptides	AAL 1 Scores	AAL 1 H/L	AAL 2 #Peptides	AAL 2 Scores	AAL 2 H/L	*P* Value
PGRP1_HUMAN	Peptidoglycan recognition protein 1	1	53.6	2.09	3	148.9	1.97	3E-04
TPP1_HUMAN	Tripeptidyl-peptidase 1	1	70.5	1.78	2	122.6	2.27	5E-04
NEP_HUMAN	Neprilysin	16	828.9	2.27	12	487.6	1.76	6E-04
GNAS2_HUMAN	Guanine nucleotide-binding protein G(s) subunit alpha isoforms short	3	152.4	1.99	2	57.3	2.01	3E-04
A2GL_HUMAN	Leucine-rich alpha-2-glycoprotein	7	445.2	2.14	5	332.2	1.84	4E-04
MMRN2_HUMAN	Multimerin-2	3	177.1	2.18	2	64.6	1.78	5E-04
PERM_HUMAN	Myeloperoxidase	3	100.3	2.32	5	211.6	1.61	0.001
CETP_HUMAN	Cholesteryl ester transfer protein	2	94.9	1.96	1	68.5	1.96	4E-04
MASP2_HUMAN	Mannan-binding lectin serine protease 2	6	466.4	1.96	5	456.8	1.95	4E-04
IGHG3_HUMAN	Ig gamma-3 chain C region	10	596.1	1.86	10	498.2	1.99	4E-04
PGBM_HUMAN	Basement membrane-specific heparan sulfate proteoglycan core protein	2	83.2	1.26	3	128.1	2.58	0.013
A1BG_HUMAN	Alpha-1B-glycoprotein	4	200.1	1.74	2	115.3	2.09	7E-04
R4RL2_HUMAN	Reticulon-4 receptor-like 2	1	42	1.93	2	154	1.85	5E-04
CBPE_HUMAN	Carboxypeptidase E	1	37.7	2.05	2	71.3	1.63	0.001
BGAL_HUMAN	Beta-galactosidase	9	500.6	1.71	5	267.9	1.95	8E-04
NAGAB_HUMAN	Alpha-N-acetylgalactosaminidase	5	249.4	1.99	3	117.3	1.66	1E-03
CADH1_HUMAN	Cadherin-1	5	403.3	1.71	6	352.2	1.91	9E-04
A2ML1_HUMAN	Alpha-2-macroglobulin-like protein 1	5	247.6	2.07	6	278.1	1.53	0.002
PPAL_HUMAN	Lysosomal acid phosphatase	12	612.4	1.89	9	353.1	1.66	0.001
SAP3_HUMAN	Ganglioside GM2 activator	2	154.9	2.91	2	133.6	0.64	0.365
IGHG1_HUMAN	Ig gamma-1 chain C region	11	630.9	1.89	10	438.4	1.63	0.001
APOD_HUMAN	Apolipoprotein D	15	1052	2.11	13	958	1.39	0.005
CSPG4_HUMAN	Chondroitin sulfate proteoglycan 4	1	78.2	1.63	2	156	1.85	0.001
ALDOB_HUMAN	Fructose-bisphosphate aldolase B	13	893.3	1.95	11	747.4	1.51	0.002
AQP2_HUMAN	Aquaporin-2	2	102.7	1.75	2	108	1.71	0.001
K22E_HUMAN	Keratin, type II cytoskeletal 2 epidermal	28	1821	1.66	29	1727	1.78	0.001
UROK_HUMAN	Urokinase-type plasminogen activator	1	43.2	1.82	1	28.4	1.59	0.002

Table S6. *Cont.*

ACCESSION	Protein Name	AAL 1 #Peptides	AAL 1 Scores	AAL 1 H/L	AAL 2 #Peptides	AAL 2 Scores	AAL 2 H/L	*P* Value
ANXA4_HUMAN	Annexin A4	3	195	1.98	2	56.2	1.41	0.004
ANX11_HUMAN	Annexin A11	3	107.2	1.14	3	106.7	2.22	0.028
AMPN_HUMAN	Aminopeptidase N	45	2539	1.7	44	2486	1.6	0.002
DPP2_HUMAN	Dipeptidyl peptidase 2	5	345.2	1.67	5	288.4	1.61	0.002
CD14_HUMAN	Monocyte differentiation antigen CD14	8	369.3	1.62	6	291.6	1.62	0.002
CMBL_HUMAN	Carboxymethylenebutenolidase homolog	1	32.1	1.78	1	26.4	1.46	0.004
DDC_HUMAN	Aromatic-L-amino-acid decarboxylase	1	82.2	1.69	2	149.3	1.54	0.002
PBLD_HUMAN	Phenazine biosynthesis-like domain-containing protein	1	59.3	1.72	1	57.1	1.5	0.003
IPSP_HUMAN	Plasma serine protease inhibitor	25	1498	1.64	24	1170	1.54	0.003
RAB14_HUMAN	Ras-related protein Rab-14	1	31.5	1.63	1	61.2	1.53	0.003
GTR5_HUMAN	Solute carrier family 2, facilitated glucose transporter member 5	2	86.6	1.46	1	30.2	1.68	0.004
PTPRG_HUMAN	Receptor-type tyrosine-protein phosphatase gamma	2	62.6	1.61	1	34.6	1.52	0.003
SLC31_HUMAN	Neutral and basic amino acid transport protein rBAT	3	127.5	1.64	3	141.3	1.48	0.004
CNDP2_HUMAN	Cytosolic non-specific dipeptidase	6	237.8	1.68	4	158.4	1.41	0.005
YS019_HUMAN	Transmembrane protein HSPC323	3	155.1	1.59	2	71.5	1.5	0.004
PSCA_HUMAN	Prostate stem cell antigen	6	445.4	1.4	6	421.5	1.68	0.005
PRDX6_HUMAN	Peroxiredoxin-6	3	136.6	1.61	2	105.5	1.47	0.004
GSTA1_HUMAN	Glutathione S-transferase A1	1	28.5	1.7	1	48.4	1.36	0.007
B3GN1_HUMAN	N-acetyllactosaminide beta-1,3-N-acetylglucosaminyltransferase	3	246.3	1.61	2	158.5	1.44	0.005
LEG9_HUMAN	Galectin-9	2	124.2	1.59	1	36.7	1.45	0.005
K2C1_HUMAN	Keratin, type II cytoskeletal 1	49	3072	1.21	45	2583	1.82	0.019
GSTM3_HUMAN	Glutathione S-transferase Mu 3	3	136.7	1.71	2	81.7	1.32	0.009
BHMT1_HUMAN	Betaine--homocysteine S-methyltransferase 1	7	519.6	1.6	8	446.9	1.43	0.005
CQ101_HUMAN	PKHD domain-containing transmembrane protein C17orf101	1	42	1.5	1	30.2	1.53	0.005
IDHC_HUMAN	Isocitrate dehydrogenase [NADP] cytoplasmic	7	363	1.41	9	444	1.61	0.006

Table S6. *Cont.*

ACCESSION	Protein Name	AAL 1 #Peptides	AAL 1 Scores	AAL 1 H/L	AAL 2 #Peptides	AAL 2 Scores	AAL 2 H/L	*P* Value
K1C9_HUMAN	Keratin, type I cytoskeletal 9	21	1304	1.18	23	1253	1.83	0.024
DHSO_HUMAN	Sorbitol dehydrogenase	1	41	1.69	1	49.6	1.31	0.01
MA2A1_HUMAN	Alpha-mannosidase 2	2	92.2	1.45	1	25.2	1.55	0.006
CATC_HUMAN	Dipeptidyl peptidase 1	2	100	1.38	2	97	1.61	0.007
EZRI_HUMAN	Ezrin	5	240	1.36	1	31	1.63	0.008
XPP2_HUMAN	Xaa-Pro aminopeptidase 2	3	178.6	1.85	3	141.6	1.14	0.033
CATO_HUMAN	Cathepsin O	1	36.6	1.63	1	39.2	1.34	0.009
HBB_HUMAN	Hemoglobin subunit beta	12	795.1	1.54	10	519.5	1.42	0.007
1433T_HUMAN	14-3-3 protein theta	3	209.7	1.42	1	56.8	1.52	0.007
NEUR1_HUMAN	Sialidase-1	4	196.5	1.39	2	84.7	1.54	0.008
PCKGC_HUMAN	Phosphoenolpyruvate carboxykinase, cytosolic [GTP]	2	67.8	1.31	1	44.6	1.63	0.011
STOM_HUMAN	Erythrocyte band 7 integral membrane protein	1	31.6	1.56	1	31.1	1.35	0.009
IGKC_HUMAN	Ig kappa chain C region	7	526.1	1.51	7	556.4	1.41	0.008
CBG_HUMAN	Corticosteroid-binding globulin	8	450	1.5	6	277	1.41	0.008
K6PL_HUMAN	6-phosphofructokinase, liver type	1	43.5	1.48	2	64.4	1.42	0.008
THBG_HUMAN	Thyroxine-binding globulin	17	1069	1.43	8	466.3	1.47	0.008
K1C10_HUMAN	Keratin, type I cytoskeletal 10	25	1445	1.41	25	1341	1.47	0.009
DPP4_HUMAN	Dipeptidyl peptidase 4	24	1298	1.48	23	1241	1.39	0.01
LAC_HUMAN	Ig lambda chain C regions	6	327.5	1.52	5	269.1	1.34	0.011
BHMT2_HUMAN	Betaine--homocysteine S-methyltransferase 2	2	112.4	1.57	3	115.2	1.28	0.015
AMPE_HUMAN	Glutamyl aminopeptidase	31	1489	1.51	27	1246	1.33	0.013
GLYC_HUMAN	Serine hydroxymethyltransferase, cytosolic	4	282.3	1.52	5	253	1.31	0.014
HS90B_HUMAN	Heat shock protein HSP 90-beta	13	769.7	1.26	10	482.5	1.55	0.018
DERM_HUMAN	Dermatopontin	1	45.5	1.9	1	56	0.91	0.173
COFA1_HUMAN	Collagen alpha-1(XV) chain	4	201.9	1.44	1	68.4	1.36	0.013
4F2_HUMAN	4F2 cell-surface antigen heavy chain	8	350.9	1.36	6	288.5	1.44	0.013
SCRB2_HUMAN	Lysosome membrane protein 2	5	232.8	1.76	2	106.4	1.03	0.085
BDH2_HUMAN	3-hydroxybutyrate dehydrogenase type 2	2	109.9	1.37	2	94.5	1.41	0.014
PODXL_HUMAN	Podocalyxin-like protein 1	5	264.8	1.69	4	265.8	1.05	0.075
FUCO2_HUMAN	Plasma alpha-L-fucosidase	4	177.3	1.42	2	70.2	1.32	0.018
ASSY_HUMAN	Argininosuccinate synthase	4	172.2	1.41	1	35.2	1.32	0.018
BODG_HUMAN	Gamma-butyrobetaine dioxygenase	3	149	1.47	2	81.2	1.24	0.025

Table S6. *Cont.*

ACCESSION	Protein Name	AAL 1 #Peptides	AAL 1 Scores	AAL 1 H/L	AAL 2 #Peptides	AAL 2 Scores	AAL 2 H/L	*P* Value
ACE2_HUMAN	Angiotensin-converting enzyme 2	2	123.9	1.15	3	145.1	1.54	0.043
UBIQ_HUMAN	Ubiquitin	3	159.6	1.4	2	81	1.28	0.023
AQP1_HUMAN	Aquaporin-1	1	83.8	1.42	2	108.6	1.26	0.026
F16P1_HUMAN	Fructose-1,6-bisphosphatase 1	3	175.8	1.16	2	133.8	1.52	0.043
CD9_HUMAN	CD9 antigen	1	28.3	1.33	2	76.7	1.31	0.027
TSP1_HUMAN	Thrombospondin-1	4	255.7	1.45	5	231.7	1.19	0.039
L1CAM_HUMAN	Neural cell adhesion molecule L1	3	133.2	1.17	1	55.6	1.46	0.046
TSN1_HUMAN	Tetraspanin-1	2	115	1.32	1	31.4	1.28	0.033
LG3BP_HUMAN	Galectin-3-binding protein	18	1164	1.26	25	1466	1.34	0.034
AK1A1_HUMAN	Alcohol dehydrogenase [NADP+]	1	31.4	1.19	1	39.2	1.41	0.044
FTHFD_HUMAN	10-formyltetrahydrofolate dehydrogenase	4	166.2	1.46	2	95.4	1.13	0.061
TBA1C_HUMAN	Tubulin alpha-1C chain	5	227.7	1.35	4	167.8	1.22	0.043
HV306_HUMAN	Ig heavy chain V-III region BUT	2	142.9	1.23	2	199.6	1.34	0.045
VASN_HUMAN	Vasorin	9	554.6	0.97	9	515.2	1.57	0.167
HBA_HUMAN	Hemoglobin subunit alpha	5	234.4	1.44	3	193.9	1.1	0.08
LYAG_HUMAN	Lysosomal alpha-glucosidase	22	1410	1.21	25	1359	1.33	0.052
IST1_HUMAN	IST1 homolog	1	38.3	1.2	1	52.2	1.34	0.053
ANTR1_HUMAN	Anthrax toxin receptor 1	2	128.9	1.32	1	65.6	1.18	0.067
CPVL_HUMAN	Probable serine carboxypeptidase CPVL	8	386.6	1.34	7	285.9	1.15	0.078
6PGL_HUMAN	6-phosphogluconolactonase	4	192	1.12	5	186.7	1.36	0.087
KNG1_HUMAN	Kininogen-1	24	1702	1.2	22	1334	1.28	0.071
PLD3_HUMAN	Phospholipase D3	1	66.3	1.05	3	142.1	1.42	0.126
GNS_HUMAN	N-acetylglucosamine-6-sulfatase	4	198.9	1.46	1	67.2	1.01	0.16
CATD_HUMAN	Cathepsin D	9	394	1.31	9	318.3	1.16	0.084
PGRP2_HUMAN	N-acetylmuramoyl-L-alanine amidase	5	219.2	0.89	5	239.9	1.54	0.308
KV301_HUMAN	Ig kappa chain V-III region B6	1	109.8	1.18	2	199.7	1.21	0.115
PPGB_HUMAN	Lysosomal protective protein	3	196.1	1.25	3	191.8	1.14	0.127
AMBP_HUMAN	Protein AMBP	16	1149	1.17	19	1202	1.22	0.122
ASAH1_HUMAN	Acid ceramidase	4	172.9	1.24	3	116.3	1.14	0.133
ANAG_HUMAN	Alpha-N-acetylglucosaminidase	13	745.2	1.19	12	679.4	1.16	0.141
LDHB_HUMAN	L-lactate dehydrogenase B chain	12	577.1	1.23	7	301.6	1.12	0.157

Table S6. *Cont.*

ACCESSION	Protein Name	AAL 1 #Peptides	AAL 1 Scores	AAL 1 H/L	AAL 2 #Peptides	AAL 2 Scores	AAL 2 H/L	*P* Value
GDIB_HUMAN	Rab GDP dissociation inhibitor beta	2	113.7	1.18	5	180	1.16	0.157
SPB5_HUMAN	Serpin B5	2	104.7	1.14	1	37.2	1.2	0.161
AACT_HUMAN	Alpha-1-antichymotrypsin	13	811.1	1.26	13	687.9	1.07	0.193
BCAM_HUMAN	Basal cell adhesion molecule	4	259.6	1.22	4	202.8	1.11	0.174
PROZ_HUMAN	Vitamin K-dependent protein Z	5	187.8	1.13	10	402.1	1.19	0.178
MUC4_HUMAN	Mucin-4	1	58.2	1.09	2	68.8	1.23	0.194
AGAL_HUMAN	Alpha-galactosidase A	7	437.6	1.11	6	304.5	1.21	0.195
GP1BA_HUMAN	Platelet glycoprotein Ib alpha chain	2	159.1	1.09	1	37.3	1.22	0.21
AL9A1_HUMAN	4-trimethylaminobutyraldehyde dehydrogenase	4	145.1	1.25	3	106.6	1.03	0.268
GAS6_HUMAN	Growth arrest-specific protein 6	5	298.5	0.86	6	250.7	1.41	0.517
FAT2_HUMAN	Protocadherin Fat 2	1	34.9	1.18	1	27.3	1.09	0.261
ENOA_HUMAN	Alpha-enolase	6	344.6	1.28	8	462.9	0.97	0.359
1433Z_HUMAN	14-3-3 protein zeta/delta	4	274.7	1.22	3	216.4	1.03	0.301
MXRA8_HUMAN	Matrix-remodeling-associated protein 8	4	168	1.26	3	131.5	0.97	0.383
ARP2_HUMAN	Actin-related protein 2	3	174.2	1.31	3	169.4	0.9	0.543
CLN5_HUMAN	Ceroid-lipofuscinosis neuronal protein 5	4	189.4	1.2	4	210.7	1	0.411
HS90A_HUMAN	Heat shock protein HSP 90-alpha	14	809.7	1.19	11	514.3	1	0.426
LRP2_HUMAN	Low-density lipoprotein receptor-related protein 2	136	8235	1.04	137	8129	1.15	0.4
EGF_HUMAN	Pro-epidermal growth factor	47	2708	1.2	39	2186	0.97	0.485
IGHA1_HUMAN	Ig alpha-1 chain C region	21	1314	1.1	17	1214	1.08	0.424
CERU_HUMAN	Ceruloplasmin	36	2196	1.07	27	1466	1.1	0.426
CAPG_HUMAN	Macrophage-capping protein	2	73.8	0.96	1	38.8	1.18	0.559
ANPRC_HUMAN	Atrial natriuretic peptide receptor 3	1	32.1	0.95	1	68.8	1.2	0.578
RAB1A_HUMAN	Ras-related protein Rab-1A	4	146.6	0.92	2	55	1.22	0.613
CPN2_HUMAN	Carboxypeptidase N subunit 2	15	831.4	1.08	14	792.8	1.06	0.514
G3P_HUMAN	Glyceraldehyde-3-phosphate dehydrogenase	13	615.8	1.12	8	404.9	1.01	0.557
DHAK_HUMAN	Bifunctional ATP-dependent dihydroxyacetone kinase/FAD-AMP lyase (cyclizing)	1	30.7	1.05	2	84.1	1.08	0.542
TMBI1_HUMAN	Transmembrane BAX inhibitor motif-containing protein 1	1	38.3	1.1	1	27.2	1.03	0.571

Table S6. *Cont.*

**ACCESSION**	**Protein Name**	**AAL 1 #Peptides**	**AAL 1 Scores**	**AAL 1 H/L**	**AAL 2 #Peptides**	**AAL 2 Scores**	**AAL 2 H/L**	***P* Value**
CYTC_HUMAN	Cystatin-C	1	34.6	0.88	2	131.8	1.24	0.733
GGT1_HUMAN	Gamma-glutamyltranspeptidase 1	13	693.4	1.02	10	482.8	1.09	0.603
CATH_HUMAN	Cathepsin H	3	167.4	1.13	2	140.3	0.97	0.651
LAMP1_HUMAN	Lysosome-associated membrane glycoprotein 1	4	278.9	0.87	4	210.9	1.23	0.786
CAN1_HUMAN	Calpain-1 catalytic subunit	1	34.1	1.09	1	36.2	1.01	0.657
AFAM_HUMAN	Afamin	2	116.9	0.96	3	148.6	1.13	0.691
PGK1_HUMAN	Phosphoglycerate kinase 1	4	226.2	0.99	4	156	1.09	0.695
CUBN_HUMAN	Cubilin	95	6328	0.97	97	6093	1.1	0.732
PAPP2_HUMAN	Pappalysin-2	3	169	0.94	3	116.6	1.13	0.789
DESP_HUMAN	Desmoplakin	2	87.6	1.18	1	27	0.88	0.866
TBB5_HUMAN	Tubulin beta chain	2	162.9	1.09	2	140.5	0.97	0.783
FUCO_HUMAN	Tissue alpha-L-fucosidase	4	194.8	1.01	3	123.9	1.04	0.802
S36A2_HUMAN	Proton-coupled amino acid transporter 2	6	262.1	1.39	5	247.5	0.65	0.771
LMAN2_HUMAN	Vesicular integral-membrane protein VIP36	7	393.1	1.08	7	347.4	0.95	0.913
FBLN4_HUMAN	EGF-containing fibulin-like extracellular matrix protein 2	3	134.8	1.06	1	33	0.97	0.907
UBA1_HUMAN	Ubiquitin-like modifier-activating enzyme 1	4	171.2	1.04	3	99.7	0.99	0.897
TM7S3_HUMAN	Transmembrane 7 superfamily member 3	2	140.2	1.02	2	117.2	1.01	0.9
PCP_HUMAN	Lysosomal Pro-X carboxypeptidase	6	295	1.14	1	57.4	0.88	0.993
MA1A1_HUMAN	Mannosyl-oligosaccharide 1,2-alpha-mannosidase IA	6	345.2	1	6	255.2	1.02	0.932
CTL4_HUMAN	Choline transporter-like protein 4	2	66.1	1	2	70.5	1.02	0.932
CO6A1_HUMAN	Collagen alpha-1(VI) chain	11	812.5	0.99	9	494.4	1.03	0.935
ILEU_HUMAN	Leukocyte elastase inhibitor	3	157.9	1.06	1	38.8	0.95	0.976
HSP71_HUMAN	Heat shock 70 kDa protein 1A/1B	6	286.2	0.99	7	323.3	1.02	0.967
PSA7_HUMAN	Proteasome subunit alpha type-7	2	53.3	0.89	2	57.9	1.11	0.946
TRFE_HUMAN	Serotransferrin	11	757.9	1.06	10	534.4	0.93	0.955
GRHPR_HUMAN	Glyoxylate reductase/hydroxypyruvate reductase	2	129.1	1.04	2	77	0.95	0.96
CD320_HUMAN	CD320 antigen	2	112.5	0.57	1	25.3	1.41	0.617
ACTN4_HUMAN	Alpha-actinin-4	3	168.7	1.03	2	73.8	0.96	0.927

Table S6. *Cont.*

ACCESSION	Protein Name	AAL 1 #Peptides	AAL 1 Scores	AAL 1 H/L	AAL 2 #Peptides	AAL 2 Scores	AAL 2 H/L	*P* Value
C1RL_HUMAN	Complement C1r subcomponent-like protein	3	133.6	0.98	4	143.9	1	0.929
CD81_HUMAN	CD81 antigen	2	115.6	0.95	2	128.1	1.03	0.922
SDC1_HUMAN	Syndecan-1	1	52.7	0.88	2	76.6	1.1	0.885
CRYL1_HUMAN	Lambda-crystallin homolog	2	59.1	1.13	1	25.5	0.82	0.755
CBR1_HUMAN	Carbonyl reductase [NADPH] 1	1	34.2	1	1	63.5	0.95	0.795
TIMD3_HUMAN	Hepatitis A virus cellular receptor 2	1	67	1.03	3	154.8	0.91	0.758
HV305_HUMAN	Ig heavy chain V-III region BRO	3	223.1	1	3	237	0.94	0.762
MA1C1_HUMAN	Mannosyl-oligosaccharide 1,2-alpha-mannosidase IC	1	29.7	0.99	1	48	0.95	0.761
SODE_HUMAN	Extracellular superoxide dismutase [Cu-Zn]	2	144.2	0.65	3	163.1	1.28	0.605
ICOSL_HUMAN	IC	2	74	1.02	3	155.8	0.91	0.728
KAIN_HUMAN	Kallistatin	7	351.6	0.99	5	181	0.94	0.728
IGHA2_HUMAN	Ig alpha-2 chain C region	14	958.5	0.98	12	858.3	0.95	0.727
APOE_HUMAN	Apolipoprotein E	11	615.8	0.96	11	604.1	0.97	0.727
VTNC_HUMAN	Vitronectin	6	389.7	1.11	8	469	0.81	0.676
ATRN_HUMAN	Attractin	20	1343	1	23	1383	0.92	0.696
FREM2_HUMAN	FRAS1-related extracellular matrix protein 2	7	380.6	1.01	6	269.1	0.9	0.634
A1AG1_HUMAN	Alpha-1-acid glycoprotein 1	4	206.4	0.78	4	241.9	1.12	0.6
CD44_HUMAN	CD44 antigen	3	143.9	0.81	1	38	1.09	0.584
TCPQ_HUMAN	T-complex protein 1 subunit theta	2	122.7	0.99	1	41.9	0.9	0.573
H4_HUMAN	Histone H4	2	130	0.59	4	201.5	1.29	0.484
PTPRJ_HUMAN	Receptor-type tyrosine-protein phosphatase eta	2	76.6	0.95	3	127.1	0.92	0.514
BTD_HUMAN	Biotinidase	17	873.2	0.93	12	583.9	0.94	0.514
LRC19_HUMAN	Leucine-rich repeat-containing protein 19	4	146.3	0.88	4	169	0.99	0.514
LDHA_HUMAN	L-lactate dehydrogenase A chain	8	362	0.85	4	211.7	1.02	0.513
FBLN3_HUMAN	EGF-containing fibulin-like extracellular matrix protein 1	11	587.4	0.76	10	417.5	1.1	0.506
MYH9_HUMAN	Myosin-9	15	817.2	1.01	7	338	0.85	0.494
ANGL2_HUMAN	Angiopoietin-related protein 2	2	94.2	1	2	89.7	0.86	0.493
PVR_HUMAN	Poliovirus receptor	5	257.6	0.95	5	265.2	0.91	0.488
MGA_HUMAN	Maltase-glucoamylase, intestinal	33	1721	0.94	29	1364	0.91	0.462

Table S6. *Cont.*

ACCESSION	Protein Name	AAL 1 #Peptides	AAL 1 Scores	AAL 1 H/L	AAL 2 #Peptides	AAL 2 Scores	AAL 2 H/L	*P* Value
K1C17_HUMAN	Keratin, type I cytoskeletal 17	6	398.9	0.61	7	344	1.23	0.441
IC1_HUMAN	Plasma protease C1 inhibitor	20	1351	0.84	18	1140	1	0.436
F151A_HUMAN	Protein FAM151A	2	108.6	0.95	4	211.1	0.89	0.413
SUSD2_HUMAN	Sushi domain-containing protein 2	7	571.3	0.87	6	480.3	0.97	0.409
ROBO4_HUMAN	Roundabout homolog 4	11	598.3	0.9	9	553.7	0.92	0.385
ACE_HUMAN	Angiotensin-converting enzyme	8	377.3	0.9	10	455.8	0.92	0.385
ABP1_HUMAN	Amiloride-sensitive amine oxidase [copper-containing]	5	371.6	1.07	9	638	0.75	0.395
ISLR_HUMAN	Immunoglobulin superfamily containing leucine-rich repeat protein	3	201.6	1.04	1	58.1	0.78	0.39
DCD_HUMAN	Dermcidin	4	185	0.52	3	136.2	1.3	0.376
H2B1K_HUMAN	Histone H2B type 1-K	2	69.7	0.91	1	37.7	0.91	0.364
TTYH3_HUMAN	Protein tweety homolog 3	1	52	0.86	2	67.2	0.96	0.364
PLMN_HUMAN	Plasminogen	1	30.1	0.82	1	41.4	0.99	0.371
K1C14_HUMAN	Keratin, type I cytoskeletal 14	14	800	0.63	12	601.2	1.18	0.388
ACPH_HUMAN	Acylamino-acid-releasing enzyme	1	58.3	1	1	41.5	0.81	0.359
PPIB_HUMAN	Peptidyl-prolyl cis-trans isomerase B	2	157	1.05	2	133.2	0.74	0.339
ALBU_HUMAN	Serum albumin	31	2011	0.96	41	2180	0.82	0.294
FGL2_HUMAN	Fibroleukin	8	343	0.93	8	351	0.85	0.285
OLFM4_HUMAN	Olfactomedin-4	19	1179	0.93	15	822.5	0.84	0.269
NRP1_HUMAN	Neuropilin-1	1	34.9	0.72	1	30.7	1.05	0.3
GANAB_HUMAN	Neutral alpha-glucosidase AB	2	124.6	0.94	1	50.4	0.83	0.255
PCD24_HUMAN	Protocadherin-24	15	1026	0.93	17	1147	0.84	0.252
MVP_HUMAN	Major vault protein	2	102.4	0.9	1	26.1	0.84	0.218
MMRN1_HUMAN	Multimerin-1	1	83.6	1.37	2	91.5	0.36	0.256
CEAM7_HUMAN	Carcinoembryonic antigen-related cell adhesion molecule 7	1	45.3	0.96	2	84.8	0.77	0.195
MEGF8_HUMAN	Multiple epidermal growth factor-like domains protein 8	10	584.2	0.95	10	531.3	0.78	0.192
A2MG_HUMAN	Alpha-2-macroglobulin	33	1701	0.89	25	1245	0.83	0.177
FETUA_HUMAN	Alpha-2-HS-glycoprotein	4	150	0.82	1	56.2	0.9	0.175
SORL_HUMAN	Sortilin-related receptor	6	331.3	0.82	10	418.7	0.91	0.177
MADCA_HUMAN	Mucosal addressin cell adhesion molecule 1	1	101	0.8	1	87.5	0.91	0.155
K2C5_HUMAN	Keratin, type II cytoskeletal 5	27	1548	0.69	19	993.7	1.01	0.192

Table S6. *Cont.*

ACCESSION	Protein Name	AAL 1 #Peptides	AAL 1 Scores	AAL 1 H/L	AAL 2 #Peptides	AAL 2 Scores	AAL 2 H/L	*P* Value
CHM2A_HUMAN	Charged multivesicular body protein 2a	2	100.3	0.91	1	26.7	0.78	0.151
PROM1_HUMAN	Prominin-1	1	55	0.81	3	135	0.87	0.122
FAT4_HUMAN	Protocadherin Fat 4	8	357.5	0.81	8	353.8	0.85	0.104
KPYM_HUMAN	Pyruvate kinase isozymes M1/M2	13	634.9	0.81	8	371.3	0.84	0.097
DNAS1_HUMAN	Deoxyribonuclease-1	4	280.1	0.81	3	175.5	0.84	0.097
CLM9_HUMAN	CMRF35-like molecule 9	4	215.8	0.68	4	193.3	0.97	0.13
MPRI_HUMAN	Cation-independent mannose-6-phosphate receptor	1	43.6	0.87	2	59.1	0.76	0.082
VNN3_HUMAN	Vascular non-inflammatory molecule 3	2	148.1	0.82	2	92.8	0.8	0.076
PGM1_HUMAN	Phosphoglucomutase-1	5	311	0.84	4	179.8	0.78	0.073
KLK1_HUMAN	Kallikrein-1	4	239.1	0.72	4	167.4	0.89	0.077
HV304_HUMAN	Ig heavy chain V-III region TIL	3	294.1	0.77	3	176.5	0.83	0.061
QSOX1_HUMAN	Sulfhydryl oxidase 1	3	168.3	0.81	3	122.8	0.78	0.056
DDAH2_HUMAN	N(G),N(G)-dimethylarginine dimethylaminohydrolase 2	5	248.6	0.8	5	150.3	0.79	0.056
CADH6_HUMAN	Cadherin-6	2	91.6	0.76	1	40.7	0.83	0.057
MUC18_HUMAN	Cell surface glycoprotein MUC18	2	96.6	0.75	2	79.9	0.84	0.06
ANT3_HUMAN	Antithrombin-III	6	294.1	0.73	3	170.3	0.86	0.063
MDHC_HUMAN	Malate dehydrogenase, cytoplasmic	3	129.5	0.91	4	161.9	0.66	0.072
PGAM1_HUMAN	Phosphoglycerate mutase 1	5	209.8	0.87	2	63.8	0.7	0.055
GLCM_HUMAN	Glucosylceramidase	1	59.2	0.75	1	37	0.81	0.045
PNPH_HUMAN	Purine nucleoside phosphorylase	1	60.4	0.68	4	162.6	0.88	0.055
PCD12_HUMAN	Protocadherin-12	3	170.8	0.79	4	197.8	0.76	0.038
FCN2_HUMAN	Ficolin-2	3	169.4	0.84	1	40.2	0.66	0.035
CFAB_HUMAN	Complement factor B	17	888.3	0.71	17	830.5	0.78	0.025
VTDB_HUMAN	Vitamin D-binding protein	3	170.1	0.83	1	41.5	0.66	0.03
CF072_HUMAN	Uncharacterized protein C6orf72	1	46.7	0.73	1	25.5	0.75	0.02
CO6A3_HUMAN	Collagen alpha-3(VI) chain	4	191.4	0.61	1	50.6	0.86	0.034
CAD15_HUMAN	Cadherin-15	2	143.6	0.75	2	69.3	0.71	0.017
TGON2_HUMAN	Trans-Golgi network integral membrane protein 2	1	83.7	0.73	3	107.6	0.73	0.017
MXRA5_HUMAN	Matrix-remodeling-associated protein 5	3	153	0.81	2	91.9	0.65	0.021

Table S6. *Cont.*

ACCESSION	Protein Name	AAL 1 #Peptides	AAL 1 Scores	AAL 1 H/L	AAL 2 #Peptides	AAL 2 Scores	AAL 2 H/L	*P* Value
CYTB_HUMAN	Cystatin-B	1	40.2	0.65	1	31	0.8	0.02
LRRN4_HUMAN	Leucine-rich repeat neuronal protein 4	2	158.7	0.71	1	67.9	0.73	0.014
HSPB1_HUMAN	Heat shock protein beta-1	3	201.6	0.68	2	145.4	0.75	0.014
PIGR_HUMAN	Polymeric immunoglobulin receptor	24	1515	0.74	26	1587	0.69	0.013
ANGT_HUMAN	Angiotensinogen	6	409	0.69	6	396.3	0.72	0.011
AMNLS_HUMAN	Protein amnionless	2	82.1	0.61	1	31	0.8	0.017
CATB_HUMAN	Cathepsin B	2	100.9	0.82	4	156.5	0.59	0.021
TIG1_HUMAN	Retinoic acid receptor responder protein 1	3	223.8	0.65	5	229.8	0.74	0.01
CSF1_HUMAN	Macrophage colony-stimulating factor 1	1	35.2	0.62	2	79.6	0.77	0.012
SDC4_HUMAN	Syndecan-4	1	35.7	0.65	1	30.4	0.73	0.009
OSTP_HUMAN	Osteopontin	1	29.6	0.72	1	33.9	0.65	0.008
6PGD_HUMAN	6-phosphogluconate dehydrogenase, decarboxylating	6	306	0.66	6	394.4	0.71	0.007
PCDGK_HUMAN	Protocadherin gamma-C3	2	100.9	0.66	3	158.4	0.7	0.007
TRFL_HUMAN	Lactotransferrin	1	52.7	0.57	6	315.5	0.78	0.012
P3IP1_HUMAN	Phosphoinositide-3-kinase-interacting protein 1	1	61.4	0.52	2	87.6	0.84	0.02
PEDF_HUMAN	Pigment epithelium-derived factor	12	778	0.67	11	539.1	0.68	0.006
VMO1_HUMAN	Vitelline membrane outer layer protein 1 homolog	2	87.2	0.7	1	44.3	0.64	0.006
CFAI_HUMAN	Complement factor I	1	43.6	0.6	3	130.6	0.74	0.008
HEMO_HUMAN	Hemopexin	2	100.7	0.71	4	163.8	0.6	0.006
PDIA1_HUMAN	Protein disulfide-isomerase	1	61.6	0.7	1	35.3	0.61	0.005
ITIH2_HUMAN	Inter-alpha-trypsin inhibitor heavy chain H2	6	394.2	0.57	5	263.5	0.74	0.007
IGJ_HUMAN	Immunoglobulin J chain	1	87.1	0.54	1	100.8	0.78	0.01
PYGB_HUMAN	Glycogen phosphorylase, brain form	2	100.3	0.64	1	63.3	0.66	0.004
CAB45_HUMAN	45 kDa calcium-binding protein	1	74.7	0.6	2	115.8	0.68	0.004
FSTL1_HUMAN	Follistatin-related protein 1	2	83.1	0.73	2	93.4	0.54	0.006
A1AT_HUMAN	Alpha-1-antitrypsin	24	1400	0.64	22	1103	0.6	0.002
GOLM1_HUMAN	Golgi membrane protein 1	2	109	0.62	3	122.7	0.62	0.002
ANXA1_HUMAN	Annexin A1	11	649.4	0.56	10	480.8	0.68	0.003
PLSL_HUMAN	Plastin-2	3	130.3	0.52	2	75.1	0.72	0.005

Table S6. *Cont.*

ACCESSION	Protein Name	AAL 1 #Peptides	AAL 1 Scores	AAL 1 H/L	AAL 2 #Peptides	AAL 2 Scores	AAL 2 H/L	*P* Value
LYAM1_HUMAN	L-selectin	2	84	0.65	4	183.1	0.59	0.002
S10A8_HUMAN	Protein S100-A8	2	78.9	0.77	2	79.4	0.46	0.01
GELS_HUMAN	Gelsolin	3	126.3	0.55	4	147.6	0.67	0.003
FIBG_HUMAN	Fibrinogen gamma chain	12	638.2	0.65	9	570.3	0.56	0.002
HEG1_HUMAN	Protein HEG homolog 1	7	434.5	0.63	9	524.1	0.58	0.002
ATF6B_HUMAN	Cyclic AMP-dependent transcription factor ATF-6 beta	1	59.1	0.56	1	51.8	0.65	0.002
FIBB_HUMAN	Fibrinogen beta chain	18	1007	0.61	18	868.6	0.57	0.001
HEXA_HUMAN	Beta-hexosaminidase subunit alpha	2	76.6	0.56	1	27.3	0.55	8E-04
ITIH1_HUMAN	Inter-alpha-trypsin inhibitor heavy chain H1	2	143.6	0.51	1	90.5	0.57	7E-04
LUM_HUMAN	Lumican	7	390	0.55	5	202.9	0.5	5E-04
LIPL_HUMAN	Lipoprotein lipase	1	57	0.56	2	87.8	0.44	5E-04

Table S7. Patient Information. The patients used for pools C1 and C2 had no disease detected. The patients used for pool C3 were diagnosed with cystitis and pool C4 comprised 5 patients with cystitis and 4 patients with inflammation. We show the number of male & female patients, the number of patients with grade 1, 2 or 3 disease and mean ages and urinary protein (Bradford) and creatinine (Jaffe) concentrations (both in μg/mL). Equal volumes of individual patient urines were mixed to generate each pooled sample.

Pool	No. Patients	No. Male/Female	Age (years)	Stage	Grade (G1/G2/G3)	Protein (μg/mL)	Creatinine (μg/mL)
C1	12	10/2	69	NA	NA	91	1595
C2	12	9/3	69	NA	NA	109	2346
C3	9	7/2	67	NA	NA	83	1685
C4	9	7/2	63	NA	NA	95	1777
Ta1	10	6/4	71	pTa	4/6/0	76	1321
Ta2	10	6/4	74	pTa	4/6/0	109	1707
T1-1	9	6/3	74	pT1	1/2/6	187	1935
T1-2	9	7/2	72	pT1	0/5/4	176	1972
T2-1	8	8/0	73	pT2+	0/1/7	171	1626
T2-2	8	6/2	69	pT2+	0/0/3	156	1478
Control	28	22/6	69	NA	NA	151	1789
pTa	75	55/20	71	pTa	35/35/5	185	1756
LG-pTa	36	27/9	70	pTa	36/0/0	148	1983
